# Explaining population booms and busts in Mid-Holocene Europe

**DOI:** 10.1038/s41598-023-35920-z

**Published:** 2023-06-08

**Authors:** Dániel Kondor, James S. Bennett, Detlef Gronenborn, Nicolas Antunes, Daniel Hoyer, Peter Turchin

**Affiliations:** 1 Complexity Science Hub, Vienna, Austria; 2 University of Washington, Seattle, WA USA; 3 LEIZA (Leibniz Zentrum für Archäologie), Mainz, Germany; 4 Evolution Institute, Tampa, FL USA; 5 George Brown College, Toronto, Canada

**Keywords:** Computational science, Population dynamics, Sustainability

## Abstract

Archaeological evidence suggests that the population dynamics of Mid-Holocene (Late Mesolithic to Initial Bronze Age, ca. 7000–3000 BCE) Europe are characterized by recurrent booms and busts of regional settlement and occupation density. These boom-bust patterns are documented in the temporal distribution of 14C dates and in archaeological settlement data from regional studies. We test two competing hypotheses attempting to explain these dynamics: climate forcing and social dynamics leading to inter-group conflict. Using the framework of spatially-explicit agent-based models, we translated these hypotheses into a suite of explicit computational models, derived quantitative predictions for population fluctuations, and compared these predictions to data. We demonstrate that climate variation during the European Mid-Holocene is unable to explain the quantitative features (average periodicities and amplitudes) of observed boom-bust dynamics. In contrast, scenarios with social dynamics encompassing density-dependent conflict produce population patterns with time scales and amplitudes similar to those observed in the data. These results suggest that social processes, including violent conflict, played a crucial role in the shaping of population dynamics of European Mid-Holocene societies.

## Introduction

**Figure 1:**
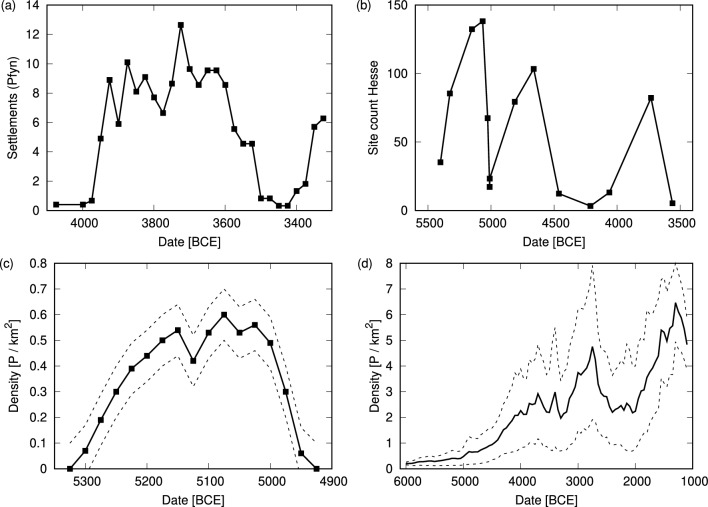
Archaeological proxies for population dynamics in western and central Europe during the Mid-Holocene. (**a**) Settlement counts associated with the Pfyn culture, data from^10^ (**b**) The number of occupied sites in central Germany (Hesse), data from^13^; (**c**) Estimated population density in the lower Rhine region, data from^14^; (**d**) Long-term reconstruction of population density in north-central Switzerland, data from^6^. Dashed lines in panels (**c**) and (**d**) represent the range of uncertainty given by the authors in the original publications.

During the Mid-Holocene (ca. 7000–3000 BCE) early farmers expanded from Western Anatolia and spread throughout Europe into a world of hunter-gatherers^1^. Archaeological evidence of regional and local settlement patterns has established the existence of regional population booms and busts during this period^2–6^ (see examples in Fig. 1). Analysis of these data reveals a consistent pattern, in which most regions undergo a well-characterized population boom after the arrival of the first farmers, followed by a decline within a few hundred years. On local and regional scales archaeological evidence indicates that settlements grew (in numbers and size) over the course of several generations before declining or even being abandoned^7–11^. This initial dynamic was followed throughout the Neolithic into the Bronze Age by recurrent regional population booms and busts, superimposed on an overall increasing population trend^5,12^.

**Figure 2:**
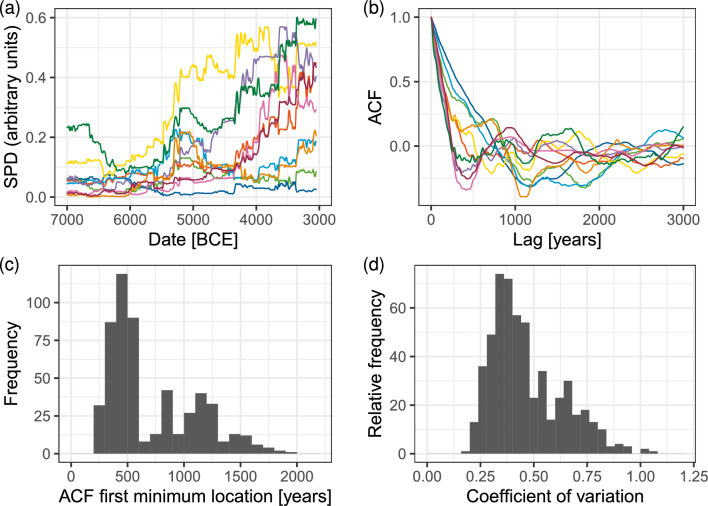
Cyclic patterns in European Mid-Holocene radiocarbon data. (**a**) Examples of SPD time series in different regions. (**b**) Autocorrelation functions (ACF) for these regional SPD time series. (**c**) Aggregate frequency distribution of the first minimum lag for ACFs from regions containing at least 500 data points over overlapping regions with a linear size of 500 km (n = 550). (**d**) Aggregate frequency distribution of coefficients of variations of SPD times series for these regions (n = 550). Additional regional SPDs and ACFs are displayed in Figs. S8 and S9 in the *SI Appendix*; disaggregated frequency distributions of ACF minima and CV values are displayed in Fig. S6 in the *SI Appendix*.

Boom and bust dynamics are also evident in large-scale radiocarbon (14C) data sets covering Mid-Holocene Europe. A statistical analysis of Summed Probability Distribution (SPD) of 14C data over different spatial scales (see Materials and Methods) suggests that regional boom and bust dynamics are characterized by long-term oscillations of characteristic periods and amplitudes (Fig. 2). In particular, half-cycle periods (estimated by the first minimum of the Autocorrelation Function, ACF) cluster around 500 years (Fig. 2b,c). Typical coefficients of variance (CV), a measure of oscillation amplitude, are 0.25 or more (Fig. 2d).

While much has been written on the collapse of centralized societies ranging from chiefdoms^15,16^ to large-scale states and empires^17–19^, mechanisms leading to recurrent population busts in small-scale farming societies have only recently been addressed^5,8,20–22^. One prominent hypothesis proposes changing climate as the major exogenous driver^23–26^. Other investigators argued that climate change had only limited effects on early farming societies^21,27–30^ and that booms and busts are mainly a result of endogenous processes instead^20^.

Such endogenous processes involve dynamical feedbacks between the affected populations and their environment (whether physical or social) and can be described as second-order dynamical models. Long studied in ecology, especially in the context of resource-consumer population interactions^31^, these models have not been fully appreciated when considering human populations^19,32^. A key component of such models is that population density interacts with another dynamic variable, waxing and waning on a similar time scale. Several possibilities for such interactions have been proposed. For example, several authors have developed models of populations interacting with renewable natural resources that can potentially generate sustained second-order cycles^33–36^. However, elsewhere^37^ we show that, contrary to these works, the dynamical interaction between farmers and soil nutrients cannot produce second-order cycles for a broad range of parameters that are consistent with temperate biomes such as within Neolithic Europe.

Another second-order process that can possibly explain the Mid-Holocene boom-bust cycles is the dynamic interaction between population numbers (or density) and the level of social cohesion. In this framework, high population densities can lead to conflicts and ultimately intensification of inter-personal violence^38^. Models focusing on these processes emphasize the reduced viability of communities in “disintegrative” phases^2,10,20,39,40^, often corroborated by evidence of reduced trade networks and growing inequality^9,11,22,41,42^. Disintegrative phases are often accompanied by an intensification of inter-personal violence, both within and between groups^23,43–47^. While intensity of inter-group conflicts exhibits substantial variation in time, several recent studies have linked settlement abandonment and population decline in different world regions to periods when intergroup conflict spikes^20,44,45,48^.

While hypotheses attempting to explain population dynamics in Mid-Holocene Europe have been much discussed, they have not yet been tested in a large-scale analysis that compares model predictions with data. Deriving quantitative predictions from explicit models is a necessary step when testing hypotheses about population dynamics because verbal reasoning may mislead^49^. Here we focus on the two rival (but not mutually-exclusive) hypotheses outlined above: population fluctuations in Mid-Holocene Europe were driven (1) by an exogenous process, climate change; or (2) by an endogenous process, density-dependent social disintegration and conflict. We have developed an agent-based model that incorporates the effect of climate and density-dependent conflict in an abstract representation and compare simulation results with the 14C data-derived statistics (Fig. 2). Our model does not assume these processes act independently of one another. However, by building the model in a flexible way that allows disabling the separate process components, we are able to test which are essential to reproducing observed demographic processes at the correct time scales. While 14C data is less accurate than indicators of settlement patterns compiled from archaeological reports, it allows us to perform a systematic analysis of temporal patterns across a broad study area (Europe between 7000 BCE and 3000 BCE) and perform quantitative comparisons with model outputs.

## Results

### Modeling population dynamics

**Figure 3:**
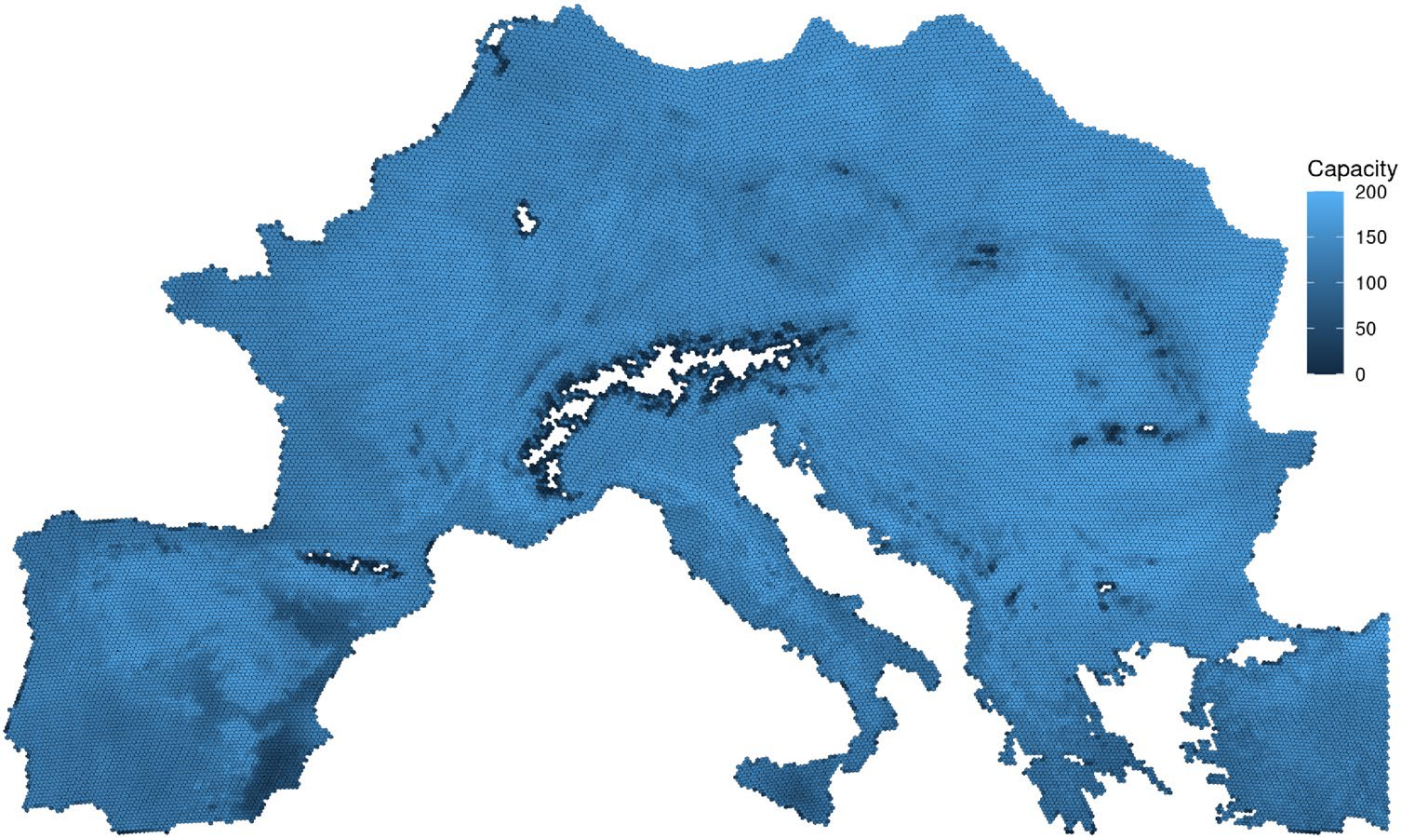
Illustration of the study area. Hexagon cells are colored according to their carrying capacity. The hexagon grid was created with the DGGRID software^50,51^ as described in the article text. Figure was generated with R (version 4.1.2, available at https://www.r-project.org/), using the ggplot2 package (version 3.3.5, available at https://ggplot2.tidyverse.org/).

To investigate the effect of the mechanisms outlined above, we developed a spatially-explicit agent-based model that tracks the spread of farming groups along with a set of possible interactions among them. The temporal focus of our investigation is the mid-Holocene (7000–3000 BCE), roughly the period from the first evidence of agriculture in Europe to the beginnings of the Bronze Age. The simulation space includes most of Europe, excluding northeastern areas where spread of agriculture occurred later than our period (Fig. 3). Our simulation represents this area as discrete units (“cells”) using an equal-area hexagon grid^50,51^. Each cell is either empty, or is occupied by an independent group of farmers (a “village”).

The simulation progresses in one-year discrete timesteps and has two components that are evaluated each year. In the population component, the number of farmers in a cell is modeled as a logistic growth process with carrying capacity that varies in space and time. Spatial variation is based on relative variability of agricultural productivity in Europe according FAO GAEZ^52,53^. Temporal variation is based on climate variability and was estimated with a combination of a crop yield emulator^54^ and a paleoclimate dataset^55^. These procedures are explained in more detail in the *Materials and Methods* and *SI Appendix*. Temporal variability of climate effects on yield is scalable with a simulation parameter, *s* (see *Materials and Methods* for more details). Setting $s = 0$ eliminates this variance and thus the effects of climate on the simulation.

In the interaction component of the simulation, the population of cells can split, migrate, and, optionally, engage in inter-group conflict. These events happen stochastically. For each cell, the probability of a population split increases with population size (see Materials and Methods, Eq. 2). The result of a split is that a fraction of the cell’s occupants attempts to migrate to a new location. The target cell is selected randomly, in a process influenced by distance, suitability for agriculture, and occupation status. Specifically, the probability of selecting an already occupied cell is given by $p_E$. Based on the value of $p_E$ and the resulting interactions, our model has two main variants:

1. If $p_E = 0$, only empty cells can be selected as targets. If no suitable empty cell is found, the split-off event is canceled (no migration occurs). In this variant, no conflict occurs and population numbers are solely affected by agricultural productivity and, via *s*, any variation to it due to climate.
2. If $p_E > 0$ and an occupied cell is selected as a target, it is assumed that a conflict between the cells ensues. Beyond the confrontation itself, with probability $p_C$, the group winning the conflict becomes “militarized” and will continue attacking other neighbors, with a yearly rate of $p_A$; we refer to such cells as “aggressors”. Subsequent attacks by aggressors can thus create additional aggressors causing conflict to spread. Aggressors revert back to peaceful farmers either spontaneously with rate $p_R$, or if they are unable to find a cell within range to attack. In this model variant, the number of aggressor cells (and thus the amount of violent conflict) acts as a dynamic variable: its level increases in response to high occupation density (with a rate that is determined by the $p_E$ parameter) and decreases at a relatively constant rate.

Note that in the case of $p_C = 0$ conflict occurs but aggressors are not created. In this case, conflict is not a dynamic variable (the model is first-order) and the level of violence is determined solely by the density of occupied cells.

Our model necessarily abstracts the interaction mechanisms to capture the essential demographic effects without representing the detailed social and ecological processes that show significant variation in the study period and area^1,56,57^. While our abstractions cannot capture the full richness of environmental and social interactions of the period, we believe they are overall consistent with archaeological knowledge.

Group fission and onward migration was an important aspect of at least the early Neolithic, and likely contributed to the brisk spread of agriculture in Europe^30,41,58^. Several authors have linked group fission and onward migration to competition over a limited set of local resources and as a way of avoiding intra-group conflicts^41,59^; we represent this by requiring that group fission probabilities increase with group size, consistent with “scalar stress” models as well^60,61^. However, founding a new agricultural settlement required a considerable effort and likely required several years of preparation, potentially including a “pilot” phase^62^, and involved a significant part of the group^58^. While we do not model these details directly, these considerations inform our model. Specifically, we limit the probability of split-off events so they occur roughly once every 8 years, and we choose the migrating group size to be roughly half of the population of the origin cell.

**Figure 4:**
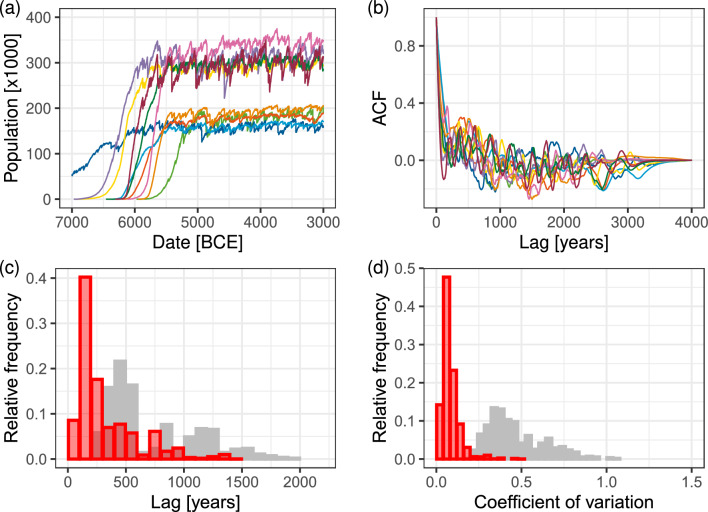
Simulated regional populations under climate variation ($s=2$) and no inter-group conflict ($p_E = 0$). (**a**) Example simulated population time series from different regions for one simulation realization and one grid position. (**b**) Autocorrelation functions (ACF) for each example (detrended) regional population time series (example time series and ACFs for a larger set of regions and additional *s* values are shown in Figs. S10–S11 in the *SI Appendix*). (**c**) Aggregate frequency distribution of the first minimum lag for ACFs over overlapping analysis grids of 500 km linear size (red). The distribution shows aggregate results of 100 repeated realizations of the simulation. Grey frequency distribution from observed radiocarbon data from Fig 2c. (**d**) Aggregate frequency distribution of coefficients of variations over overlapping grids in 100 simulation realizations (red). Grey frequency distribution from observed radiocarbon data from Fig 2d.

Similarly, our understanding of conflicts in small-scale societies informs our probabilistic modeling of these interactions. Based on ethnographic evidence, it is clear that most instances of conflict between small-scale societies and entailing inter-personal violence result only in a small number of casualties; nevertheless, ambushes and massacres happen, albeit infrequently^63,64^, with considerable evidence of such events in Mid-Holocene Europe^23,43–47^. Thus, the build-up to large-scale violent confrontations can take several years in which enmity is persistent. At the same time, repeated conflicts have been suggested to further limit population numbers by restricting settlements to defensible locations and the creation of “buffer zones” between groups, thus decreasing the effective carrying capacity of the landscape^49,65–67^.

In our model however, we abstract away the multitude of ways in which conflicts arise and affect population numbers and focus only on confrontations that either result in complete disintegration (when the attackers win), or in the creation of additional aggressors (when the defenders win); thus the $p_A$ parameter in the model represents how often lingering enmity escalates to such large-scale events. At the same time, although the end result is potentially the termination of a group, it does not imply that all individuals in it are killed; survivors may disperse as refugees to other settlements where they possibly have relatives or friends or are adopted into the winning group, initially as subordinate members^68,69^. We do not model such dispersal explicitly, instead simplifying this process by going directly to the end result (complete disintegration of one of the conflict parties or creation of more aggressors). In this manner, our model captures the essential dynamical feedback between population numbers and conflict, as increased levels of conflict directly lead to population declines.

All simulation parameters and their typical values are summarized in Table 2 in the Materials and Methods section. The simulation results below reflect sets of parameter combinations that are both representative of the behavior of model variants and plausible for Mid-Holocene Europe; an exploration of the larger parameter space is done in the *SI Appendix* (Figs. S14–S41).

### Climate forcing is unable to produce population cycles

In the first variant of the model there is no conflict between cells and regional populations fluctuate solely in response to the effect of climate variation on carrying capacity. As explained in the *SI*, we estimated the effect of climate on temporal variation in each simulation cell’s productivity by using historical climate data of Armstrong et al.^55^ and a yield emulator of Franke et al.^54^, which translated temperature and rainfall data into agricultural productivity in each cell for each year during the modeled period. This procedure yielded an average year-to-year coefficient of variation of $5.8\%$. Further, we investigated whether assuming an even stronger climate effect could result in boom-bust patterns similar to the observed ones. Specifically, we doubled, tripled and quadrupled the strength of the climate effect ($s = 2, 3$ and 4; see *Materials and Methods*, Eq. (1)). Here we report the results for $s = 2$, while the *SI Appendix* gives the results for $s = 1, 3$ and 4.

Figure 4 shows the results from a simulation with $s = 2$. Panel (a) shows example regional population trajectories (calculated for regions with a linear size of 500 km; see *Materials and Methods* for details). Supplementary Videos S1 and S2 show example model realizations for parameters $G = 40\,\textrm{km}$ and $s = 2,4$ respectiely. Model-predicted trajectories in Fig. 4a are roughly S-shaped with fluctuations around that trend, as would be expected from a noisy logistic growth process. Variation in the timing of peak populations is due to the arrival times of first farmers into a region. The (stochastic) equilibrium levels are primarily determined by the number of habitable cells in the region and their suitability for agriculture. The impact of climatic variation on population is visible as high frequency variation around the overall logistic pattern. Overall larger variations (i.e., higher values of the *s* parameter) result in lower total population (see the *SI Appendix*, Fig. S10). This is the result of larger climatic “shocks” causing more rapid population loss that can only be partially restored by population growth during climatic upturns. Such lower overall population numbers also translate into a slower spread of farming (as lower cell populations translate to lower chance of split-off and migration), an effect that can be compensated for by increasing the characteristic distance of migrations (see section 2 and Fig. S5 in the *SI Appendix*, also Supplementary Videos S1–S2).

We analyzed temporal patterns in the simulation results identically to the procedure used to characterize the radiocarbon data as shown in Fig. 2. The analysis shows no evidence for longer-term booms and busts. The ACFs show a sharp spike in the frequency distribution around 200 years (Fig. 4c), indicating the dominance of short-term fluctuations, compared to the peak of 500 years in the case of the radiocarbon data. At the same time, typical CV values (Fig. 4d) are below 0.2, significantly lower than the values estimated from the radiocarbon dataset. These results are robust with respect to variations in the *s* and other simulation parameters (see *SI Appendix*, Fig. S14).

These results suggest that the modeled effect of relatively high-frequency climate variations is unable to explain, on its own, the population dynamics observed in our study period. While climate downturns (or “shocks”) can result in significant population declines (via reduced carrying capacity), these ecological effects do not last long enough to result in the observed long population “busts” that typically last several hundred years in many regions. Additional possible effects of climate shocks, such as illness and decreased fertility due to poor nutrition, would also act only as short-term responses due to restored productivity and would not significantly alter these temporal dynamics. Thus, we turn to the model variant that involves long-term dynamical feedbacks, along with the effect of climate.

### Conflict produces population cycles

**Figure 5:**
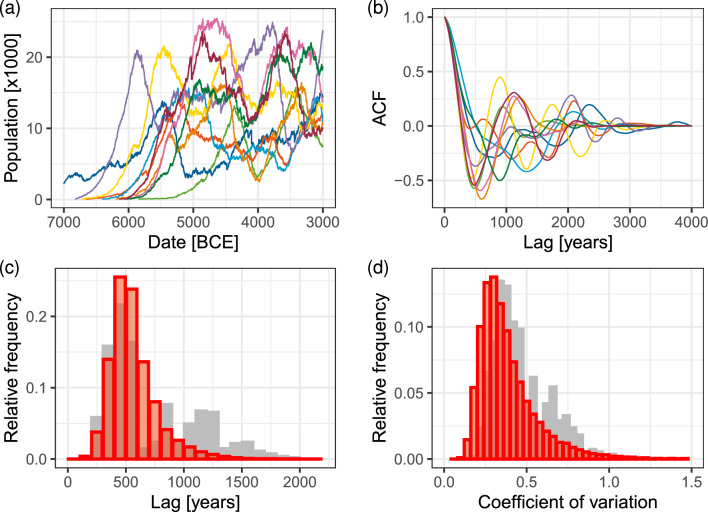
Cyclic patterns in simulated regional populations under inter-group conflict ($p_E = 1$; $p_A = 1/20$ years) and climate variation ($s=2$). (**a**) Example simulated population time series from different regions in one simulation realization and one grid position. (**b**) Autocorrelation functions (ACF) for each example (detrended) regional population time series (example time series and ACFs for a larger set of regions and additional s values are shown in Figs. S12–S13 in the SI Appendix). (**c**) Aggregate frequency distribution of the first minimum lag for ACFs over overlapping analysis grids of 500 km linear size among 100 repeated simulation realizations (red). Grey frequency distribution from observed radiocarbon data from Fig. 2c. (**d**) Aggregate frequency distribution of coefficients of variations over overlapping grids among 100 repeated simulation realizations. Grey frequency distribution from observed radiocarbon data from Fig. 2d. Additional parameters values are $G = 80\,\textrm{km}$, $B = 8\,\textrm{km}$, $s = 2$, $p_S = 0.5$, $p_C = 1$ and $p_R = 0.01$.

Our second model variant involves social dynamics with an intensification of violent confrontations and aggressor formation as a second-order dynamical process. As the population level in a region increases, “disintegration” leading to conflict is initiated with increasing frequency. High level of conflict, in turn, results in substantial probability of population extinction in cells, depressing overall population numbers. These second-order dynamics are clearly seen in Fig. 5a,b as noisy population cycles, where the noise in this case is due to strong high-frequency climate variance ($s=2$). Supplementary Videos S3 and S4 also show these patterns in example simulation realizations for parameter choices $(p_E = 1, p_A = 0.05)$ and $(p_E = 0.2, p_A = 0.1)$ respectively.

Analysis of the simulated trajectories shows that the dynamics predicted by the social disintegration/conflict model match the quantitative characteristics in the radiocarbon data (Fig. 5c for ACFs and Fig. 5d for CV). These results are robust to variations in the model parameters. A wide range of parameter combinations yield similar population dynamics (see *SI*; Fig. S15; also S29–S38). Overall, the location of ACF minima tends to decrease, while CV tends to increase with increasing $p_A$ or $p_E$. Half cycle lengths vary between 200 and 700 years for the range of parameters studied. Varying the characteristic migration distance has little impact on the frequency distribution, while decreasing the amplitude of climatic variations (*s*) shifts the ACF minimums more closely to the mode of the distribution (*SI* Fig. S16; see also Figs. S29–S38).

Comparing overall population levels (Figs. 4a and 5a), it is apparent that, recurrent conflict has a stronger effect on depressing regional population numbers than climate variation, typically by a factor of 6 under the nominal simulation parameters. While climatic downturns cause simulated population declines, they rarely result in large-scale depopulation, let alone empty areas that routinely form when conflict levels are high. Indeed, as can be seen in the accompanying videos, the depopulation of cells during conflict leads to large-scale spatial ’waves’ of conflict and accompanying empty regions sweeping through the simulation space (Supplementary Videos S3–S4). The spatial scale of these waves increases with decreasing frequency of attack ($p_A$). With $p_A \le 0.1$ (i.e., large-scale confrontations happen every 10 years on average) large scale ($1000~\textrm{km}^2$) spatial patterns are typical. Depending on the simulation parameters, conflict is triggered at different occupation densities. For larger values of $p_E$, conflict erupts when there is still ample available unsettled land, thus the landscape is never fully occupied over the course of the simulation. Our model of recurrent violent conflict, therefore, offers one possible explanation for the growing body of archaeological evidence that during the earlier Neolithic small, densely occupied areas were surrounded by zones of land suitable for agriculture, yet unsettled^7,9,14,67^.

To separate the impact of climate variation from the dynamics of density-dependent conflict, we explored a model variant where climate variations are assumed to have no effect on agricultural productivity ($s = 0$). The results (Figs. S29–S30 in the *SI Appendix*) show population cycles with nearly the same long-time scales. However, the distribution of half-cycle lengths is more sharply focused around its mode, indicating more regularity in the resulting dynamics. Thus, while climate variation alone appears unable to produce long-term population cycles, it can modulate the timing and length of conflict-induced cycles.

On the other hand, avoiding the formation of aggressors during conflict ($p_C = 0$) eliminates any second-order dynamics and long-term population cycles are not observed (Figs. S17; S39–S41 in the *SI Appendix*). As with the first model variant without conflict, ACF distributions show peaks below 300 years, along with peaks in CV distributions below 0.15. This demonstrates the importance of treating recurrent conflict as a dynamic process whose levels change at time scales comparable to demographic processes. It also suggests that any induced conflict due to increased competition for reduced resources during relatively short-term climate downturns is not sufficient for explaining long-term population cycles.

Our second model variant assumes that aggressors are stationary and attack farmers within a maximum radius from their location. We also investigated a variant in which aggressors are mobile, and relocate by taking over the cell of their victims (see *Materials and Methods*). Similar patterns of booms and busts in regional population appear; see *SI Appendix* (Figs. S19–28).

## Discussion

In this paper, we have investigated a suite of models for population dynamics in Mid-Holocene Europe. We model population spread, climate influence on agricultural productivity, and warfare. We show that climate variation alone is insufficient to explain the observed boom-bust dynamics, quantified using ACFs and CVs. In contrast, a model with inter-group conflict is capable of producing population oscillation patterns consistent with empirical data^2,3,9,10^. Different assumptions about the mechanisms of warfare (e.g., stationary versus mobile aggressors) and many parameter combinations result in second-order dynamics in which oscillations have periodicities and amplitudes similar to observed data.

Our results are consistent with qualitative material culture-based models of cyclical processes that have been used to explain interrelated changes in population numbers and social cohesion on regional scales^8,10,13,20^. Our modeled interactions between “farmers”, “migrants”, and “aggressors” are also able to produce recurrent unoccupied “empty spaces” around dense settlements, as also seen in the empirical evidence^7,14,67^.

Our results clearly suggest that population growth and decline dynamics in mid-Holocene Europe arise primarily from a second-order dynamical process. In this article, we have focused on an abstract representation of density-dependent conflict that emphasizes direct casualties. Other indirect demographic effects on migration and birth rates stemming from the loss of social cohesion can also lead to second-order oscillations. Further extensions of our model could allow disentangling underlying social processes in more detail, such as the effects of epidemics, reduced fertility, changed migratory behavior and subsistence patterns. Such extensions will require a careful review of existing archaeological evidence to be able to formulate testable hypotheses about the relative importance of each factor. The growing amount of ancient DNA (aDNA) evidence that shows population continuity, replacement, mixing and migration patterns^70–72^ will be especially valuable in testing quantitative predictions about the direct and indirect effects of inter-group conflicts.

While our results suggest that empirically observed boom-bust cycles in Mid-Holocene Europe arise primarily from endogenous dynamical processes (i.e., density-dependent conflict), this does not mean that climate did not play an important role for early farmers. Changes in climate affect productivity of agricultural systems and can cause famines, trigger conflict, or induce migration. But they cannot explain recurrent long-term population patterns that are observed in our data.

Our model provides a simple yet generic framework for density-dependent social conflict without relying on specific interaction patterns or mechanisms that would explain the nature and background of disintegrative factors. Model extensions that capture a richer set of within- and between interactions could allow drawing more direct comparisons with models of cultural integration and disintegration suggested for Neolithic populations^10,20,22,48,73^. Such extensions are likely to provide a better characterization of population densities, the scale of population declines, and perhaps the spatial extent of Mid-Holocene culture areas; they may also lend insight into the constitution and spread of societies based on farming, including indications of strong and systematic larger-scale cooperation as suggested by the sudden appearance of “mega-sites” and monuments during the 5th and 4th millennia BCE^74^. Such insights could have significant implications well beyond the demographics of early farmers.

## Methods

### Characterizing Mid-Holocene boom-bust dynamics with radiocarbon data

As we noted in the Introduction, boom-bust dynamics of Mid-Holocene populations are evidenced both by archaeological data on settlements numbers and sizes (the number of houses), and by 14C proxies. Because settlement data are only available for a limited number of regions (and periods), in our comprehensive empirical test of model predictions we focus on the 14C data, which are available for most of Europe, 7000–3000 BCE.

We used a database of curated radiocarbon dates provided by the c14bazAAR R package^75^ (version from 2021-09-08; data sets included are listed in Table 1). These are supplemented by a series of published and yet unpublished dates from regional studies in western central Europe^76–78^. In order to avoid duplicates, lab numbers were filtered using regular expressions, and data with the same c14age, the same standard deviation, and the same geographical decimal coordinates (rounded at the 3rd digit, $\approx 100\,\textrm{m}$) were eliminated. Large parts of the database have already undergone filtering processes in the course of the original publications^79–82^. We take each date as a signal for a human activity, irrespective of its affiliation to the corresponding archaeological context. We therefore include dates which might reflect activities earlier or later than the associated features, but nevertheless signal human activity. We do not consider the inclusion of dates of possibly non-anthropogenic origin (e.g., incinerated roots, forest fires) as problematic.

The resulting dataset includes a total of 48,129 dates. We used a subset of 21,534 of these dates with mean calibrated age between 5000 and 9000 calBP, corresponding to the time period of our analysis. We compute summed probability distributions (SPDs) following the methodology and best practices of Crema and Bevan^83^; specifically, we used unnormalized probability distributions after calibration, and a binning scheme based on the sites with a temporal resolution of $h = 100$ years, thus reducing potential bias from different level of artifacts recovered and dated among different sites^3,4^.

**Table 1:** Sources of radiocarbon dates used in this study. Data set names are those defined in the c14bazAAR package^75^.

Data set	Date version	References
Agrichange	2021-05-21	^84^
AIDA	2021-09-08	^80^
BDA	2020-03-29	^85^
Calpal	2020-08-20	^86^
Context	2006-09-26	http://context-database.uni-koeln.de/
Eubar	2017-10-02	^87^, https://telearchaeology.org/EUBAR/
Euroevol	2015-07-09	^79^
Irdd	2018-08-13	^88^
Katsianis	2020-08-20	^89^
Medafricarbon	2020-03-20	^90^
NERD	2021-09-08	^82^
Pacea	2020-01-22	^91^
Palmisano	2017-09-23	^92^
Radon	Before 2021-09-08	^93^
Radonb	Before 2021-09-08	http://radon-b.ufg.uni-kiel.de

**Figure 6:**
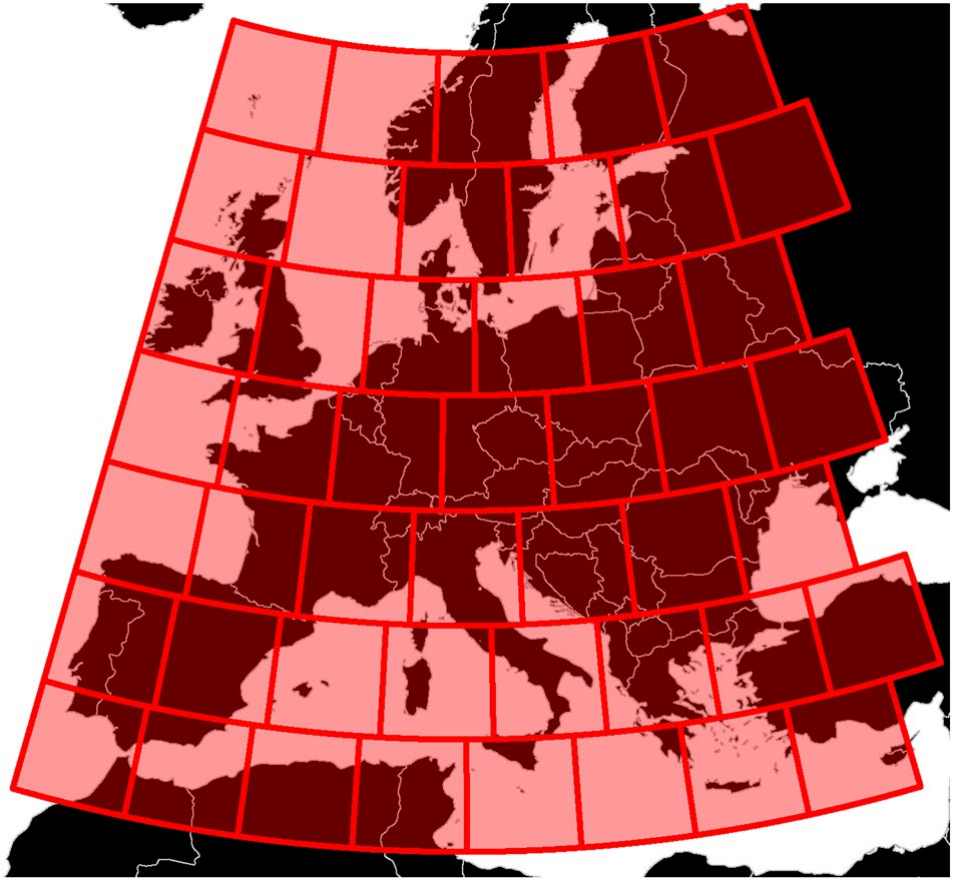
An example tiling of the study area, rendered using an Albers equal-area projection. Note that not all tiles contain valid data; in any grid position, only tiles with at least 500 radiocarbon dates were included in further analysis. Figure was generated with R (version 4.1.2, available at https://www.r-project.org/), using the ggplot2 package (version 3.3.5, available at https://ggplot2.tidyverse.org/). Base map data, including modern country borders was obtained using the rworldmap package (version 1.3-6, available at http://cran.r-project.org/web/packages/rworldmap) and is based on public domain data from the Natural Earth project, available at https://www.naturalearthdata.com/.

We aggregated the SPDs (note that simulation results were treated analogously) to form regional time series using uniform rectangular regions of a fixed linear size, i.e., 500 km (Fig. 6). The rectangles are tiled in a grid covering Europe starting from its southwestern corner. The tiling is carried out in an equirectangular projection, with the tiles’ sides being parallel to circles of latitude and meridians. At each latitude, regions are scaled along their longitudinal axis to retain approximately the same areal size. To improve robustness of the analysis, we performed aggregation over repeated samplings by shifting the starting point of the tiling scheme in 100 km strides, yielding, a total of 25 possible tilings.

Our approach is similar to previous studies^3–5^. However, instead of using predefined geographic regions, we use a regular tiling of different scales and grid alignments.

After calculating regional SPDs, we fitted a logistic growth model for each that represents growth with no boom and bust patterns. To account for the effects of sampling and calibration, we follow the methodology of Shennan et al.^3^ and Downey et al.^12^ to generate synthetic sets of radiocarbon dates proportionally to the fitted model. For each region, we generated 1000 sets of synthetic dates, each set containing the same number of dates as the original dataset in that region. For each synthetic date, we performed an “uncalibration”, i.e., assigned a 14C age to it based on the calibration curve, along with an error term, sampled from the measurement errors among the original dataset. Using the set of uncalibrated dates, we repeated the calibration and aggregation procedure for each synthetic dataset, producing a synthetic SPD. We used the mean value of these synthetic SPDs to detrend the SPD of the original dataset^3,12^, after confirming that the SPD of our dataset has statistically significant deviations from the set of synthetic datasets (see Fig. S8 in the *SI Appendix* for a comparison of synthetic SPDs to the 14C dataset in one grid position; also, Fig. S7 for a characterization of the deviations among all grid positions)^3,12,83^.

Finally, we calculated temporal autocorrelation functions (ACFs) for each detrended regional time series to characterize any cyclic behavior. For each individual ACF, we identify the location of the first minimum, and compile a frequency distribution over all regions. For perfectly periodic data, the first maximum can be interpreted as the typical cycle length of the time series, while the first minimum corresponds to the typical half-cycle length. For more complex time series this does not necessarily hold but the half-cycle length can be a more reliable indicator of the typical time scales of underlying processes. For this reason, our primary statistic for comparing the periodic components of the dynamics in the data and in the model is the location of the first ACF minimum.

To characterize the average amplitude of both empirical and model-predicted dynamics we calculated the coefficient of variation (the standard deviation of the detrended time series divided by the mean of the trend). This measure characterizes the relative magnitude of variance while taking into account the non-stationarity due to long-term trends.

Figure 2a shows calculated SPDs for a sample subset of regional tiles (data for all regions are in *SI Appendix* Fig. S8). In the absence of second order boom-bust dynamics, we expect S-shaped trajectories arising from regional logistic growth, with random (“white noise”) fluctuations superimposed on S-curves. The most frequent pattern, however, is that of recurrent booms and busts that follow an initial growth after the spread of farming into the region. When comparing the SPD time series with the fitted trend (*SI Appendix* Fig. S8), booms and busts appear to recur on a time scale of c. 1.000 years, although with much variation around this mean value. This is apparent in the ACFs of the detrended time series (Fig. 2b). After aggregation of ACF minima in repeated tiling, their distribution has a peak at 500 years, and most common half-lengths cluster within 300–1000 year interval (Fig. 2c). The distribution of CVs clusters around a peak between 0.25 and 0.5 (Fig. 2d). Distributions in Fig. 2 (panels c and d) in the main text presents results aggregated over the 25 tiling positions that we used in this study. Disaggregated results, i.e., individual distribution of ACF minima and CV values in each tiling position are shown Fig. S6 in the *SI Appendix*. In all grid positions, we see a similar pattern in these distributions.

### Model operation

We use a spatially-explicit agent-based model with a spatiotemporal focus on mid-Holocene Europe. Main model parameters are summarized in Table 2; Fig. 3 shows a representation of the simulation space. We use an equal-area hexagon grid created with the DGGRID software^50,51^. Hexagons have an area of $\approx 96\,\textrm{km}^2$ (grid type: ISEA3H, resolution level: 12). The simulation area includes 33,473 cells in total, after accounting for coastlines and removing cells with low agricultural productivity (i.e., a carrying capacity of less than 50 people).

Each hexagonal cell is either empty, occupied by farmers or, optionally, occupied by “aggressors” engaging in regular violent conflicts. Forager groups are not included in the current simulation; it is assumed that foragers have significantly lower population densities than farmers and thus the interactions between these groups are minimal with farmers outcompeting foragers^40,94^. Archaeological evidence for direct interaction among foragers and farmers in Europe exists^8^ and conflicts are assumed to have happened^40,45,47^, while archaeogenetic evidence currently shows that biological interaction between populations occurred largely during later phases^95,96^.

Each cell in the simulation area is assumed to be an independent political unit (i.e., one “village”); the formation of larger-scale entities, such as alliances or vassal relationships are not considered. In the current work, we consider agricultural villages to have a typical maximal size of about 150 persons (Dunbar’s number; cf.^97^), consistent as well with estimates for Neolithic villages^7,11,28^. Size of the cells was chosen to make this number plausible, assuming an average population density of 1–2 persons per $\hbox {km}^2$ for Neolithic farmers^14,98^. See the *SI Appendix* for a more detailed description of the process of creating the cell grid and estimating local carrying capacities.

Simulations begin with an initial population located in Anatolia in the year of 7000 BCE. To account for the different nature of models based on whether violent conflicts are included, we used a distinct set of initial conditions.

- In the case of no conflicts, the simulation begins with an initial population of 100,000 people, distributed uniformly within a group of cells in a starting region. The large number of people is necessary to avoid a longer “warm-up” phase where logistic growth occurs slowly before migration begins. In this case, choice of the initial population size does not affect the results, only the length of the initial simulation phase.
- In the case where conflicts are allowed, the simulation begins with an initial population of 5000 people distributed uniformly within a random subgroup of cells in a starting region. The lower starting population helps ensure that cells’ population are not likely to immediately fission; the random subset of cells helps ensure sufficient empty space between occupied cells to avoid early eruption of high levels of violence.

Population dynamics within each cell follows a logistic growth up to a carrying capacity. We set the average carrying capacity of cells to 150 persons, while we allow variation both in space and time according to estimated suitability to agriculture and climate. Relative variation in space was estimated from a dataset of “agro-climatic attainable yield” applied to low-intensity, rain-fed cereal agriculture from FAO GAEZ^52,53^.

Temporal variation in carrying capacities is based on a model of agriculture yield emulator of Franke et al.^54^ that takes the set of main climate parameters as its input. We use the reconstruction of past climate data of Armstrong et al.^55^ as model inputs; we take the output of this procedure as a measure of relative variation of agricultural productivity (and thus carrying capacity) in time independently for each cell (the procedure is explained in more detail in the *SI Appendix*). To account for uncertainties in estimating the real effect of climate under the conditions of Mid-Holocene agriculture, we introduce an additional scaling factor, *s*, that adjusts the magnitude of variability in cell productivity. Note that $s = 1$ is the value that corresponds to the magnitude of climatic effects on carrying capacity estimated by feeding past climate data into the crop emulator. To explore the effects of assuming stronger climate effects, we ran models with $s = 1$, 2, 3, and 4 (see the *SI*). In the main article we report results for $s = 2$; in other words, our approach is conservative with respect to rejecting the climate hypothesis. The time-varying carrying capacity of each cell is thus modeled according to the following formula (constrained to nonnegative values):

1 $$\begin{aligned} {K}_{i} (t) = 150 \frac{{G}_{i}}{{\left\langle {G}_{j} \right\rangle }} \left( 1 + s \left( \frac{{A}_{i} (t)}{{\left\langle {A}_{i} (t') \right\rangle }} - 1 \right) \right) \end{aligned}$$

Here $G_i$ is the (absolute) attainable productivity estimated from the FAO GAEZ dataset (obtained as multiplying the base yield with the land area of the cell, excluding slopes over 30%); $A_i(t)$ is the time-varying relative yield in cell *i*, based on the yield emulator model of Franke et al.^54^ evaluated using the climate data from Armstrong et al.^55^. Note that the average in the denominator of the first term is calculated over all cells in the simulation area, while the average in the denominator with $A_i$ is calculated for all years in the study period, but only for cell *i*. This way, the second term that corresponds to scaling by climate variations has a mean of one, i.e., it does not affect average carrying capacities, but only adds temporal variation. Carrying capacities are limited to non-negative numbers; very large downward deviations can result in zero capacity, making a cell temporarily uninhabitable. This does not happen when $s = 1$, but is possible for larger values. Setting $s = 0$ results in cell carrying capacities being constant in time, omitting any effect of climate variation.

In the model group migration and population spread depends on split-off events. As the population of a cell grows, the probability of a split-off event increases according the following linear relationship:

2 $$\begin{aligned} P_i^s = \left\{ \begin{array}{ll} 0 &{} \quad \text {if } N_i < N_0 \\ \min \left( 1, \alpha (N_i - N_0 ) \right) &{} \quad \text {otherwise} \end{array} \right. \end{aligned}$$

We assume that there is a lower limit ($N_0$), below which split-off events never occur. Above this, the probability of such events is a linear function of the population, reaching 1 if $N_i = N_0 + 1/\alpha$. We used $N_0 = 100$ and $\alpha = 1 / 400$, implying that no split-off events occur for groups below 100 people, and for a group size of 150 people a split-off event would occur on average once every 8 years, while a split-off would happen with certainty for a group of 500 people (a size that is not reached in our simulations due to the limited carrying capacity of cells). Note that our formulation is not dependent on any specific interpretation of the underlying processes (e.g. “approaching Dunbar’s number”), only on the generic notion that larger groups have higher probabilities of splitting. This is also true regarding the functional form used in Eq. (2); in the *SI Appendix* (Fig. S18) we show that using an alternate functional form where the probability of fission depends on a logistic function of population^61^ also gives similar results in terms of the time scale and amplitude of boom-bust patterns.

If a split-off event happens, the size of the subgroup is randomly chosen from a Poisson distribution with expected value of half the population. A target cell is selected according to the following probability distribution:

3 $$\begin{aligned} {P}_{ij} = \frac{{f}_{ij}}{\sum _{k \ne i}{{f}_{ik}}} \end{aligned}$$

where the $f_{ij}$ factors represent the attractiveness of each possible target cell and are calculated according to the following formula:

4 $$\begin{aligned} {f}_{ij} = {K}_{j} {e}^{{- {d}_{ij}} / {G}} {E}_{j} \end{aligned}$$

Here $d_{ij}$ is the scaled distance between cells *i* and *j*. *G* determines the typical spatial scale of migrations. $K_j$ is the base carrying capacity of cell *j*; thus cells with higher carrying capacities are preferred. $E_j$ expresses a preference for empty target cells:

5 $$\begin{aligned} E_j = \left\{ \begin{array}{ll} 1 &{} \quad \text {if cell } j \text { is empty} \\ p_E &{} \quad \text {if cell } j \text { is occupied} \end{array} \right. \end{aligned}$$

The parameter $p_E$ quantifies willingness to attempt to enter an occupied cell, which triggers conflict with its inhabitants. In the pure climate model, this parameter is set to 0, thus, no conflict occurs. In this case, if no empty cells are available within 10*G* distance, the migration attempt is canceled.

The distance between two neighboring cells is computed as the great-circle distance between their center points; for non-neighbor cells, the distance is computed along the shortest possible path. We only allow travel through land cells, coastal cells and two sea links that we added across the Aegean Sea and the Strait of Otranto between the Balkans and the Italian peninsula. We also treat sea travel along the coast of the Mediterranean Sea specially^99^: distances between coastal cells and along the two sea routes are scaled by factor of 0.1 permitting efficient long-distance travel.

In the warfare model version, selecting an already occupied cell as migration target gives rise to conflict, which will eventually result in the defeat and elimination of either the previous occupants of the cell (defenders), or of the attackers. In the main model variant, the winner of such a confrontation has a chance of becoming militarized, converting to “aggressors”. Model parameter $p_S$ gives the probability of attackers winning, and $p_C$ determines the probability of the winner of a conflict converting to an aggressor. Finally, parameter $p_R$ gives the yearly rate at which aggressors convert back to peaceful farmers. Note that if $p_C = 0$, then aggressors are never created, and the level of conflict is directly determined by the density of occupied cells and $p_E$. In this case, our model is first-order, i.e., there is no memory in the process determining the level of conflict. For $p_C > 0$ however, the conditions of warfare are persistent, as conflict will continue after an initial trigger and only decrease with the rate determined by $p_R$.

While we assume that aggressors maintain a more violent attitude in general, we do not model the details of these dynamics. Instead, for each aggressor cell, we only focus on cases when a large-scale violent confrontation ensues with a nearby farmer cell that has the potential of causing extinction of one party to conflict and resulting in winners becoming aggressors themselves. We assume that such large-scale confrontations happen for each aggressor cell with probability $p_A$ in each year; this means that such events happen every $1/p_A$ years on average. However, this does not mean that no other confrontations happen in the meantime as there is abundant ethnographic evidence that warfare between small-scale societies mostly consists of events with a small number of casualties with only some of them escalating to large-scale ambushes and massacres^63,64^. In our model, we abstract away the specific sequence of events, and focus only on outcomes where a settlement is either eradicated, or becomes militarized as it is exposed to increasing amounts of violence.

Given these considerations, for each aggressor cell, if a large-scale confrontation happens, we select its target in a probabilistic manner, similarly to migrating groups:

6 $$\begin{aligned} {p}_{ij}^{B}= & {} \frac{{f}_{ij}^{B}}{\sum _{k \ne i}{{f}{ik}^{B}}} \end{aligned}$$

7 $$\begin{aligned} f_{ij}^B= & {} \left\{ \begin{array}{ll} F_j N_j e^{-d_{ij} / B} &{} \quad \text {if } d_{ij} \le B_\text {max} \\ 0 &{} \quad \text {otherwise} \end{array} \right. \end{aligned}$$

Here $F_j$ is an indicator with the value of 1 only if cell *j* is occupied by farmers, and zero otherwise; this ensures that attacks only target farmers. $N_j$ is current population of the cell, reflecting a preference for cells providing more available production. Note the the inclusion of this term is not an essential feature of the model; as we show in the *SI Appendix* (Fig. S18), we gain similar results even if aggressors do not take into account the population of cells when selecting targets. *B* establishes the characteristic distance for attacks; *B* is typically small reflecting a preference for cells that are close, but in any case, such attacks are limited to explicit cut-off distance, $B_\text {max}$. If no attack targets are found within this maximum distance, aggressors revert back to farmers.

Similarly to conflicts triggered by split-off events, we decide the “winner” according to probability $p_S$. If this is the aggressor cell, the target goes extinct (i.e., the cell becomes empty). On the other hand, if aggressors lose, they are not affected, but the successful defenders themselves convert to aggressors with probability $p_C$.

In the main conflict variant aggressors are stationary and aggression spreads by possible militarization of attacked cells. In this case, we used parameter values of $p_S = 0.5$ and $p_C = 1$, along with a rate of $p_R = 0.01$ of aggressors converting back to farmers. Alternatively, aggressors can roam, i.e., after a successful attack, they move to the cell of their target, abandoning their original location. In this case, we used parameter values of $p_S = 1$, $p_C = 1$ and $p_R = 0.01$. In this manner the number of aggressors does not grow but they spread conflict by moving around in the landscape. In a further variant, aggressors only move to a new cell after the initial confrontation, i.e. as the result of group fission. Results for these variants are shown in the *SI Appendix*.

**Table 2:** Summary of main model parameters and their typical values.

Parameter	Description	Typical value
*r*	Base population growth rate	0.0135
$\alpha$	Scaling factor for group fission probabilities	1/400
$N_0$	Minimum population for group fission	100
*G*	Characteristic distance of migrations	$40\,\textrm{km}$
$p_E$	Probability of targeting an occupied cell	1
*B*	Characteristic distance of attacks	$8\,\textrm{km}$
$B_\text {max}$	Maximum attack distance	$80\,\textrm{km}$
$p_A$	Probability of major conflict per year	1/10 years
$p_S$	Attack success probability	0.5
$p_C$	Probability for conversion to aggressors (upon being exposed to conflict)	1
$p_R$	Aggressors’ probability of spontaneous conversion back to peaceful farmers	0.01
*s*	Relative magnitude of agricultural productivity variation due to climate	2

## Supplementary Information

Supplementary Information 1.

Supplementary Information 2.

Supplementary Information 3.

Supplementary Information 4.

Supplementary Information 5.

## Data Availability

Simulations were carried out by software written by the authors. Source code is available in the respective public repositories^100,101^. Several publicly available datasets were used in creating the simulation space. Scripts and detailed instructions to obtain these are available along with the simulation source code^100,101^. The radiocarbon datasets used in this study were obtained from publicly available sources using the c14bazAAR R package^75^ (version from 2021-09-08; data sets included are listed in Table 1) along with yet unpublished dates from regional studies in western central Europe^76–78^. Unpublished radiocarbon dates used in this study are available from Dániel Kondor on request.
