# Supplementary material for: Explaining population booms and busts in Mid-Holocene Europe
Source: Sci Rep. 2023 Jun 8;13:9310. doi: 10.1038/s41598-023-35920-z (PMC10250413; doi:10.1038/s41598-023-35920-z)
Supplement: Supplementary file 1 — Supplementary Information 1. [file 41598_2023_35920_MOESM1_ESM.pdf]

Explaining population booms and busts in Neolithic Europe

Supplementary Material

Dániel Kondor, James S. Bennett, Detlef Gronenborn, Nicolas Antunes, Daniel Hoyer, Peter Turchin

May 8, 2023

Chapter 1

Supplementary text

1.1 Agriculture and climate data

1.1.1 GAEZ yield and terrain data

We use the FAO GAEZ dataset [1, 2], which contains a large array of variables related to land, terrain, and agricultural productivity, to initialize our estimates of Neolithic agricultural yield . Data is typically provided in a rectangular grid with 5 arcminutes (1/12 degree) resolution. We use the "Agro-climatic attainable yield" variable (under the group "Suitability and Potential Yield", subgroup "Agro-climatic yield") for "spring wheat" and "winter wheat" crop types. For our base yield estimates (in tonnes per hectare), we used the larger of the spring or winter wheat yield values for low-intensity, rain-fed agriculture ignoring fallowing practices, which we assume corresponds to presumed technologies available in the Neolithic. We calculate the weighted average of the yield values of any overlapping rectangular grid regions as the base yield for each of our hexagon cells. The base agricultural productivity of the cell is then calculated by multiplying the base yield with the area of the cell, excluding the share covered with water or having a slope greater than 30% (as given by the GAEZ database).

The base productivity estimates are rescaled to provide an average carrying capacity of 150 people per cell. We mark cells with mean carrying capacities below 50 people as unsuitable for agriculture. The standard deviation of carrying capacities among remaining cells is 35.7 persons.

We find that the typical (unscaled) agricultural yields in the GAEZ data would imply significantly higher population densities than present in the Neolithic period. The average yield value in the dataset over the simulation area is 2.41 t/ha. If all available land were used (after accounting for land covered by water and land with high slope), this would imply an average agricultural production capacity of 20,286 tonnes of wheat per cell, or an average carrying capacity of over 100,000 people per cell (assuming an average yearly consumption of 0.2 tonnes per person).

Clearly, land is not used to such an extreme extent in even modern times, let alone by the first Neolithic farmers. While there are many challenges to estimating actual crop yields in the Neolithic period, multiple studies suggest that values around 1 t/ha are reasonable to expect on land that was actively farmed [3, 4, 5, 6] with some studies suggesting that higher values might have been possible under good conditions [7, 8]. This way, the main difference in population densities comes from limitations in land-use patterns and socio-cultural constraints. There is evidence that land used for farming was supplemented by additional land used for either pasture or fallow [7, 8, 9, 5]. A conservative estimate gives at least 2.35 ha core agrarian land used per person (of which only 0.3 ha is used for farming) and at least an additional 8.9 ha of land used for "low-intensity activities" [9, 5]. Such an estimate limits the maximum population of our hexagon cells to 4,084 persons if only considering core land-use, or 853 persons if space for additional low-instensity activities is included. Beside limitations stemming from technological constraints, settlement patterns on larger scales were further affected by cultural practices resulting in large empty areas among densely-occupied territories [10, 5]. This way, overall population densities in Neolithic Europe are estimated between $0.5 - 2$ persons per km^2 [11, 12, 5], consistent with our target of 150 persons per hexagon cell.

1.1.2 Climate-based variation

For each cell, a time-dependent measure of relative agricultural productivity is calculated based an agriculture yield emulator model of Franke et al. [13] which we, in turn, drive using detailed (yearly) past climate data of Armstrong et al. [14] over a subset of the time period of their dataset (10,000 BP to 2,500 BP). The relative productivity for each cell is normalized by average productivity for the entire period and these temporal z-scores are applied as a multiplicative factor to arrive at yearly relative yield estimates.

Yield emulator

Franke et al. [13] provide fitted polynomial models to estimate agricultural yield potential as a function of climatic conditions. They built their models by running established agricultural productivity emulator models for a set of assumed present and

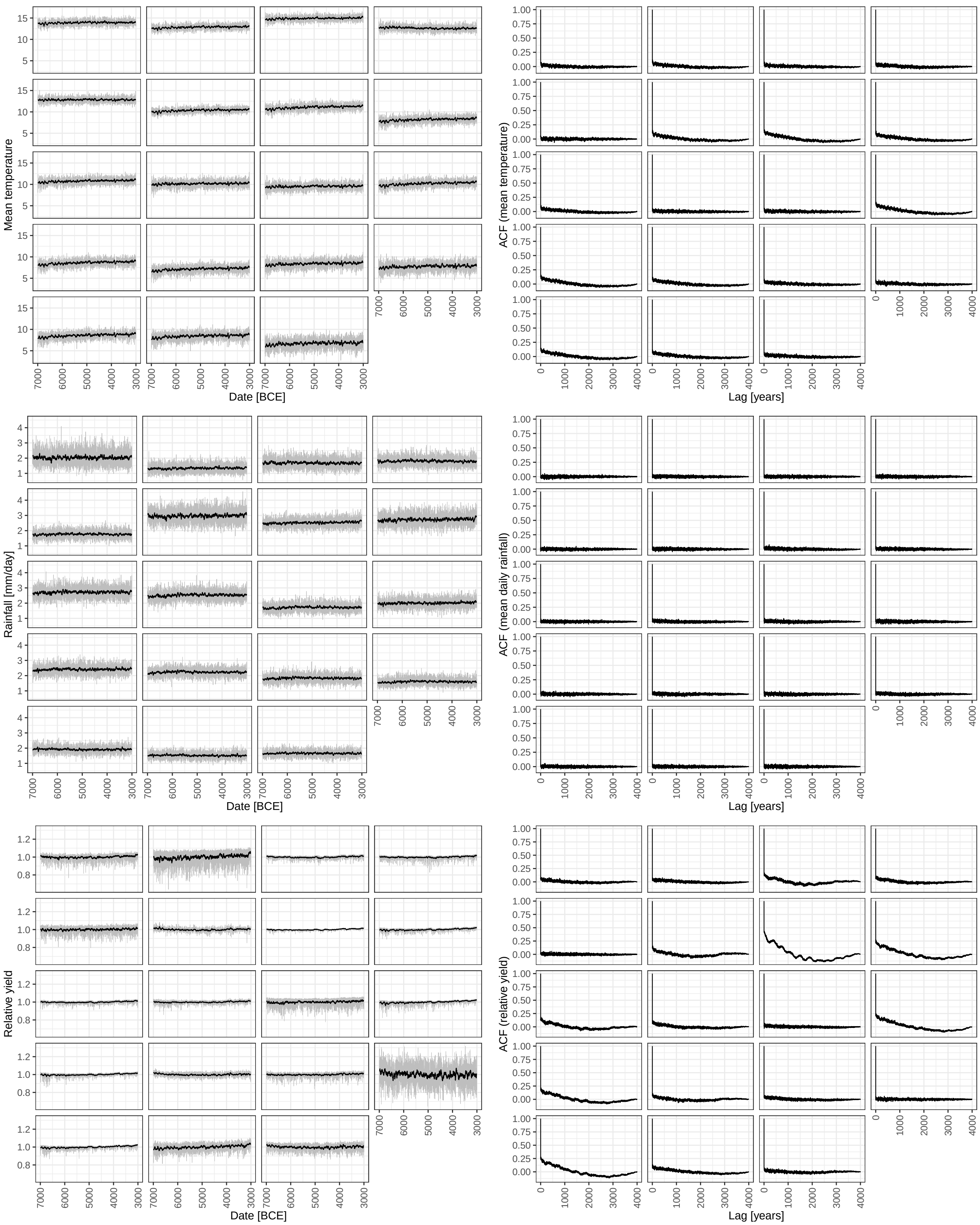

Figure S1: Typical regional variations in mean temperatures (top row), rainfall (middle row) and estimated agricultural productivity (bottom row). Left panels display time series of regional mean temperature; the black lines show 30 year moving averages. Right panels show the autocorrelation of the same. We see that the patterns are dominated by short-term variations along with some gradual long-term trends; no periodic components are present.

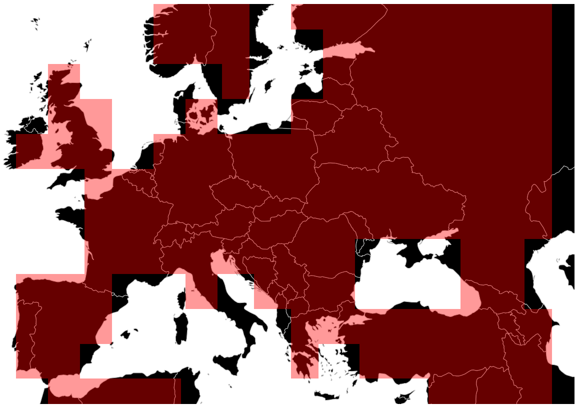

Figure S2: Areas covered by the climate data used in this study. Figure was generated with `R` (version 4.1.2, available at `https://www.r-project.org/`), using the `ggplot2` package (version 3.3.5, available at `https://ggplot2.tidyverse.org/`). Base map data, including modern country borders was obtained using the `rworldmap` package (version 1.3-6, available at `http://cran.r-project.org/web/packages/rworldmap`) and is based on public domain data from the Natural Earth project, available at `https://www.naturalearthdata.com/`.

future climate variable combinations, and then fit a third-order polynomial to reproduce those detailed estimates. These polynomial models thus rapidly estimate the results from the otherwise prohibitably expensive original emulators.

Polynomial models are provided for 9 different agricultural emulators (hence 9 parameter sets) with each emulator/parameter set targeting five crop types. In this work we used the polynomial model that was fitted for the LPJmL emulator [15] for spring and winter wheat. Our analysis could be easily extended to include models for additional emulators and crop types.

For each estimator and crop type, Franke et al. fitted a polynomial model independently in each one of thousands of 0.5 degree grid cells covering the land areas of the Earth. These (several million) fitted parameter values can be used to estimate agricultural yields world-wide given a wide possible range of climate parameters. These parameters are provided in a public repository for download [16].

Each polynomial model has four input variables: atmospheric carbon dioxide concentration (C), temperature (T), rainfall (W) and nitrogen application (N). Values for C, T and W are assumed to be averages during the growing season; within-year variation is assumed to follow the typical patterns observed in the 31-year baseline period between 1980 and 2010. Values for C are given in units of part per million (ppm), while the T and W input variables are interpreted as absolute and relative difference from the 31-year average data in the baseline period, according to the AgMERRA dataset [17, 18]. Setting each input value to their default value (i.e. $C = 360\,\text{ppm}$, $T = 0$, $W = 1$, and $N = 200\,\text{kgha}^{-1}$) allows the calculation of the agricultural productivity in a cell during the baseline period.

Climate data

Climate data for the baseline time period (1980-2010) is provided as part of the AgMERRA dataset in a rectangular grid, at a resolution of 0.25 degrees as daily values [17, 18]. For the purpose of the current study, we calculated average values of temperature and rainfall over the whole baseline time period in a 0.5 degree resolution grid consistent with the yield dataset. A more precise estimate would consider the growing period for each crop type (as different magnitudes of change can occur at different times of the year).

Armstrong et al. [14] provide historical climate data for the northern hemisphere on a 0.5 degree resolution grid for the past 60,000 years. The data includes temperature and rainfall data at a temporal resolution of one month, allowing a potential extension for month-by-month comparison with the baseline climate data. In the current study, we calculate yearly averages for the study period between 8,050 BCE to 550 BCE, i.e. 2,500 to 10,000 BP. While the main focus of our simulations is the time period between 7,000 to 3,000 BCE, we generated estimates of agricultural productivity in a longer time period to be able to vary the simulation start date and to avoid edge effects at the end of the simulation when employing a sampling procedure to generate simulated SPDs.

The dataset covers most of the land area of Europe as shown in Fig. S2. In areas without climate data, we assumed a constant value of agricultural productivity, as estimated from the GAEZ yield dataset. These areas were included in the simulation, but they were excluded from the analysis of population time series so as not to add bias to the results.

We a reconstructed timeseries of atmospheric CO2 concentrations published by the EPA [19], performing a linear interpolation to get a yearly time series. Finally, we set the nitrogen application level to zero; additional insight about the impact of fertilizer use (e.g. manuring) could be incorporated in our model by varying this level over time.

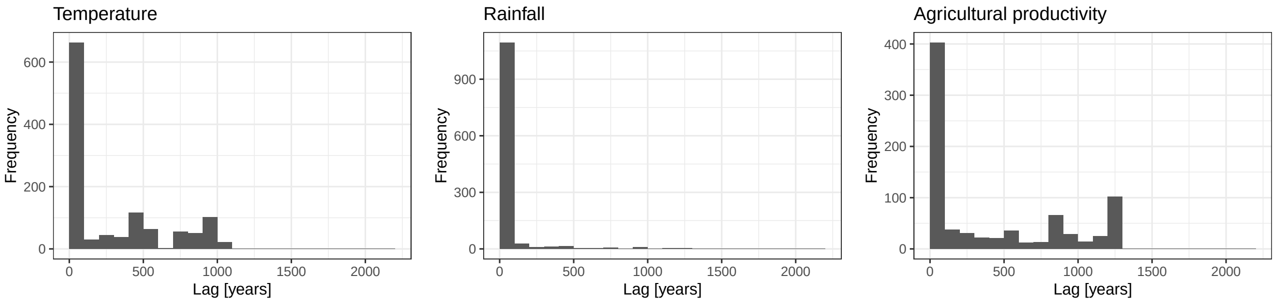

Figure S3: Frequency distribution of the location of the first minimum in the ACF of regional time series of temperature, rainfall and estimated agricultural productivity.

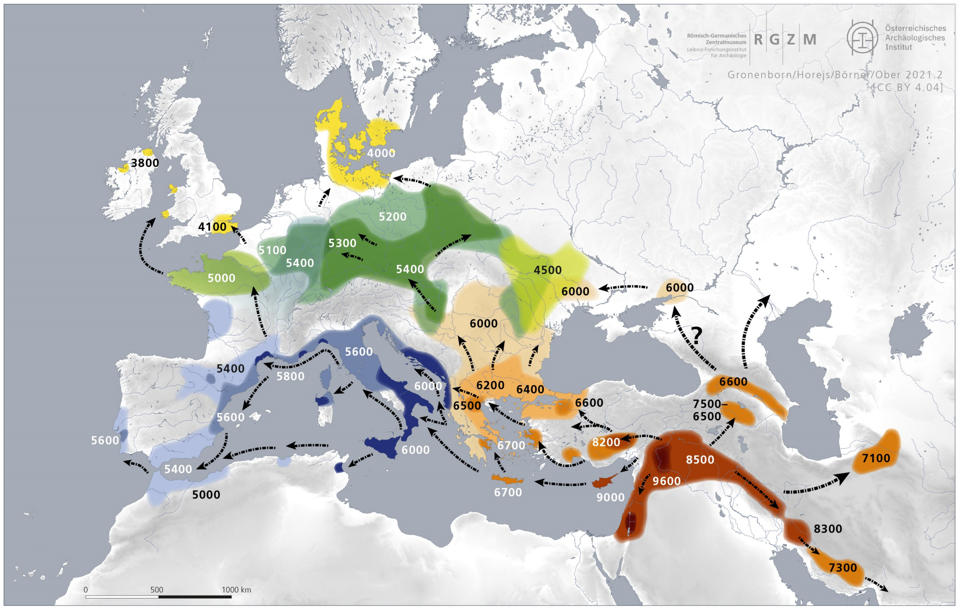

Figure S4: Archaeologically established dates of first settlement of regions by farmers in Europe. Figure reproduced from [20], under the CC BY 4.0 license.

Temporal patterns in climate and agricultural productivity

To better understand potential direct effect of temporal patterns in climate on population, we analyzed regional time series of climate variables (temperature and rainfall) and estimated agricultural productivity variations in a similar fashion to our simulated population data. Figure S1 shows examples of regional time series (using the same aggregation scheme as for the radiocarbon dates and simulation results in our study).

We display aggregate distribution of ACF minimum locations in Fig. S3 for climate variables and estimated agricultural productivity. In accordance with the patters seen in Fig. S1, the large majority of ACF minimums is detected for very short times (i.e. below 100 years), indicating that there are no strong cyclic patterns present.

1.2 Spread of farming in Europe

As an additional validation of our model, we compared the simulation results to archaeologically established dates of settlement by farmers throughout Europe. We used an estimation of regional settlement patterns from a previous study [20], displayed in Fig. S4. This dataset contains a set of 33 regions, with 19 unique dates of settlement. For each region, we identified the set of cells in our simulation space contained in it, and identified the first time (in simulation years) when a given p share of the cells in each region was settled (with $p = 0.1$ for the model variant without conflict, and $p = 0.02$ for the variant including conflict to account for the lower total share of cells settled).

We show these times along with a fitted linear relationship in Fig. S5 for model variants both excluding and including violent conflict (in the latter case, we used model parameters $p_E = 0.1$, $p_A = 0.1$). We varied main parameters G (i.e. the characteristic distance of migrations), s (scale of climate-based variability of agriculture) and the simulation start date. We see that a good linear correspondence can be established, with the slope varying according to the G and s parameters. The approximately linear relationship shows that settlement patterns are reproduced reasonably well in the simulation, while a

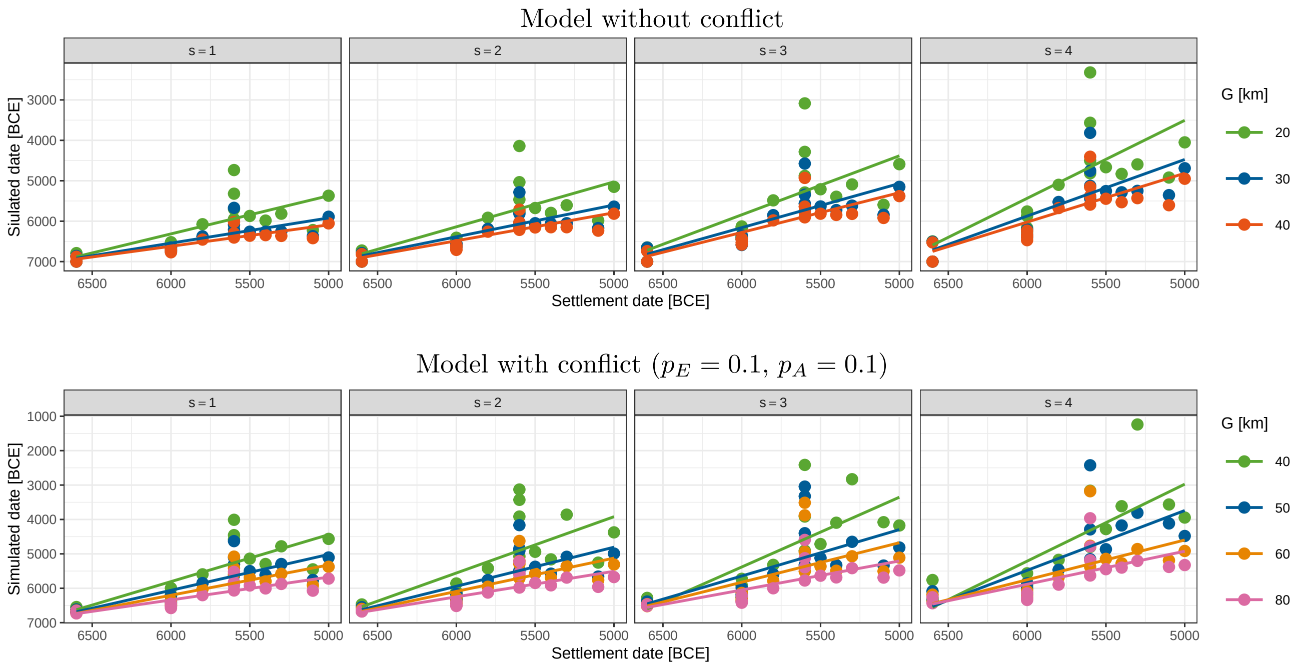

Figure S5: Simulated time of settlement in archaeologically relevant regions as a function of archaeologically reconstructed settlement dates. Different colored points correspond to different migration characteristic distances (G). Solid lines show fitted linear relationships. Larger slopes correspond to slower speed of spread. Top panels show results for the model variant without conflict (i.e. $p_E = 0$), bottom panels show results for the model variant including violent conflict.

	slope			
G [km]	$s = 1$	$s = 2$	$s = 3$	$s = 4$
20	0.943	1.113	1.459	1.929
30	0.622	0.788	1.093	1.400
40	0.531	0.693	0.982	1.209

Table S1: Fitted coefficients of a linear relationship between archaeologically estimated and simulated settlement dates of a set of regions in Europe (model variant without conflict). A slope of 1 would mean a perfect correspondence; slopes below 1 indicate a faster, slopes above 1 indicate a slower spread of farmers in the simulation than expected. Notably, for higher values of s (presumed variation of agricultural productivity due to climate) the spread of farming slows down significantly. This can be compensated by using larger values of G.

	slope			
G [km]	$s = 1$	$s = 2$	$s = 3$	$s = 4$
40	1.364	1.636	2.0282	2.2370
50	1.043	1.136	1.3480	1.7392
60	0.880	0.968	1.1479	1.1560
80	0.664	0.742	0.8426	0.9590

Table S2: Fitted coefficients of a linear relationship between archaeologically estimated and simulated settlement dates of a set of regions in Europe (model variant with conflict, $p_E = 0.1$ and $p_A = 0.1$). A slope of 1 would mean a perfect correspondence; slopes below 1 indicate a faster, slopes above 1 indicate a slower spread of farmers in the simulation than expected. Notably, for higher values of s (presumed variation of agricultural productivity due to climate) the spread of farming slows down significantly. This can be compensated by using larger values of G.

slope close to 1 indicates a correspondence in the actual speed of spread. In Tables S1 and S2, we show the fitted slope of the linear relationship between simulated and archaeologically established settlement dates. Depending on the model variant and the value of s, different G parameters give the closest approximation of the true spread of farming in Europe. In the main analysis, we have used a simulation start date of 7,000 BCE, $s = 4$, and $G = 40\,\mathrm{km}$ for the model variant without conflict, and $G = 80\,\mathrm{km}$ for the model variant including conflict.

Chapter 2

Supplementary figures and videos

2.1 Video captions

Supplementary Video S1. Simulation results for the model variant without the conflict component for parameters $G = 40\,\mathrm{km}$, $s = 2$. Grid cells are colored according to their population; lighter colors indicate larger values.

Supplementary Video S2. Simulation results for the model variant without the conflict component for parameters $G = 40\,\mathrm{km}$, $s = 4$. Grid cells are colored according to their population; lighter colors indicate larger values.

Supplementary Video S3. Simulation results for the main model variant with the conflict component (stationary aggressors), for parameters $G = 80\,\mathrm{km}$, $s = 2$, $p_E = 1$ and $p_A = 0.05$. Grid cells are colored according to their population; lighter colors indicate larger values. Red cells indicate aggressors.

Supplementary Video S4. Simulation results for the main model variant with the conflict component (stationary aggressors), for parameters $G = 80\,\mathrm{km}$, $s = 2$, $p_E = 0.2$ and $p_A = 0.1$. Grid cells are colored according to their population; lighter colors indicate larger values. Red cells indicate aggressors.

2.2 Disaggregated results for 14C data

In Fig. S6, we show ACF and CV distributions for each of the grid tiling positions used in our study for the 14C dataset.

2.3 Regional results

In Figs. S8 and S9, we display typical regional population time series and their ACFs estimated from the 14C dataset in one tiling position. In Fig. S8, we display the computed SPD, the fitted logistic growth time series and the confidence interval obtained from generating 1,000 synthetic datasets based on the fitted time series. Fig. S9 shows the ACFs of the detrended SPD time series, i.e. of the time series obtained after subtracting the mean SPD of synthetic datasets from the SPD of the 14C dataset.

In Fig. S10-S13, we display regional population time series and ACFs for typical simulation results in one tiling position. Figs. S10 and S11 show population and ACF for the simulation variant without conflict (i.e. with $p_E = 0$), but including climate variation; figs. S12 and S11 show population and ACF for the simulation variant that includes both conflict and climate variation components.

2.4 Variation of main parameters

In Figs. S14-S17, we show results (in terms of the distribution of ACF minima and CV values) in the three main simulation variants while varying the values of the most important parameters (additional parameter variations are presented in the next section). All of these results are based on 100 repeated realizations of the simulation with the given parameter values and compiling aggregate histograms of the results among these.

In Fig. S14, we show these results for the simulation variant without conflict (i.e. $p_E = 0$), but including climate variations. In this case, we test variations in the s and G parameters.

In Figs. S15 and S16, we show ACF minima and CV distributions for the main simulation variant that includes both conflict and climate variation. In Fig. S15, we used $s = 2$ and $G = 80\,\mathrm{km}$ and varied the p_E and p_A parameters. We see that the mode mode and shape of distributions changes gradually with the parameters. In Fig. S16, we used $p_E = 1$ and $p_A = 1/20$

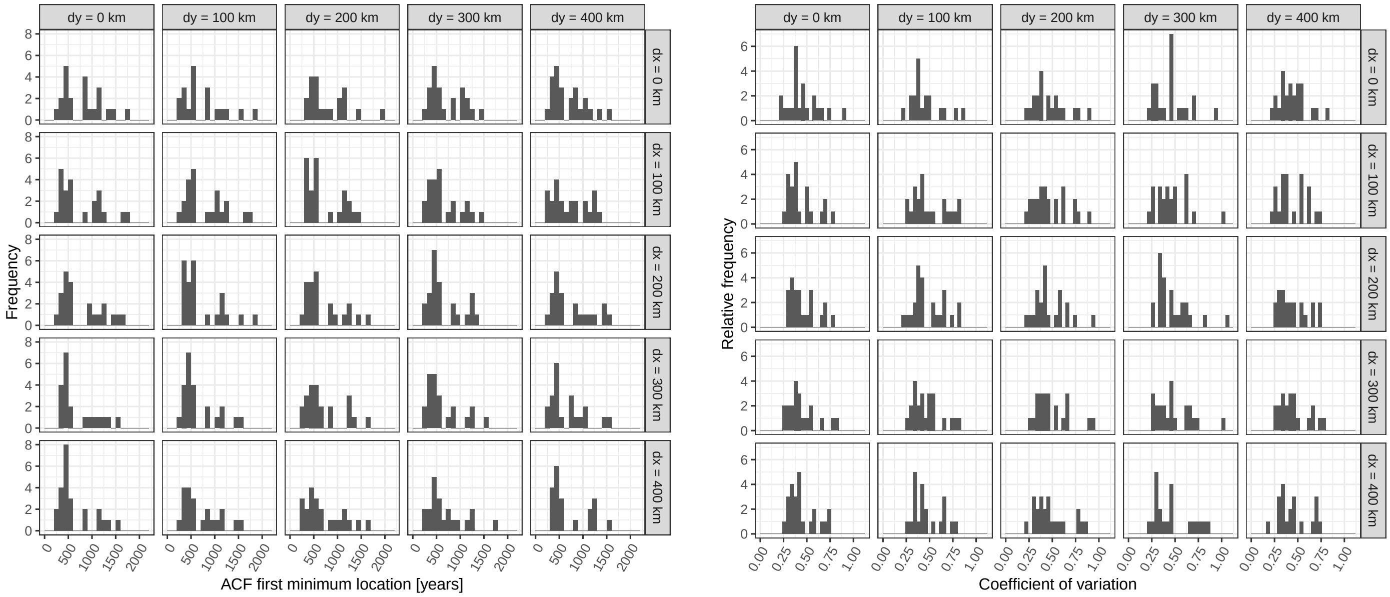

Figure S6: Frequency distribution of the location of the first minimum in the ACF (left) and CV of SPDs (right) for the 14C dataset. Each facet corresponds to one possible location of the tiling grid used for aggregation.

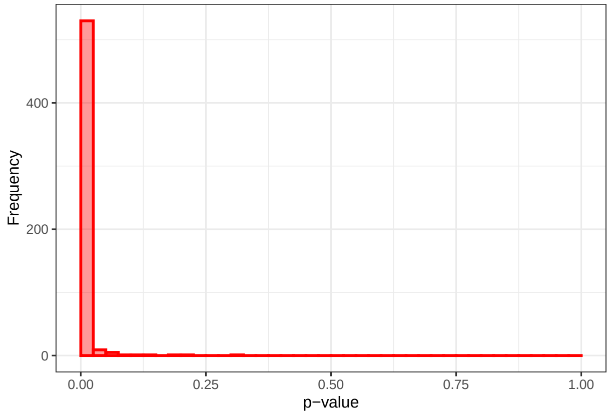

Figure S7: Frequency distribution of p-values obtained when testing the significance of temporal patterns in 14C data against synthetic datasets generated from a logistic null model [21, 22]. Distribution of values across all regions in all grid positions are displayed here ($N = 550$). Most p-values are small, indicating that in most cases, the deviation from the logistic growth null model is significant.

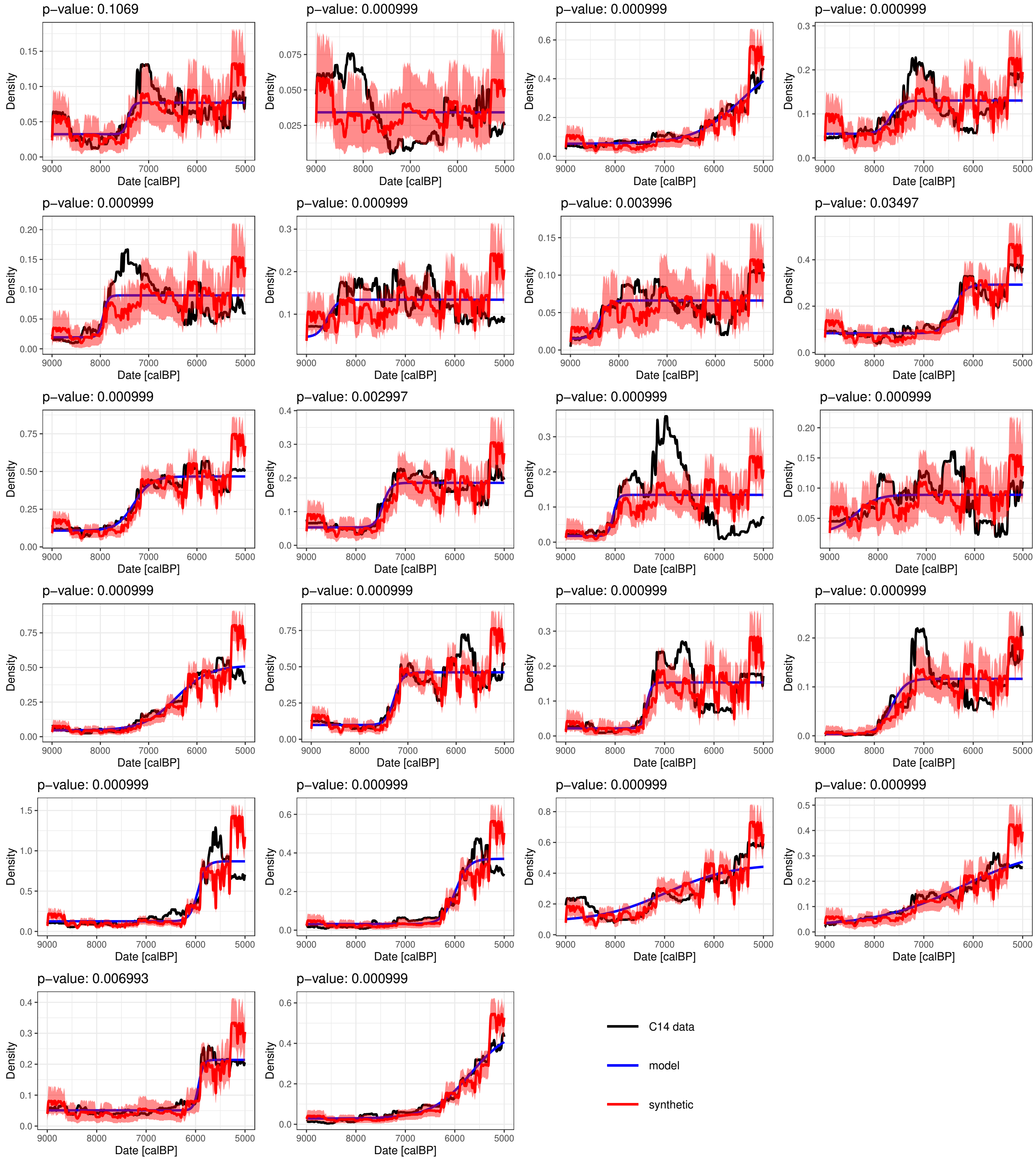

Figure S8: Regional SPDs computed from radiocarbon dates. Blue lines indicate the fitted logistic trend for SPDs, showing typical S-shaped patterns that describe well the overall trend in most cases. Red areas show the 95% confidence intervals resulting from 1,000 synthetic SPDs. The p-values are estimated as the share of synthetic SPDs with a higher total z-score outside of the 95% interval than the real data [21, 22]. We see boom and bust patterns in most regions, with typical timescales of 500-1,000 years.

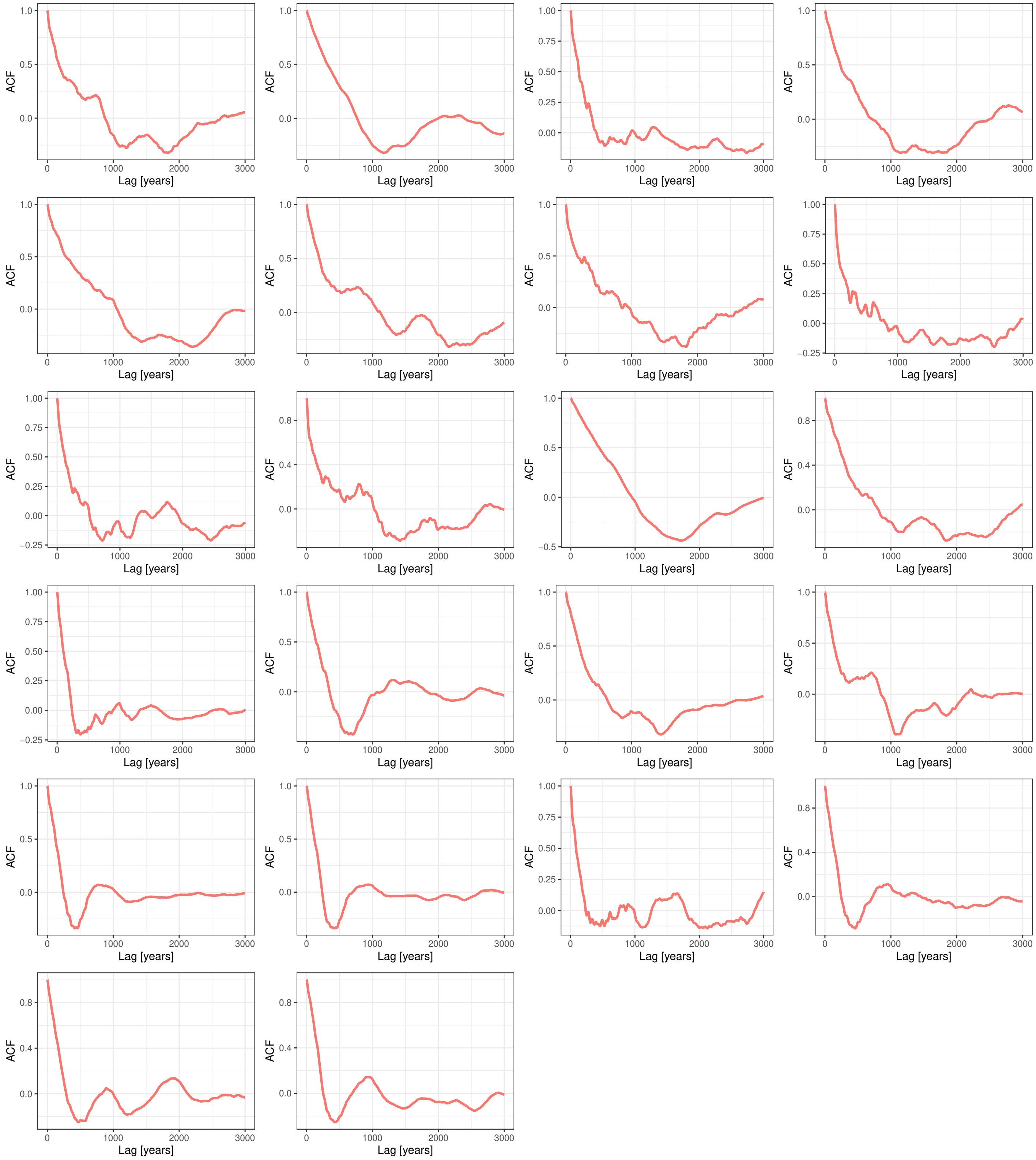

Figure S9: ACFs computed from regional SPDs of radiocarbon dates after detrending. Patterns correspond well to typical timscales of 500-1,000 years for demographic processes.

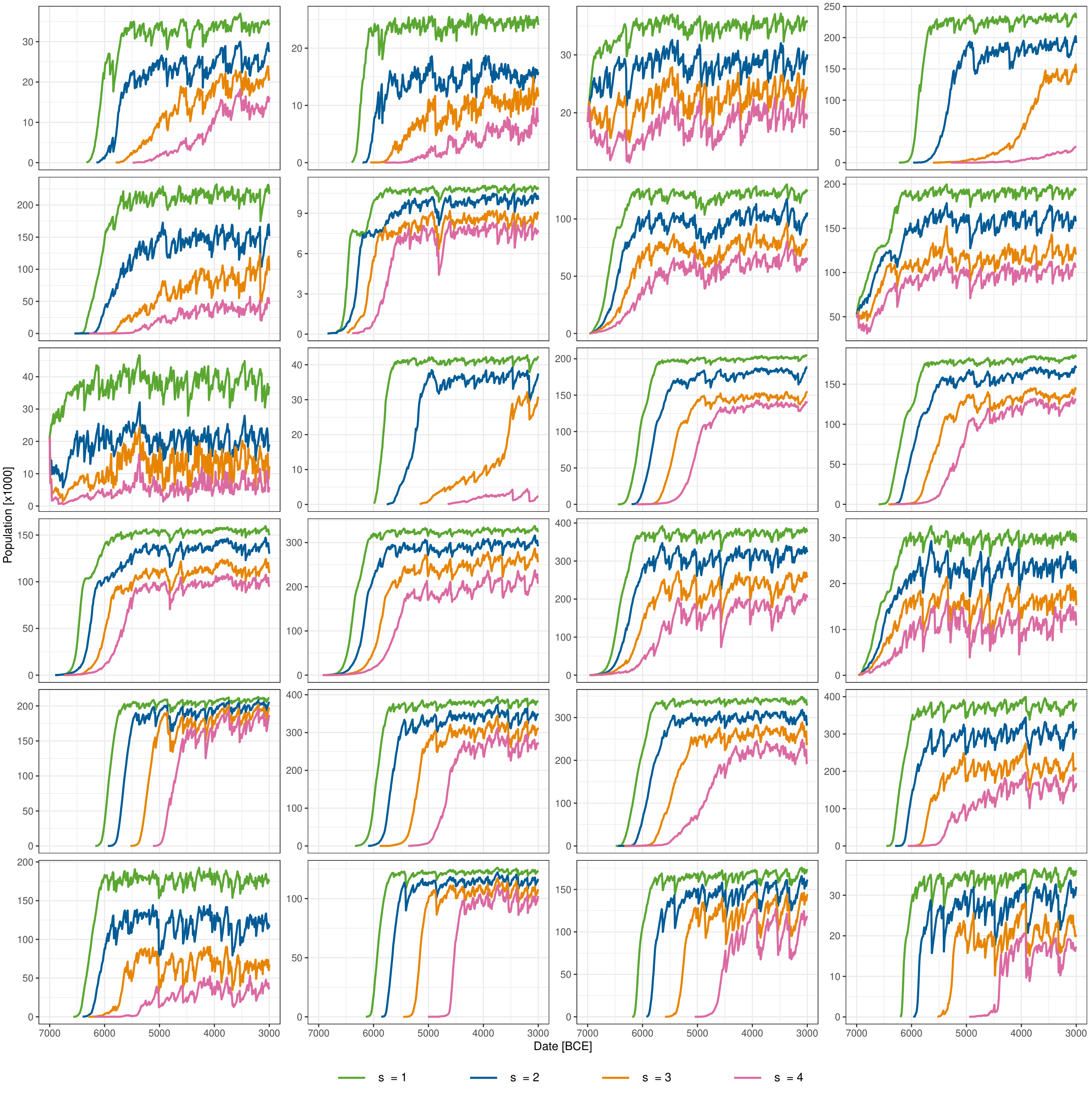

Figure S10: Simulation results in the model including climate variation but not conflict ($p_E = 0$), for main parameters: $G = 40\,\text{km}$, $s = 2$. Panels show regional population in one tiling position among the simulation area.

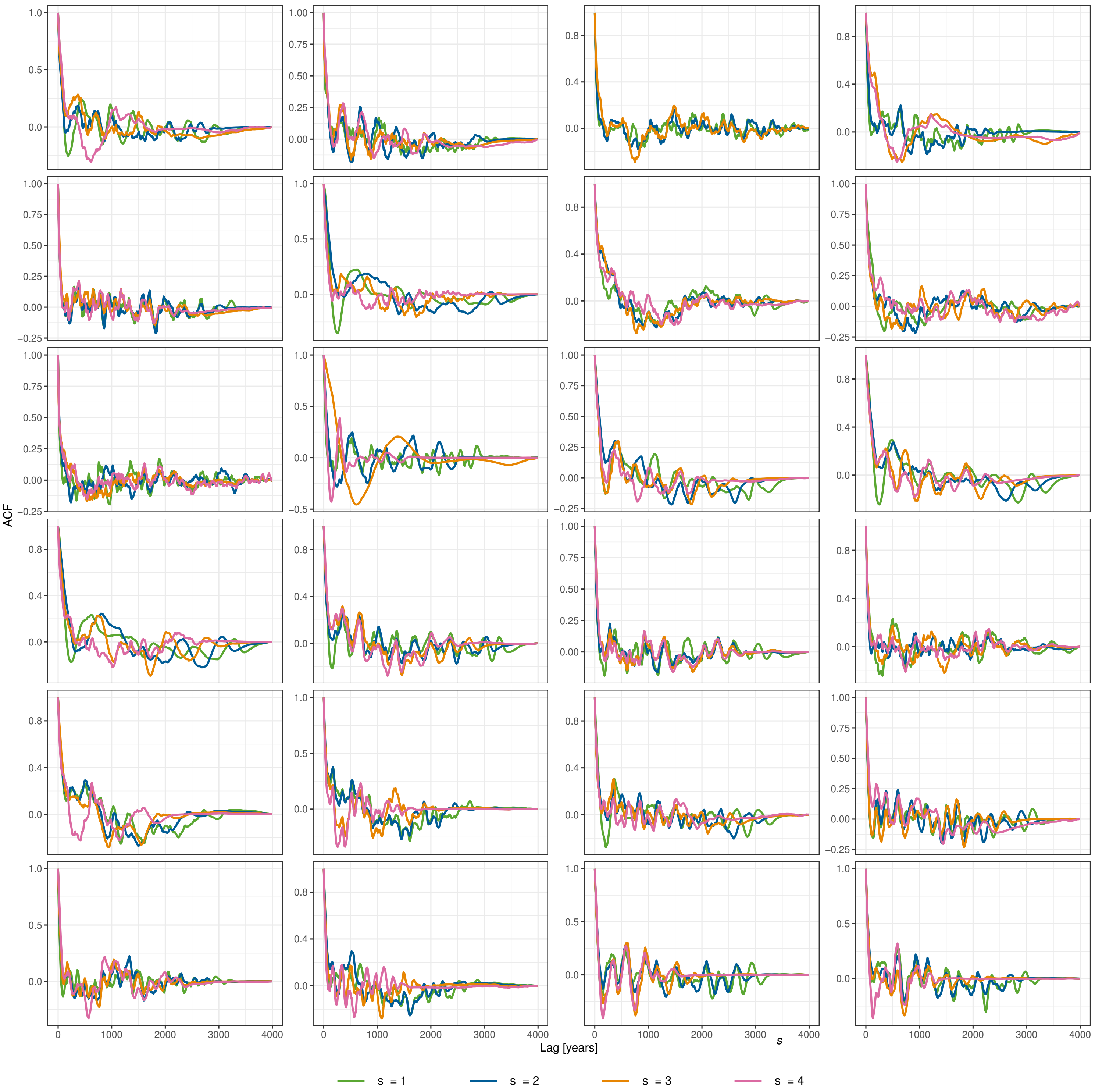

Figure S11: Simulation results in the model including climate variation but not conflict ($p_E = 0$), for main parameters: $G = 40\,\text{km}$, $s = 2$. Panels show ACFs of detrended regional population in one tiling position among the simulation area.

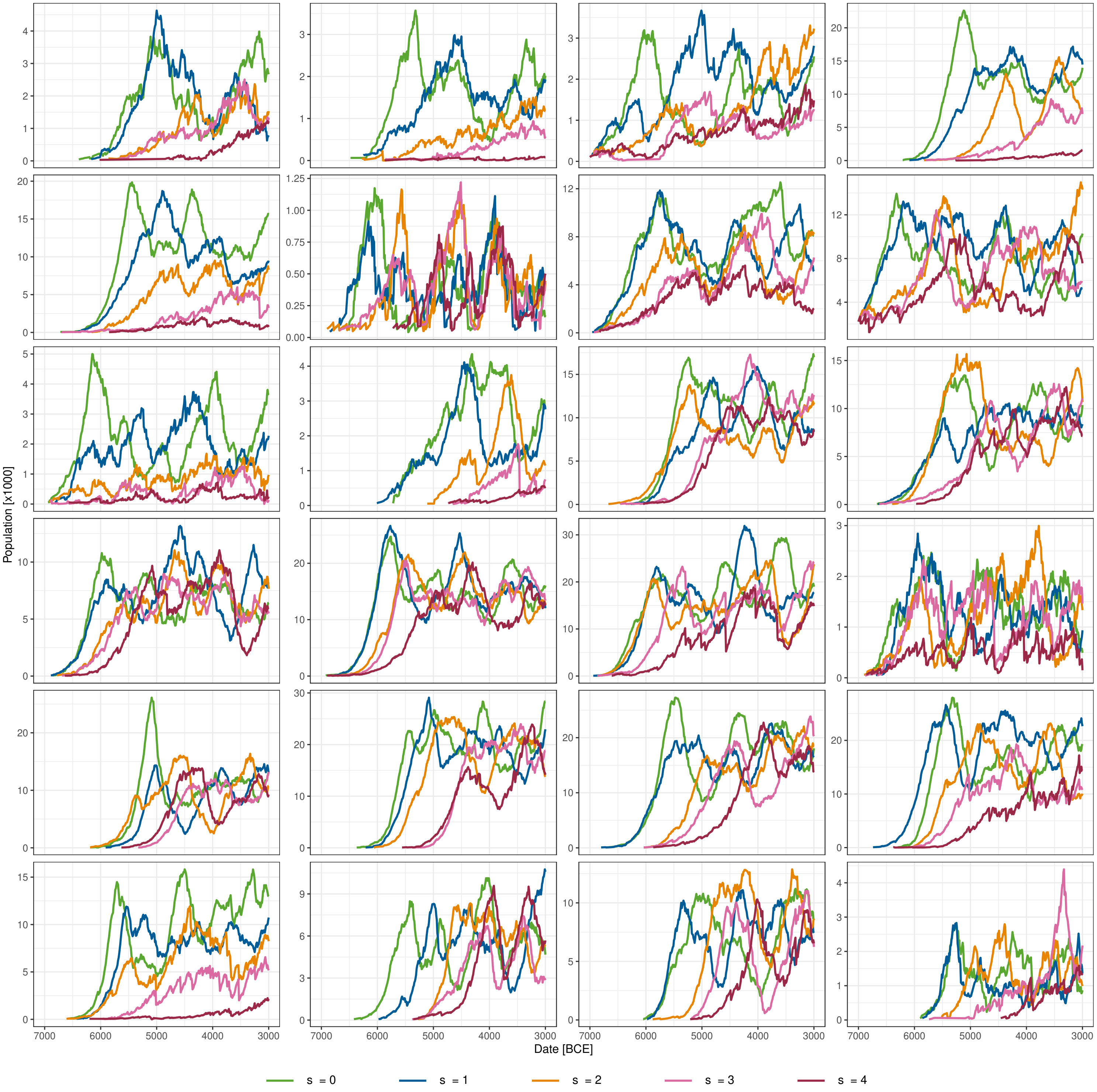

Figure S12: Simulation results in the model variant including both the conflict and climate variation components, for main parameters: $G = 80\,\mathrm{km}$, $s = 2$, $p_E = 0.2$ and $p_A = 1/20$ years. Panels show regional population in one tiling position among the simulation area.

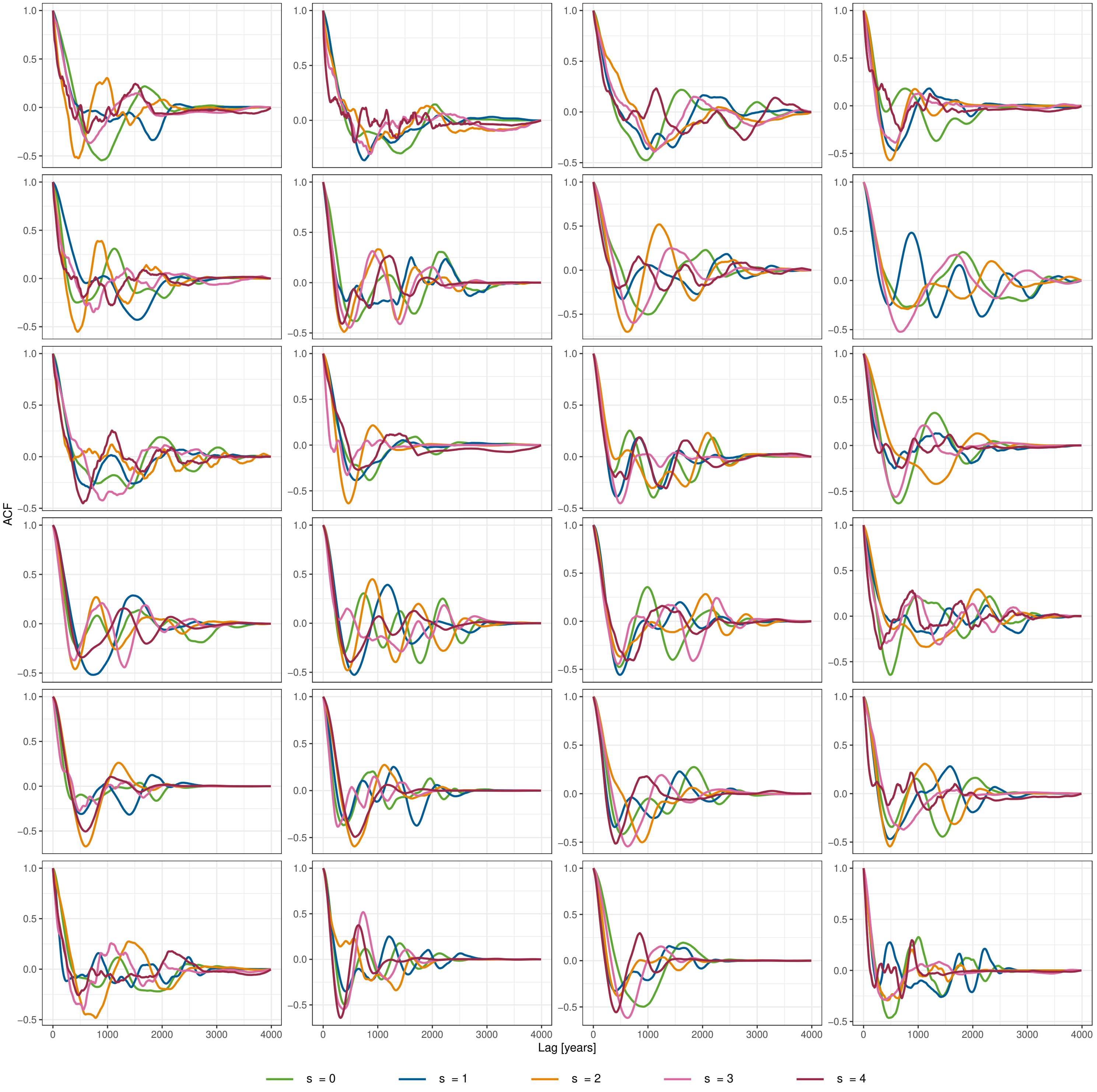

Figure S13: Simulation results in the model including both the conflict and climate variation components, for main parameters: $G = 80\,\mathrm{km}$, $s = 2$, $p_E = 0.2$ and $p_A = 1/20$ years. Panels show ACFs of detrended regional population in one tiling position among the simulation area.

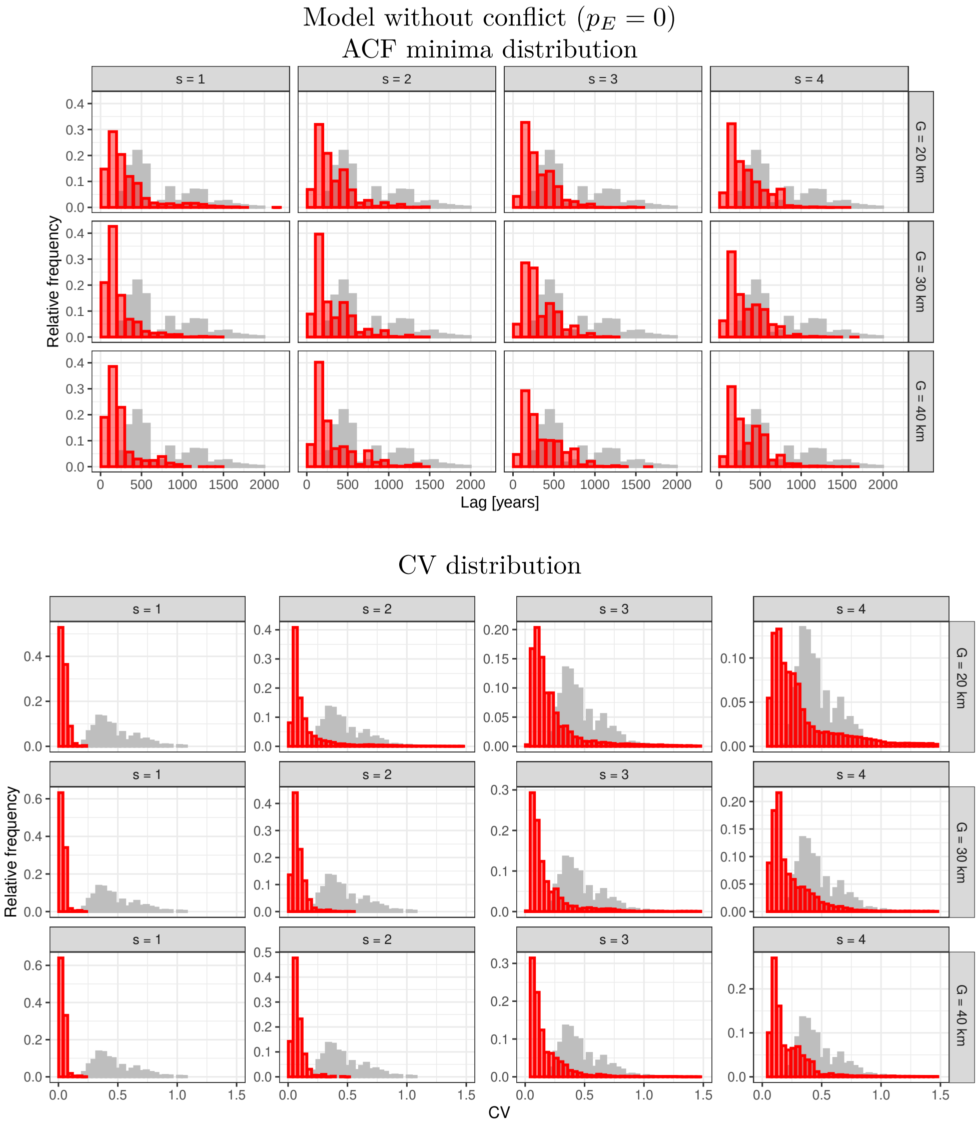

Figure S14: Distribution of the location of the first minimum in regional ACFs (top) and CV values (bottom), model without conflict (i.e. $p_E = 0$).

years, and varied the s and G parameters. In this case, the mode of the distributions does not change significantly, while the shape is affected to some degree: for lower values of s, there is less variance in ACF minima.

In Fig. S17, we show ACF minima and CV distributions for the model variant where density-dependent conflicts happen (i.e. $p_E > 0$), but do not lead to the creation of aggressors ($p_C = 0$; there is no second-order dynamics). We used a parameter of $G = 80\,\text{km}$ and varied the p_E and s parameters.

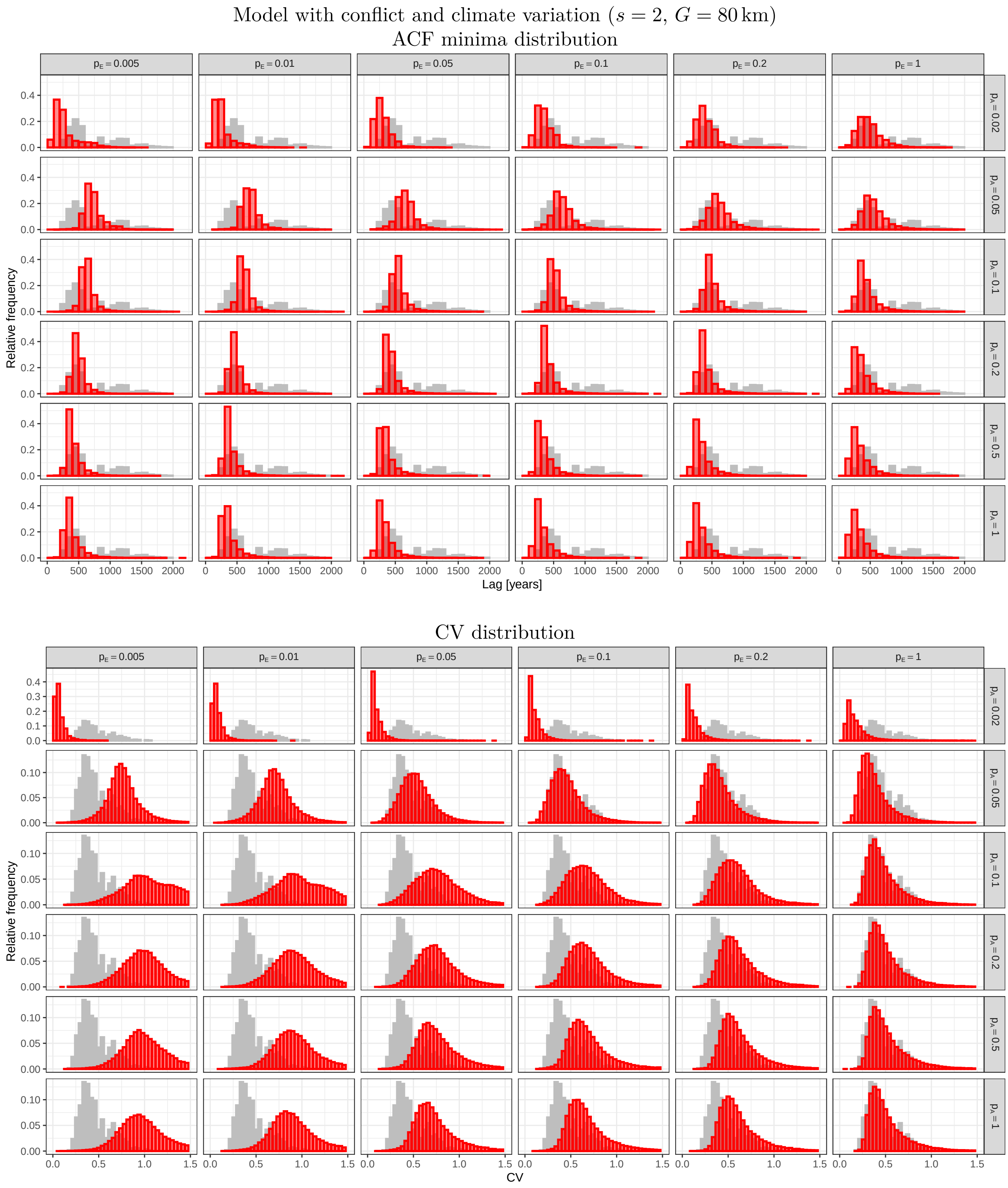

Figure S15: Distribution of the location of the first minimum in regional ACFs (top) and CV values (bottom), model without conflict and climate variation.

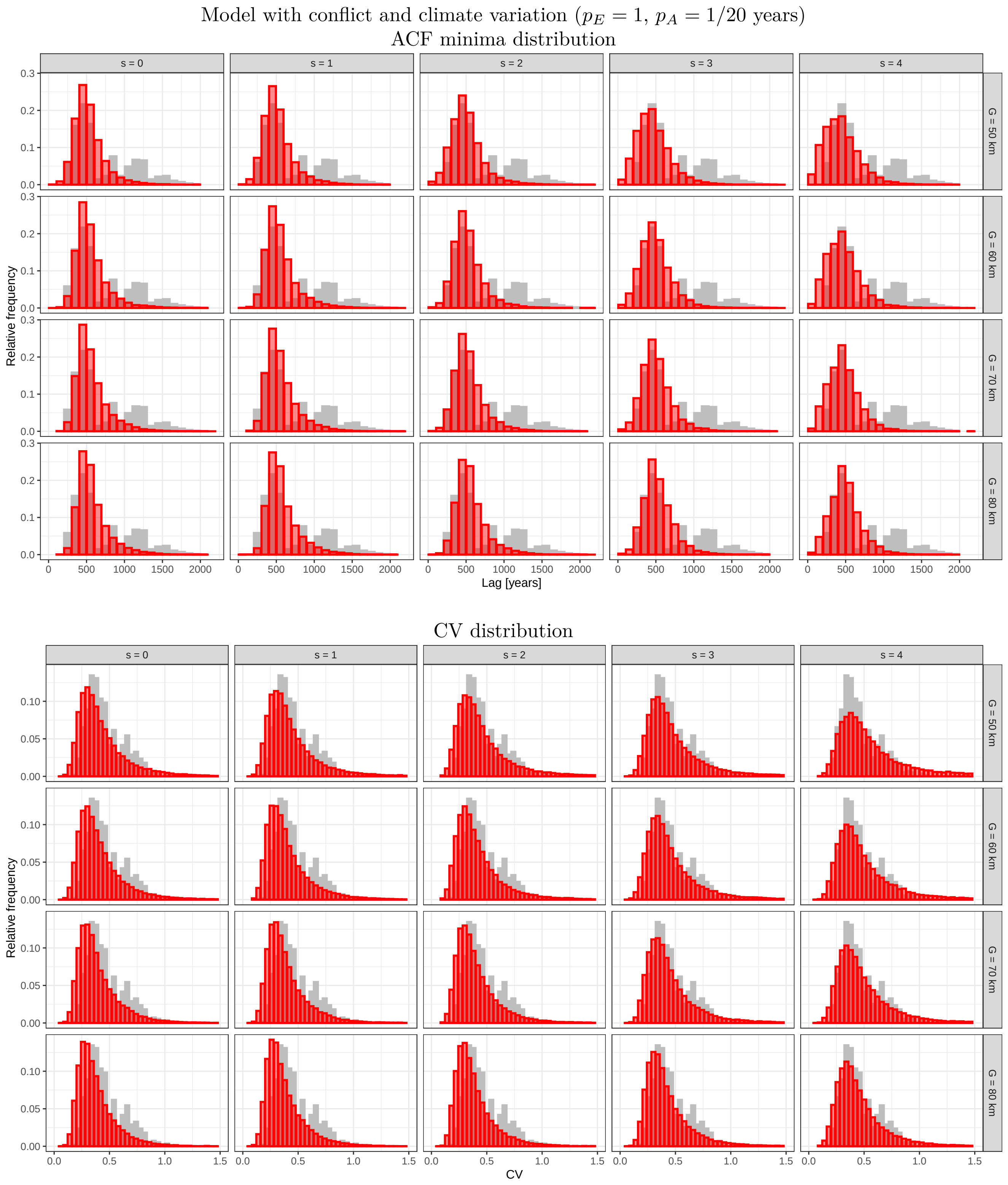

Figure S16: Distribution of the location of the first minimum in regional ACFs (top) and CV values (bottom), model without conflict and climate variation.

Model with conflict and climate variation but without aggressors ($G = 80\,\text{km}$, $p_C = 0$)
ACF minima distribution

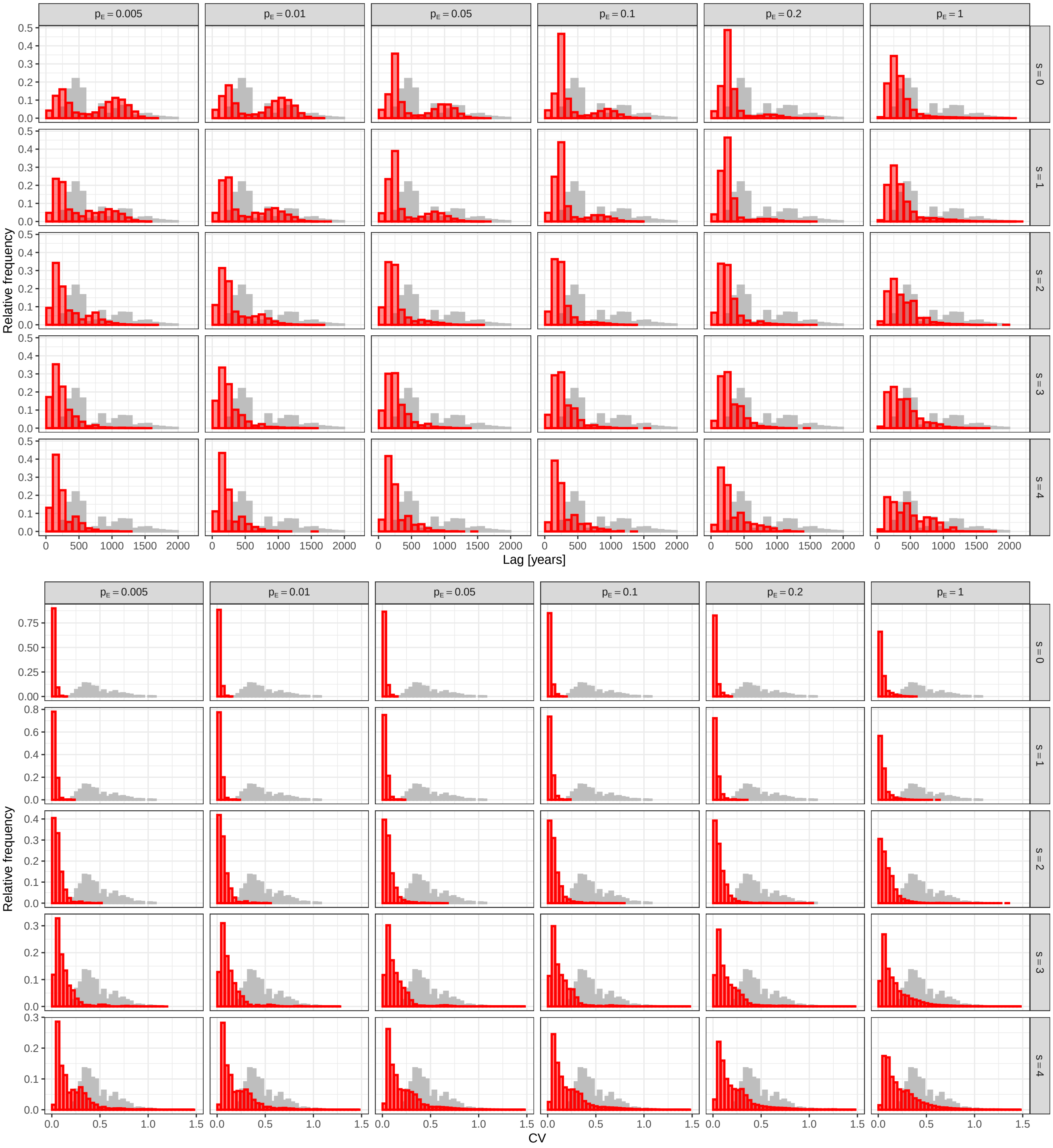

Figure S17: Distribution of the location of the first minimum in regional ACFs (top) and coefficients of variation (bottom), model with conflict and climate, but no aggressors (i.e. first-order interactions), $G = 80\,\text{km}$.

2.5 Additional model variants

Beyond the main model variant, we have investigated the effect of three further choices on model behavior:

- Alternate fission probabilities: instead of a linear dependence on population (Eq. (2) in the main text), we employed the logistic model of group fission from Ref. [23]. Note that even in this case, we scale the maximum fission probability to 0.125, i.e. a group fission is expected to occur once every 8 years. We denote the original variant by (A) and the alternate case by (B).
- Initial group migration for aggressors: contrary to the original model where selecting an already occupied cell as a migration target results in a conflict without migration, we have considered an alternate case when a migration still happens, and newly created aggressors take over the target cell. After this, they still remain in-place (contrary to the alternate, "roaming aggressors" model variant). We denote the original variant by (C) and the alternate case by (D).
- Aggressors do not consider the population of potential target cells: contrary to the original model where aggressors prefer to attack cells with larger population, in this alternate variant, target cells' population plays no role in their selection. Note that the probability of winning a conflict is unaffected. We denote the original variant by (E) and the alternate case by (F).

The combination of these gives in total 8 possible model variants, denoted by the combination of letters defined above, e.g. ACE denotes the original model variant, while BCF denotes a version where fission probabilities use the logistic form and aggressors are not affected by target cells' population in their choices. We display main results for these model variants for one combination of parameters ($G = 80\,\mathrm{km}$, $s = 2$, $p_A = 0.1$ and $p_E = 0.2$) in Fig. S18. Each panel shows results from 100 repeated simulation realizations. Based on these results, we conclude that differences among these model variants are insignificant.

2.6 Impact of varying additional parameters on ACF distributions

In Figs. S19-S41, we display additional results for the distribution of ACF minima where we additionally varied the s and G parameters. All of these results are based on 100 repeated realizations of the simulation with the given parameter values and compiling aggregate histograms of the results among these. Figs. S19-S28 show results for a model variant with alternate interactions where aggressors regularly relocate to attacked cells. Figs. S29-S38 show results for the main model variant (stationary aggressors). Figs. S39-S41 show results for the model variants where conflicts happen without creating aggressors.

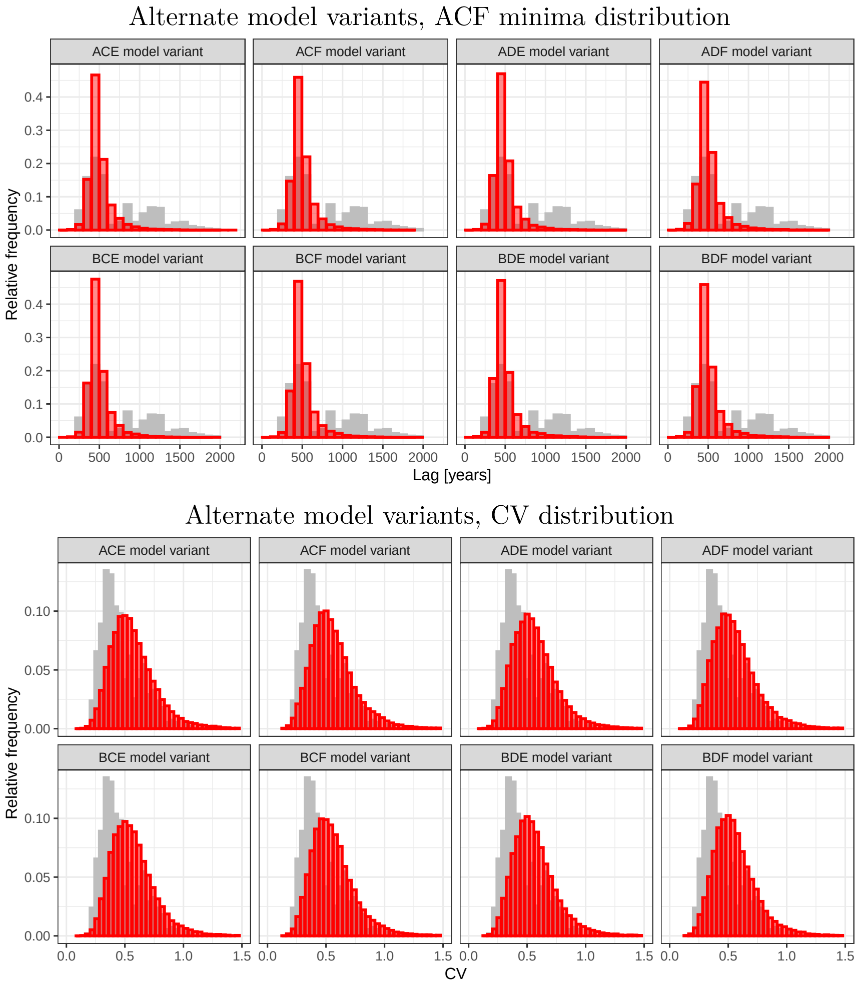

Figure S18: Distribution of the location of the first minimum in regional ACFs (top) and coefficients of variation (bottom), for additional model variants ($G = 80\,\text{km}$, $s = 2$, $p_A = 0.1$ and $p_E = 0.2$).

Roaming aggressors, $s = 0$, $G = 50$ km

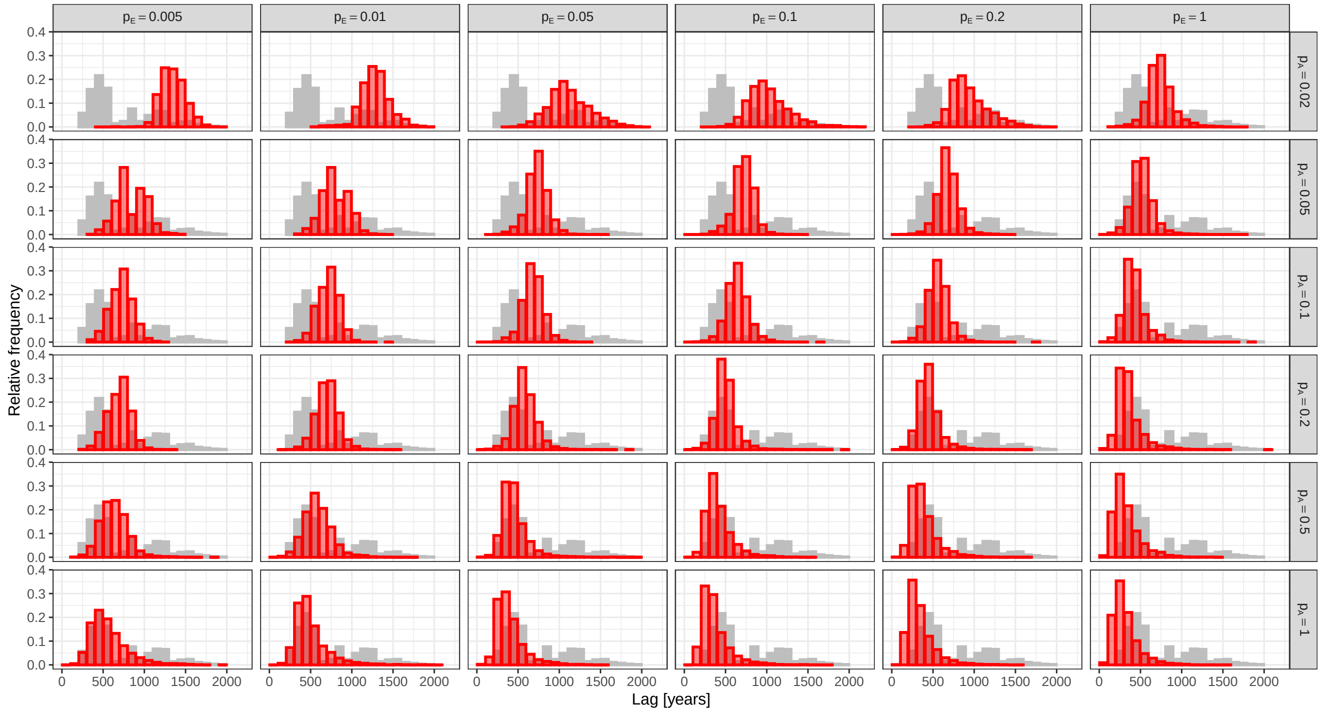

Roaming aggressors, $s = 0$, $G = 60$ km

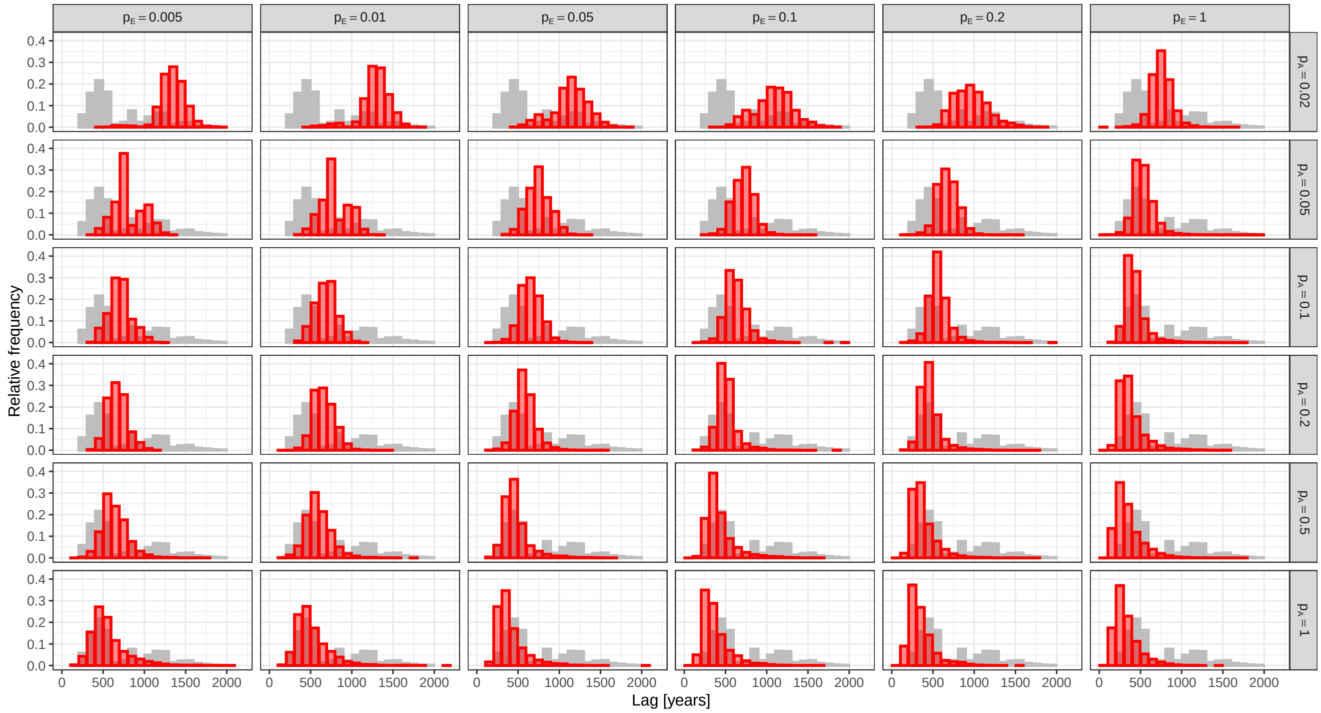

Figure S19: Distribution of the location of the first minimum in regional ACFs, model with moving aggressors, $s = 0$ (i.e. no climatic variation in agricultural productivity), $G = 50$ and 60 km (top and bottom panels respectively).

Roaming aggressors, $s = 0$, $G = 70\,\mathrm{km}$

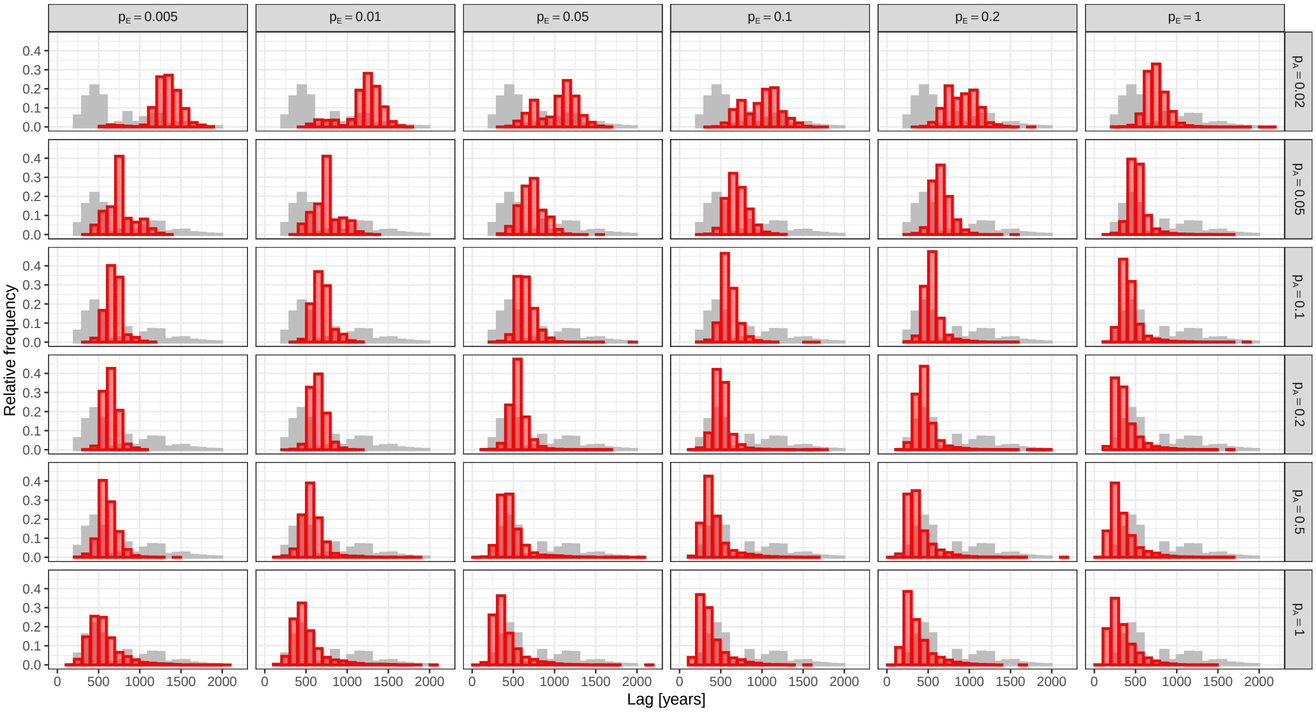

Roaming aggressors, $s = 0$, $G = 80\,\mathrm{km}$

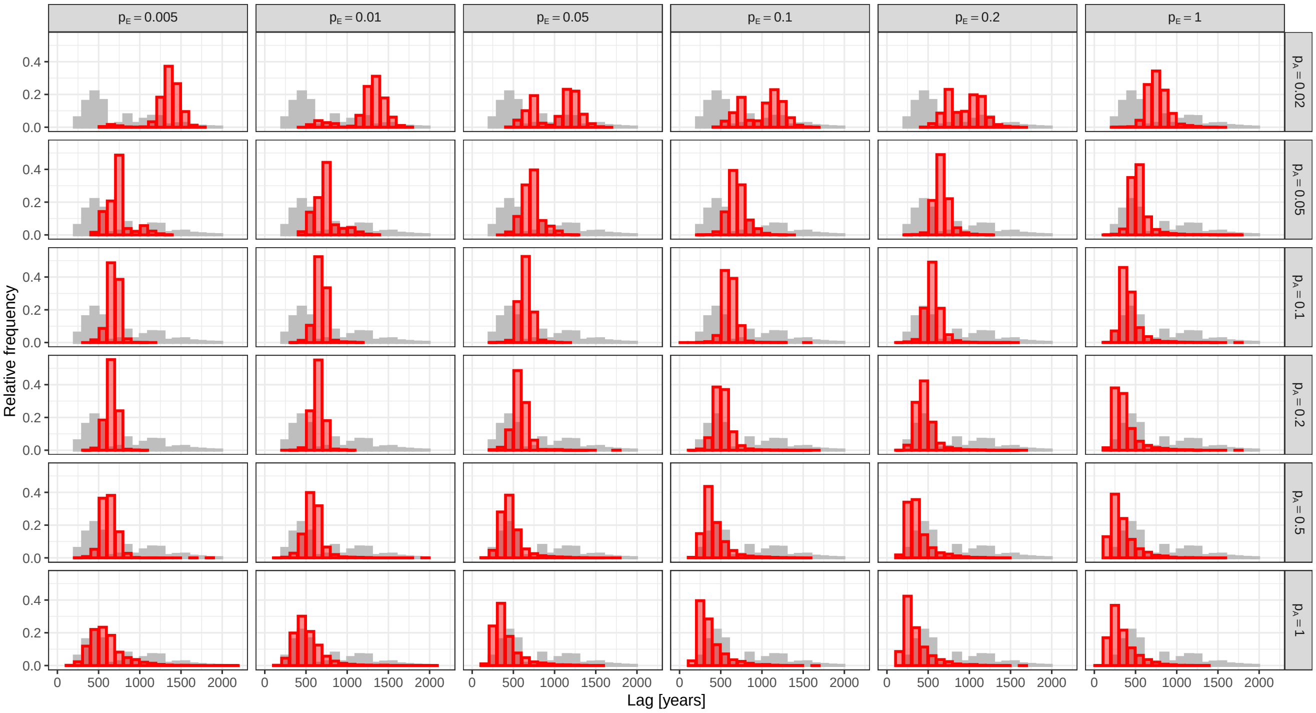

Figure S20: Distribution of the location of the first minimum in regional ACFs, model with moving aggressors, $s = 0$ (i.e. no climatic variation in agricultural productivity), $G = 70$ and $80\,\mathrm{km}$ (top and bottom panels respectively).

Roaming aggressors, $s = 1$, $G = 50\,\mathrm{km}$

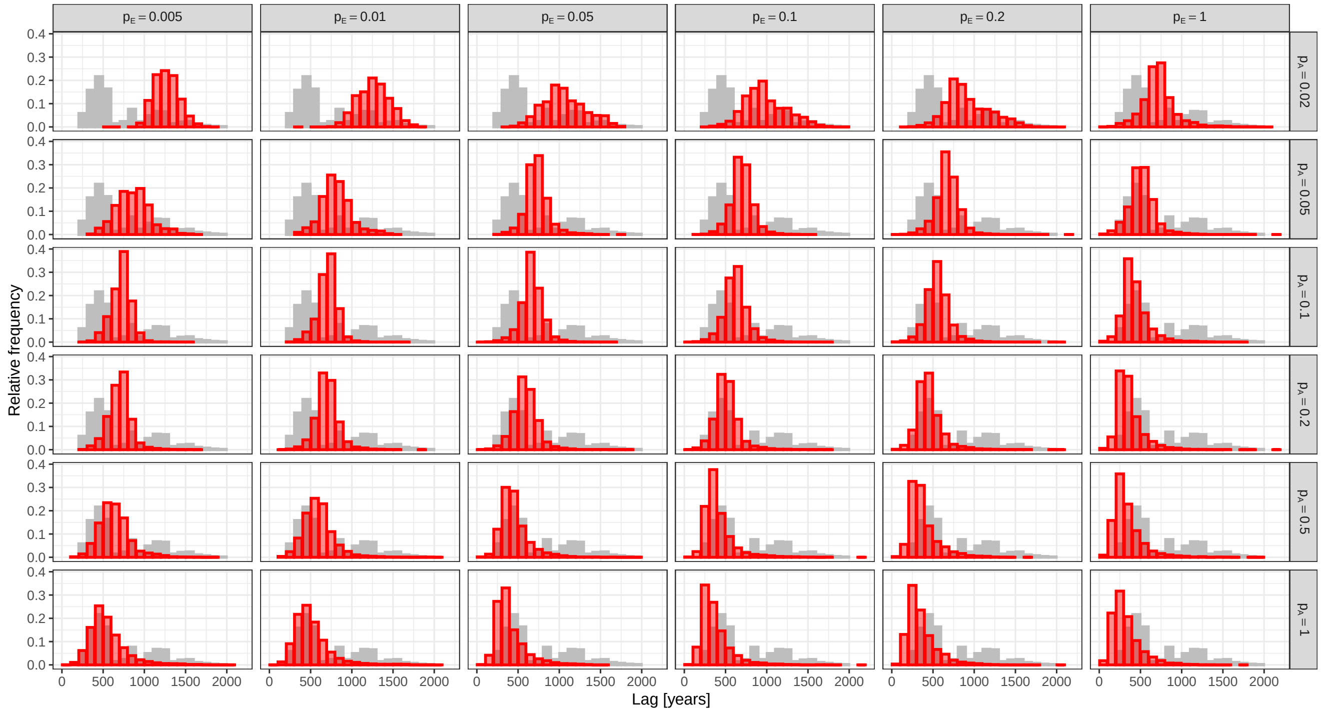

Roaming aggressors, $s = 1$, $G = 60\,\mathrm{km}$

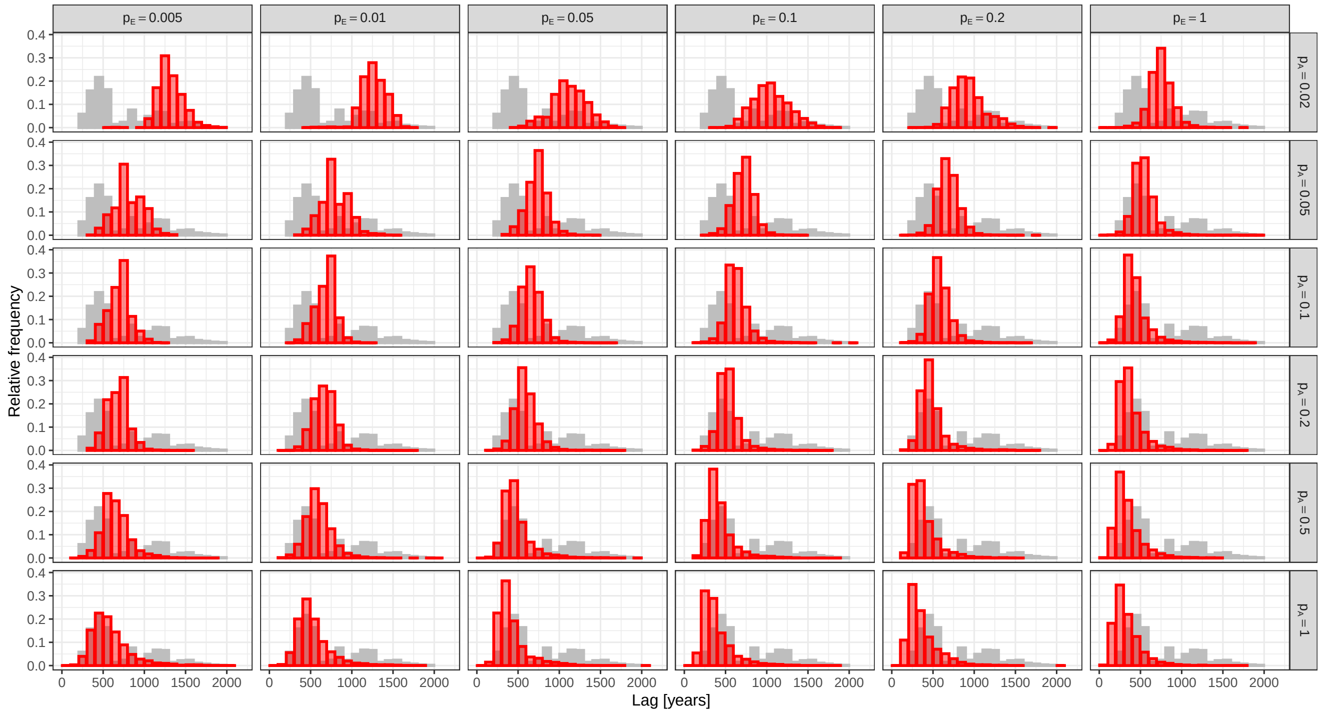

Figure S21: Distribution of the location of the first minimum in regional ACFs, model with moving aggressors, $s = 1$, $G = 50$ and $60\,\mathrm{km}$ (top and bottom panels respectively).

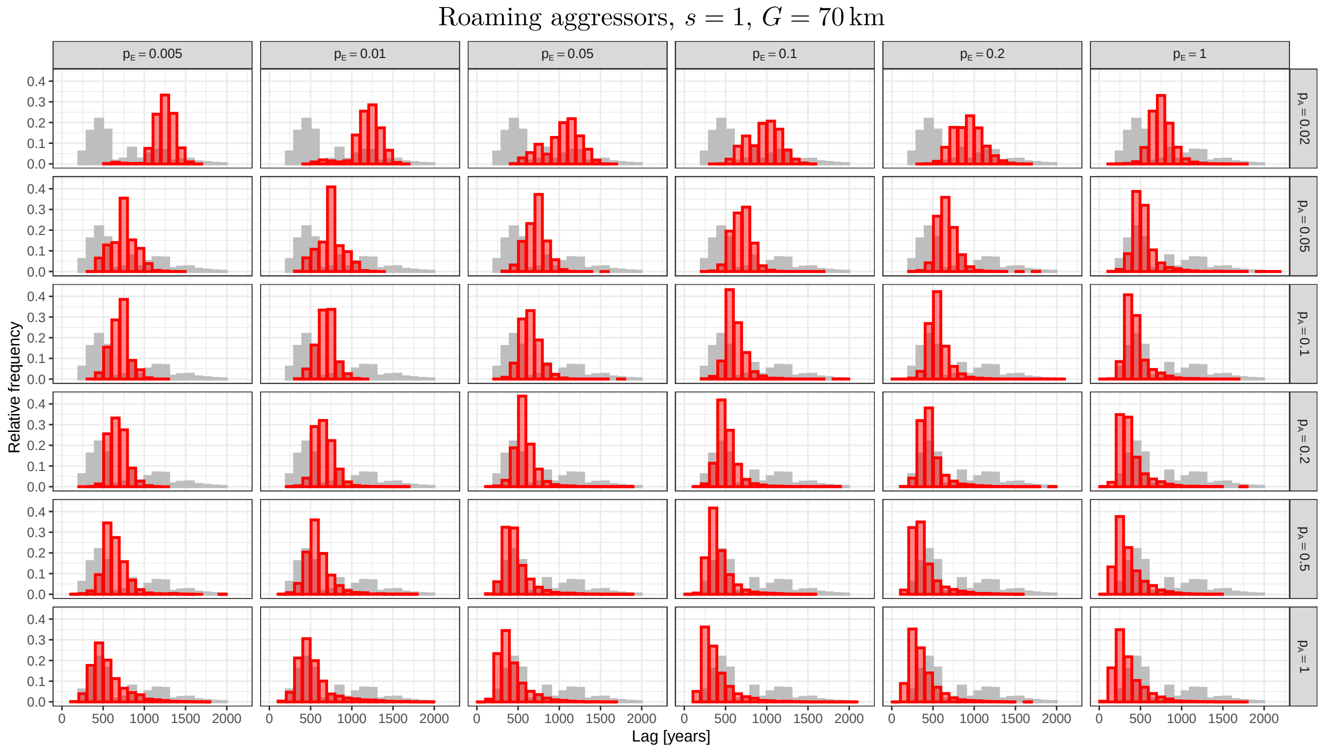

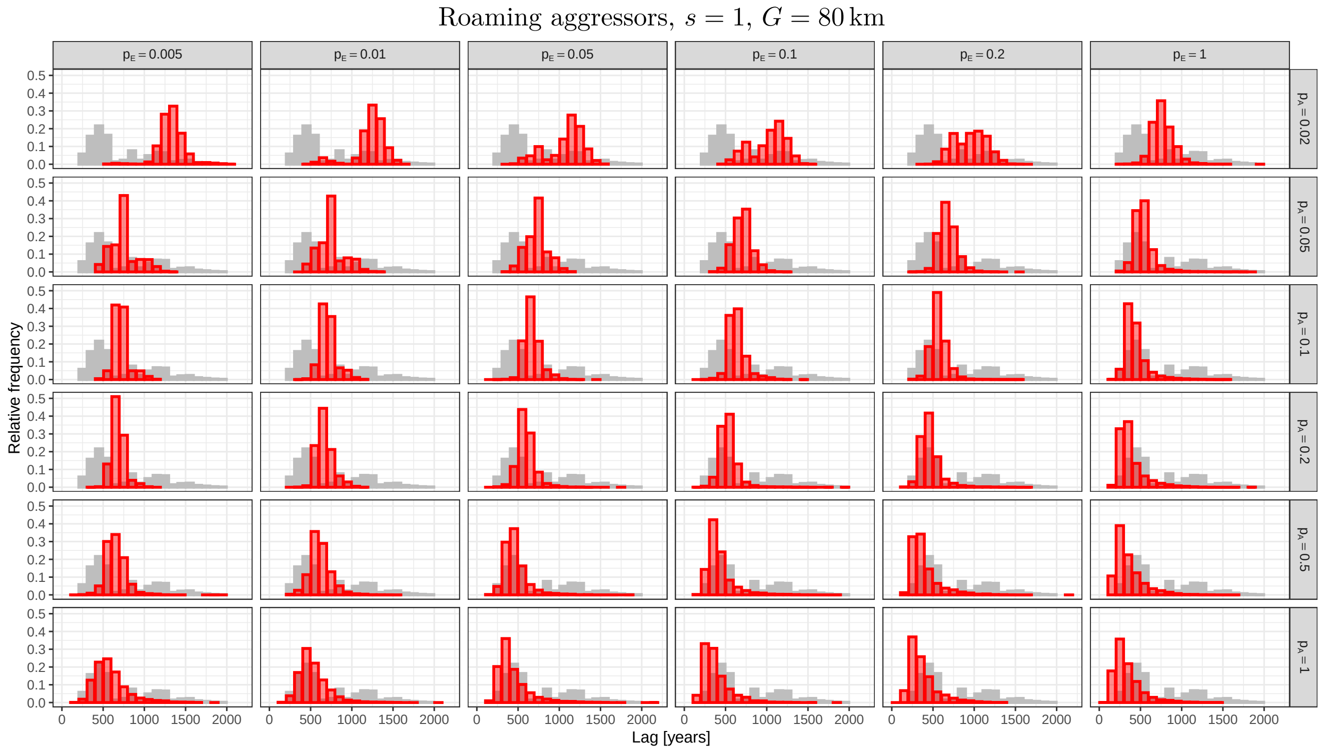

Figure S22: Distribution of the location of the first minimum in regional ACFs, model with moving aggressors, $s = 1$, $G = 70$ and 80 km (top and bottom panels respectively).

Roaming aggressors, $s = 2$, $G = 50\,\text{km}$

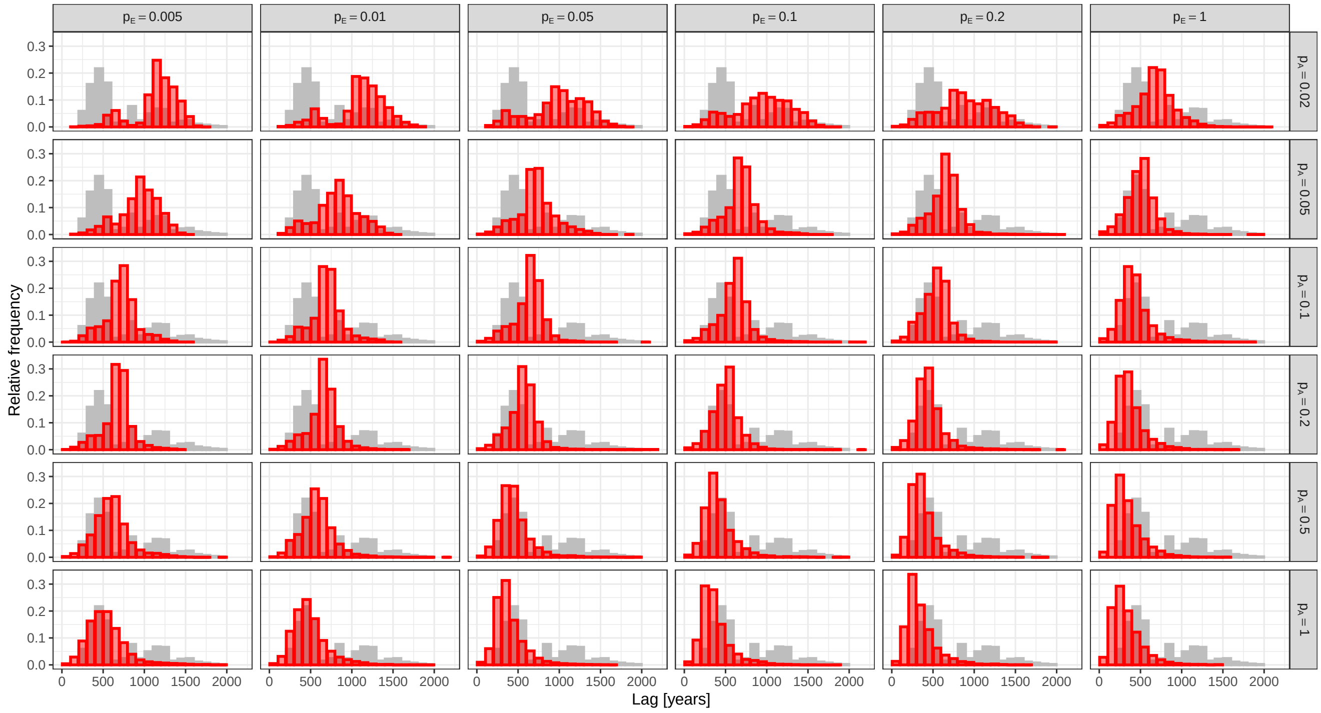

Roaming aggressors, $s = 2$, $G = 60\,\text{km}$

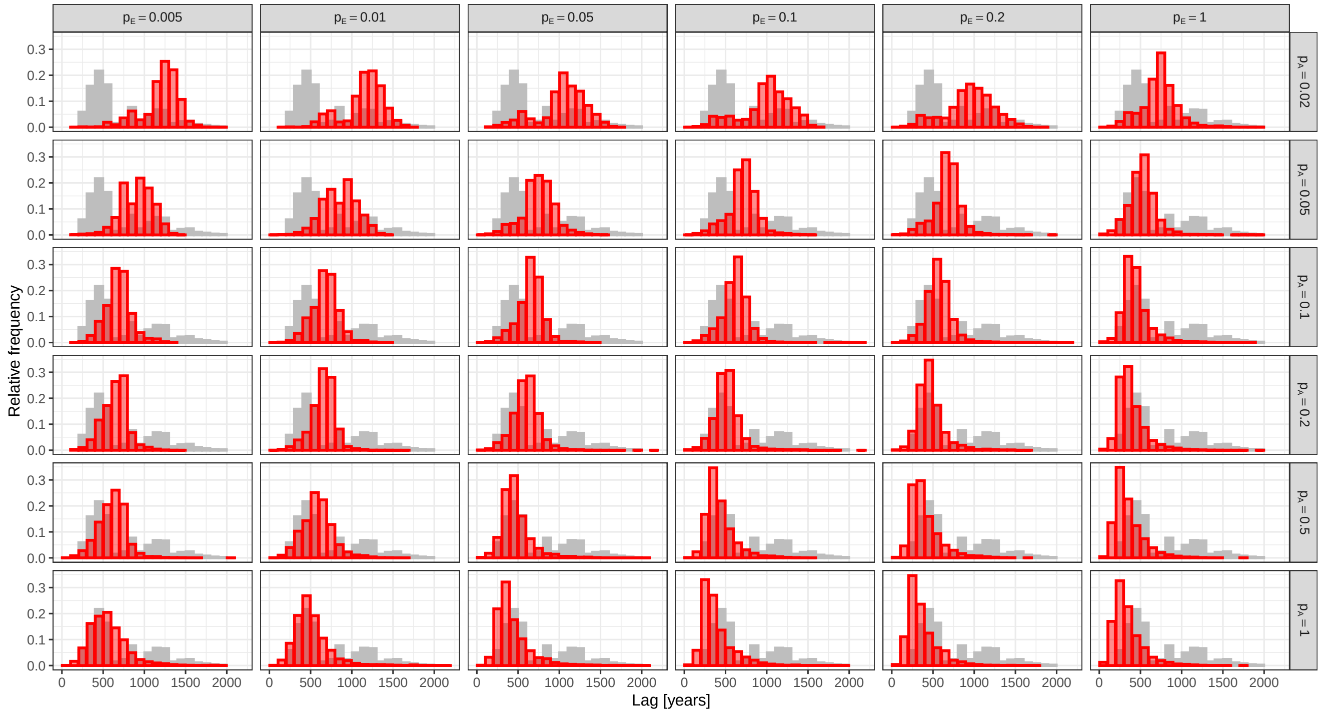

Figure S23: Distribution of the location of the first minimum in regional ACFs, model with moving aggressors, $s = 2$, $G = 50$ and $60\,\text{km}$ (top and bottom panels respectively).

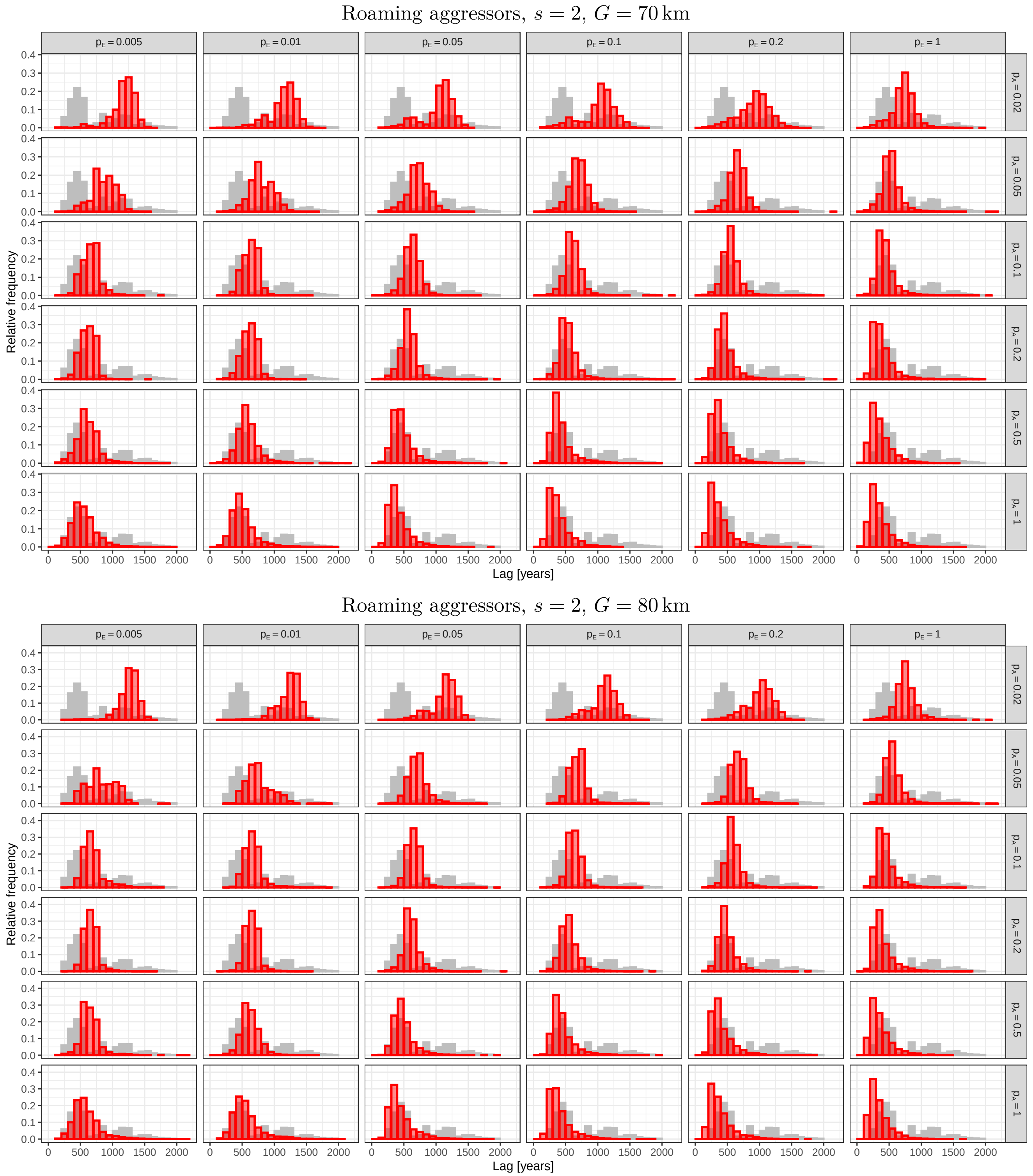

Figure S24: Distribution of the location of the first minimum in regional ACFs, model with moving aggressors, $s = 2$, $G = 70$ and 80 km (top and bottom panels respectively).

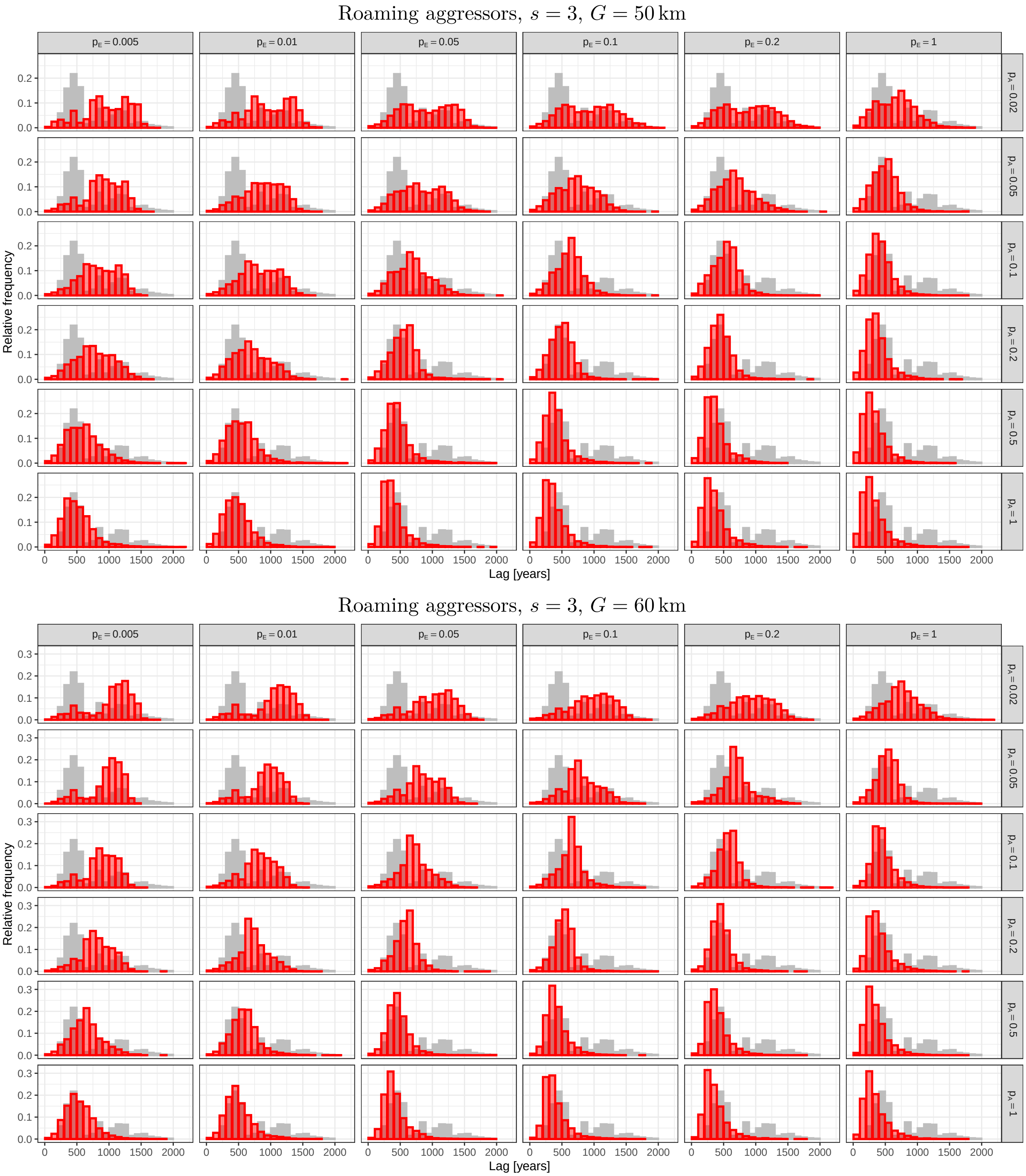

Figure S25: Distribution of the location of the first minimum in regional ACFs, model with moving aggressors, $s = 3$, $G = 50$ and $60\,\mathrm{km}$ (top and bottom panels respectively).

Roaming aggressors, $s = 3$, $G = 70\,\text{km}$

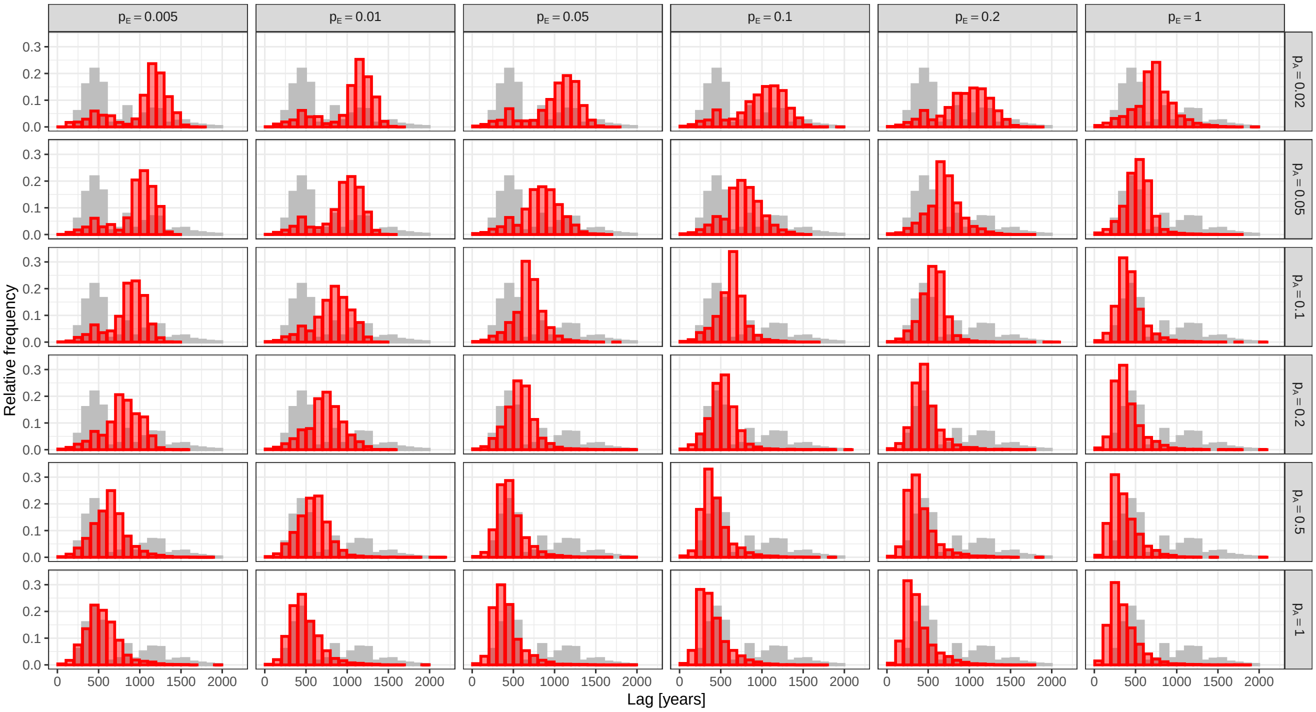

Roaming aggressors, $s = 3$, $G = 80\,\text{km}$

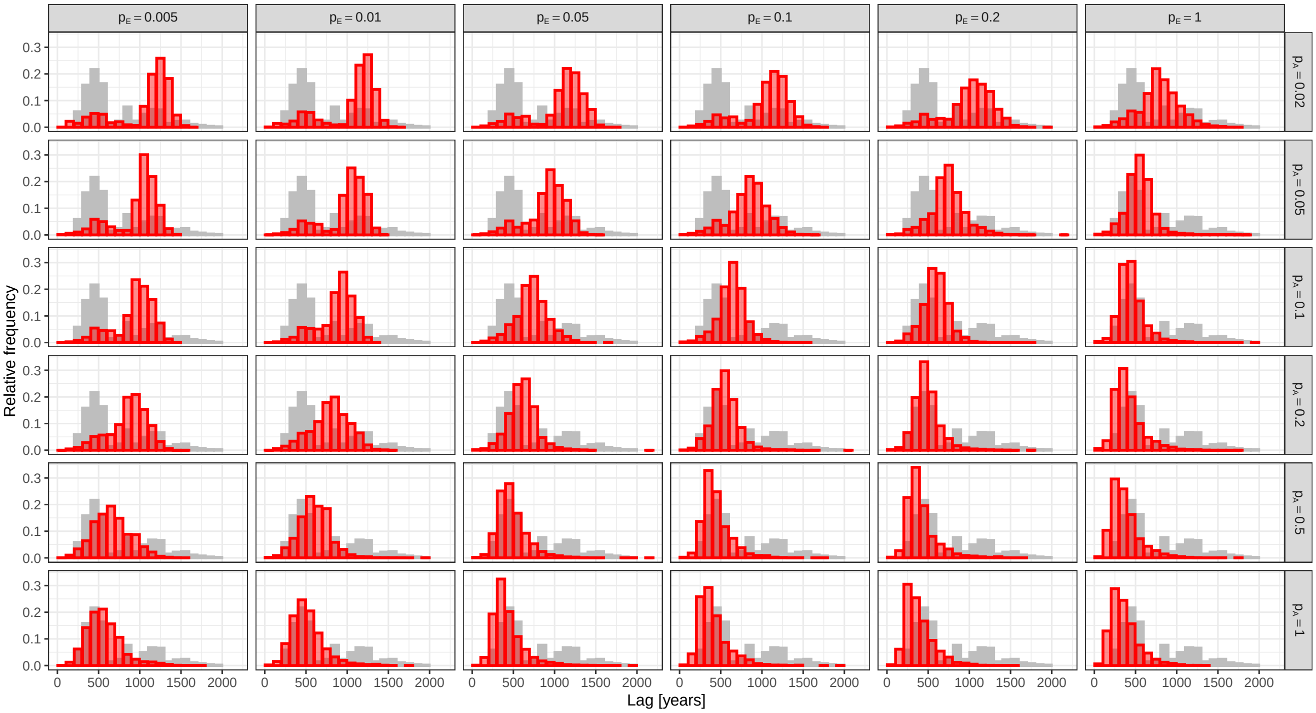

Figure S26: Distribution of the location of the first minimum in regional ACFs, model with moving aggressors, $s = 3$, $G = 70$ and $80\,\text{km}$ (top and bottom panels respectively).

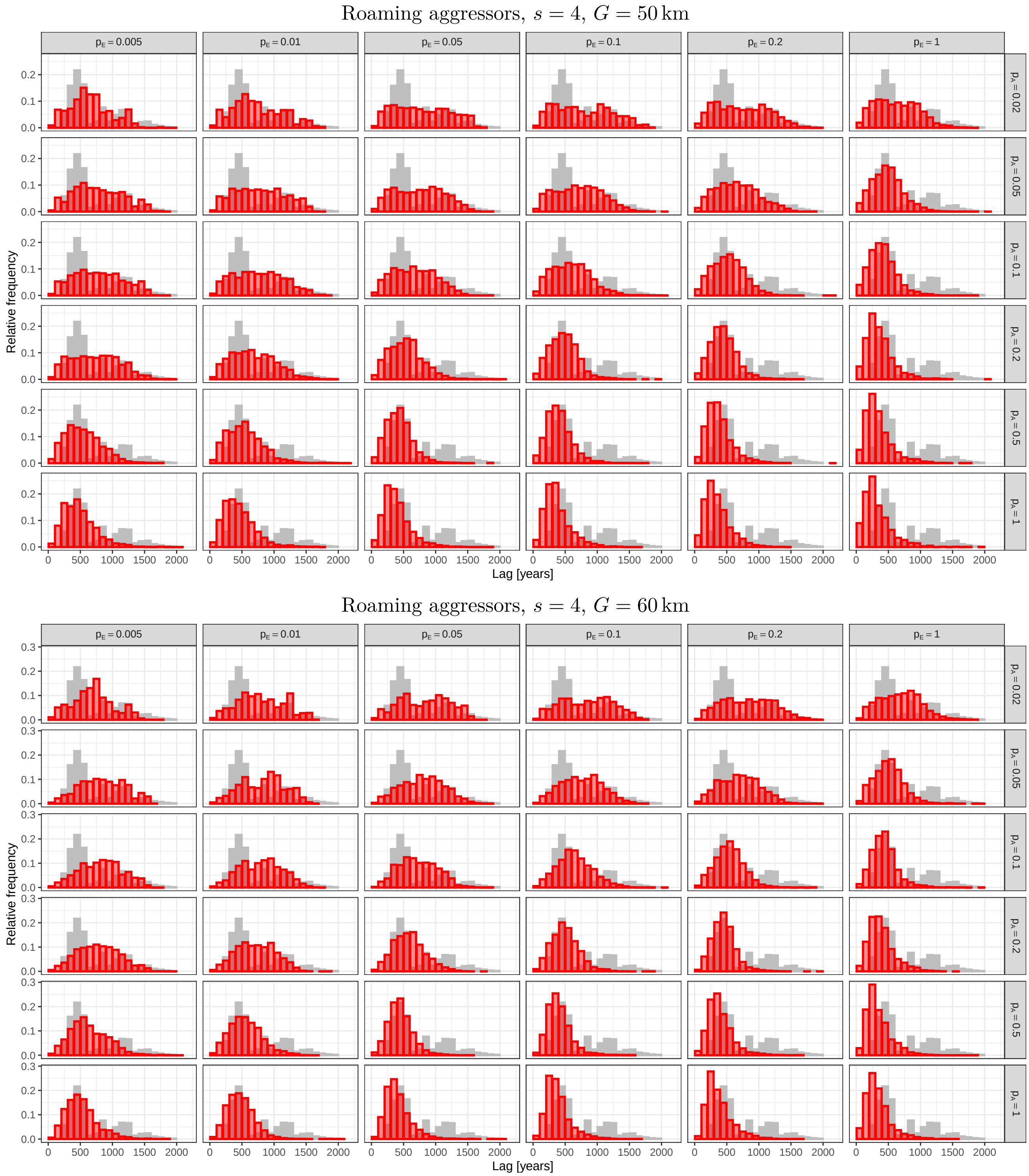

Figure S27: Distribution of the location of the first minimum in regional ACFs, model with moving aggressors, $s = 4$, $G = 50$ and 60 km (top and bottom panels respectively).

Roaming aggressors, $s = 4$, $G = 70\,\mathrm{km}$

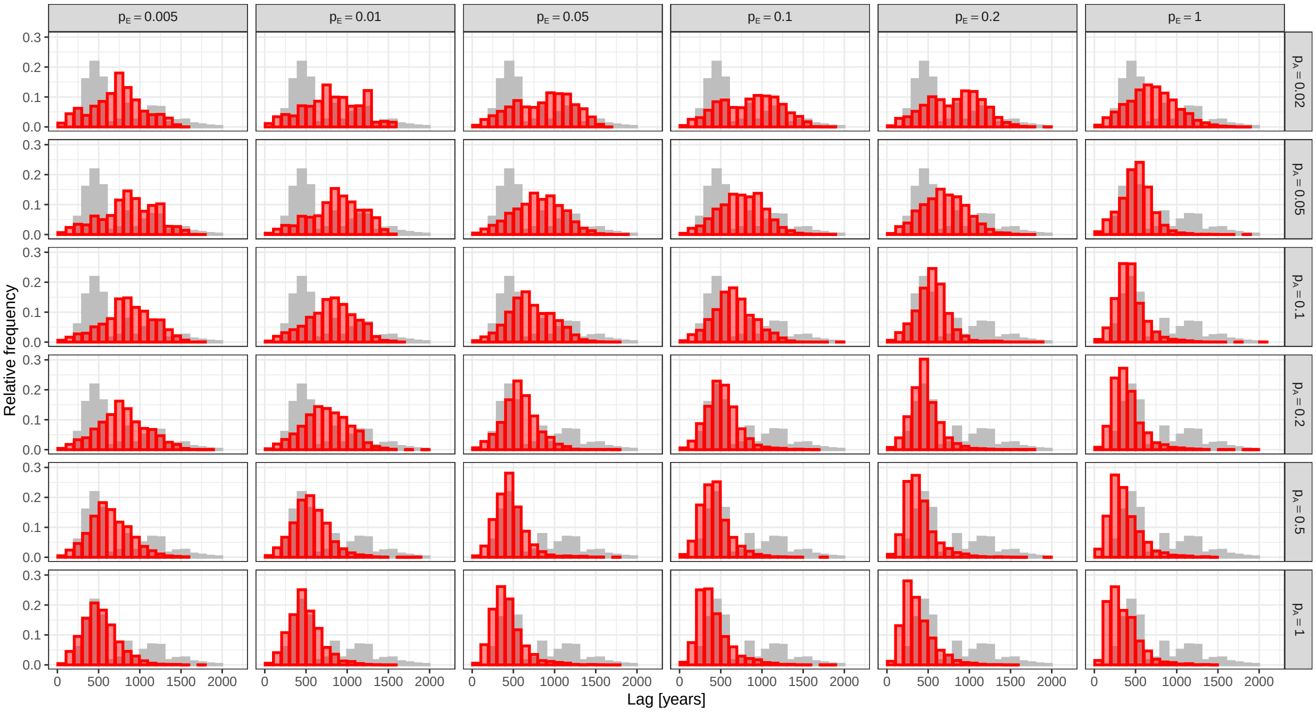

Roaming aggressors, $s = 4$, $G = 80\,\mathrm{km}$

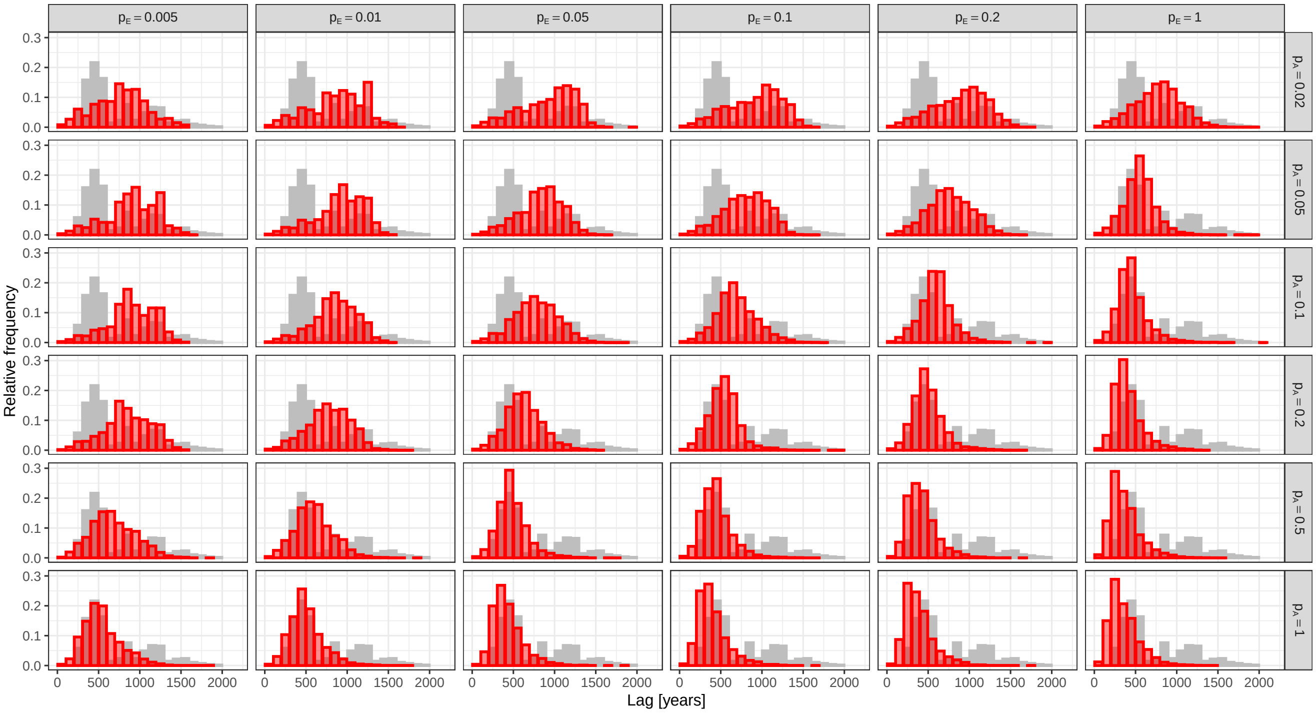

Figure S28: Distribution of the location of the first minimum in regional ACFs, model with moving aggressors, $s = 4$, $G = 70$ and 80 km (top and bottom panels respectively).

Stationary aggressors, $s = 0$, $G = 50\,\text{km}$

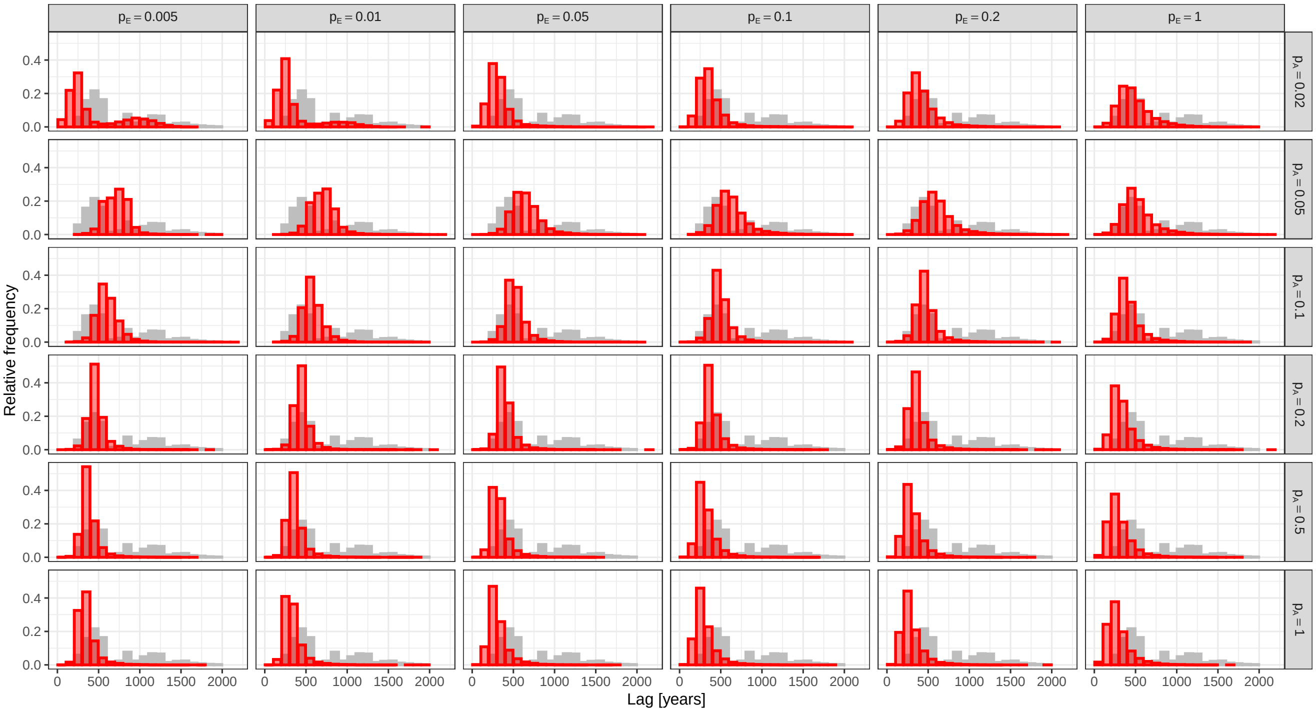

Stationary aggressors, $s = 0$, $G = 60\,\text{km}$

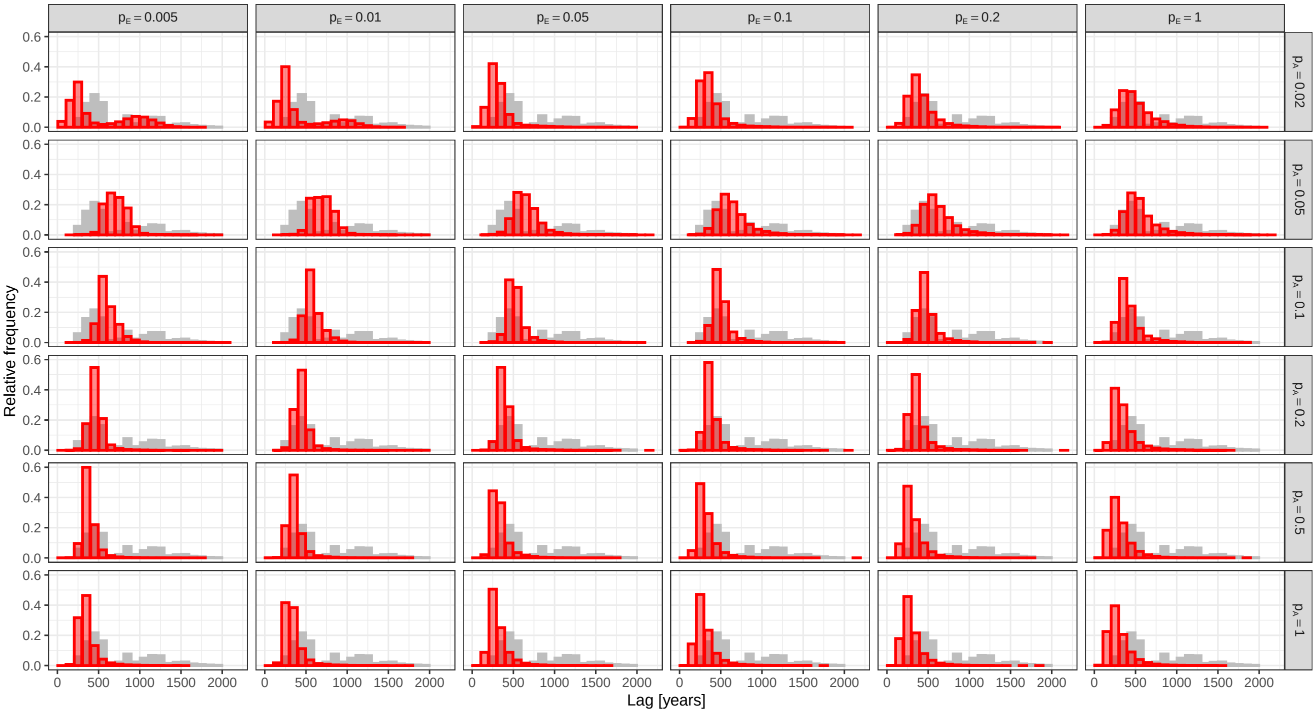

Figure S29: Distribution of the location of the first minimum in regional ACFs, model with stationary aggressors, $s = 0$ (i.e. no climatic variation in agricultural productivity), $G = 50$ and $60\,\text{km}$ (top and bottom panels respectively).

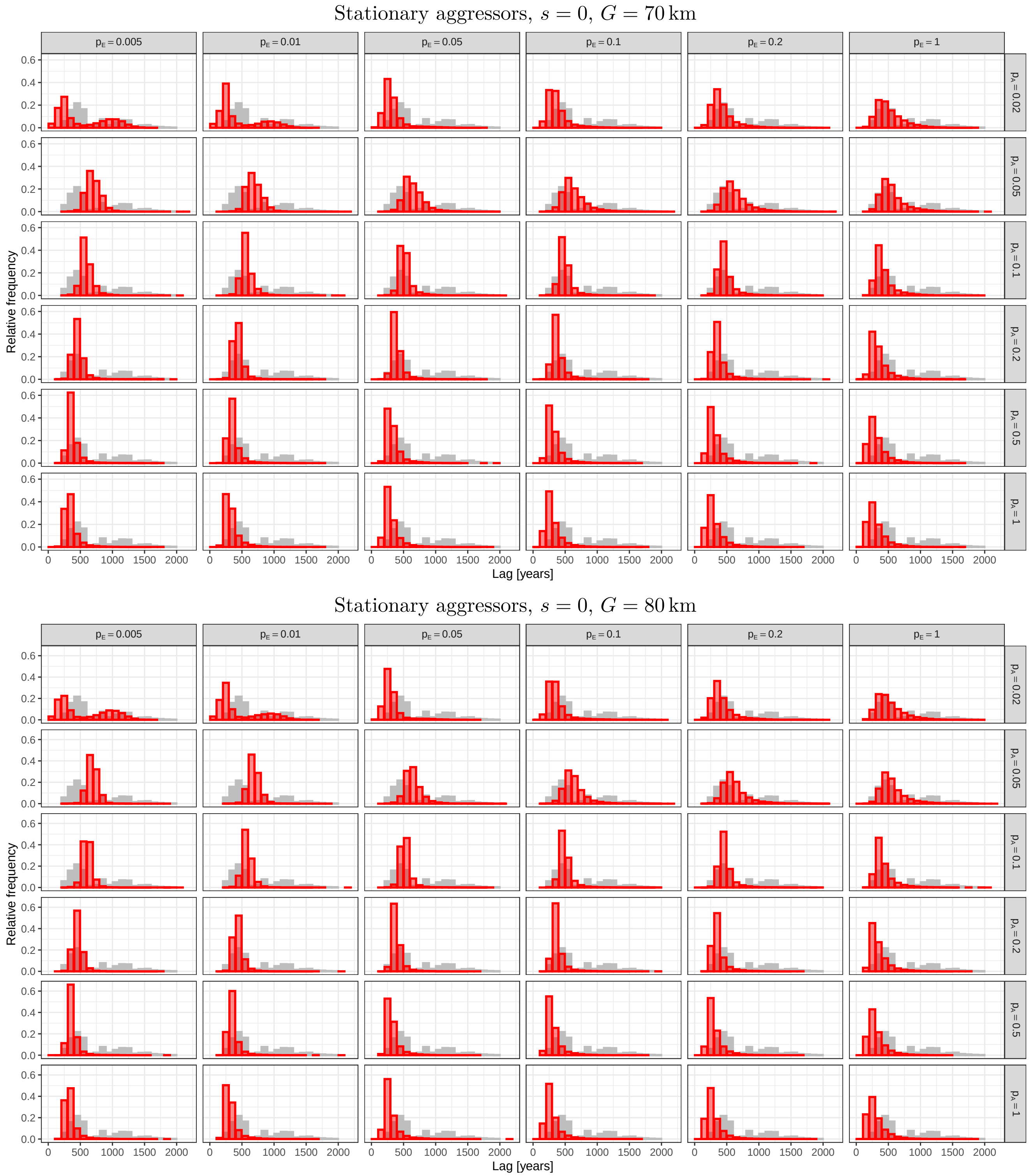

Figure S30: Distribution of the location of the first minimum in regional ACFs, model with stationary aggressors, $s = 0$ (i.e. no climatic variation in agricultural productivity), $G = 70$ and $80\,\mathrm{km}$ (top and bottom panels respectively).

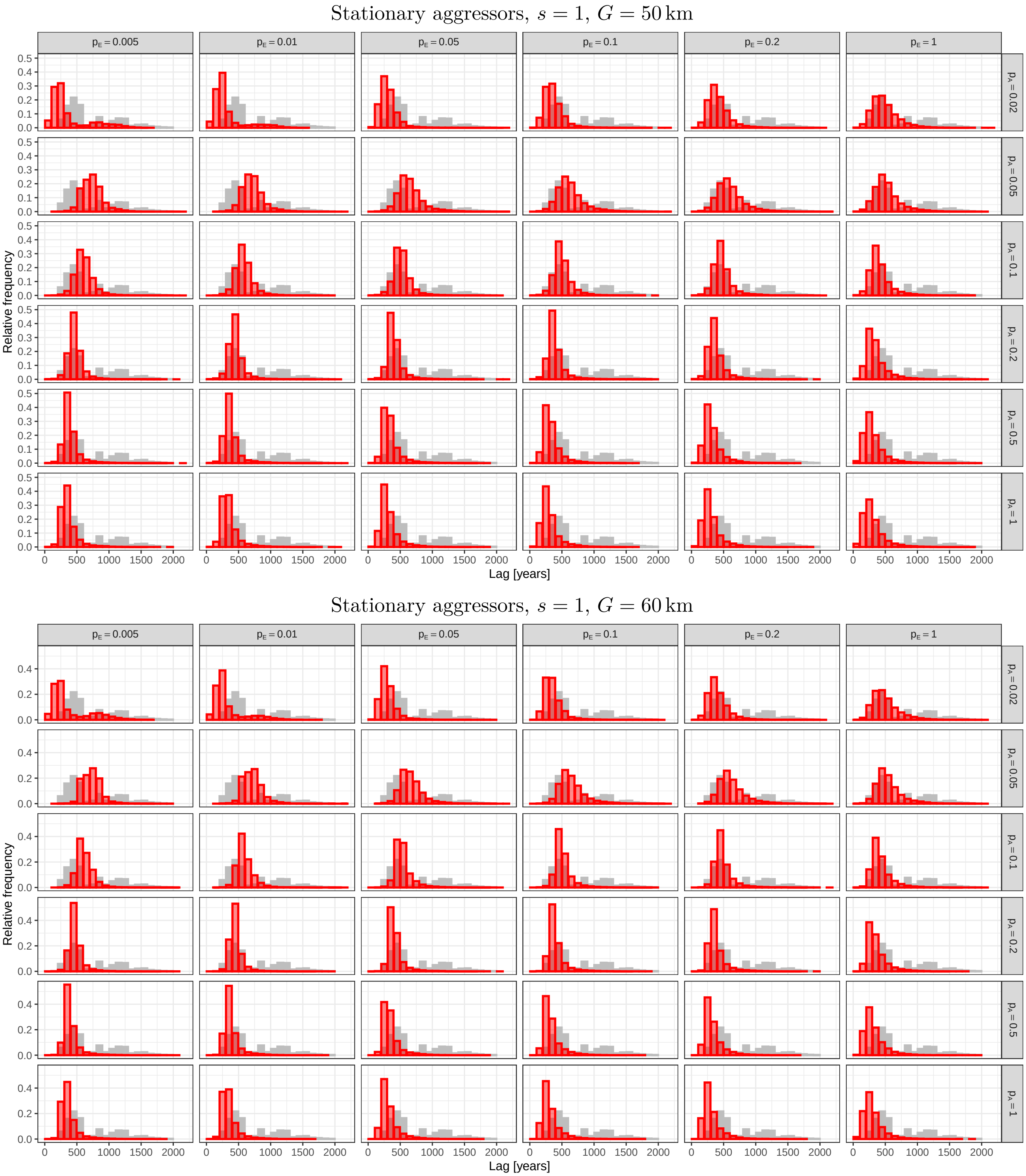

Figure S31: Distribution of the location of the first minimum in regional ACFs, model with stationary aggressors, $s = 1$, $G = 50$ and $60\,\mathrm{km}$ (top and bottom panels respectively).

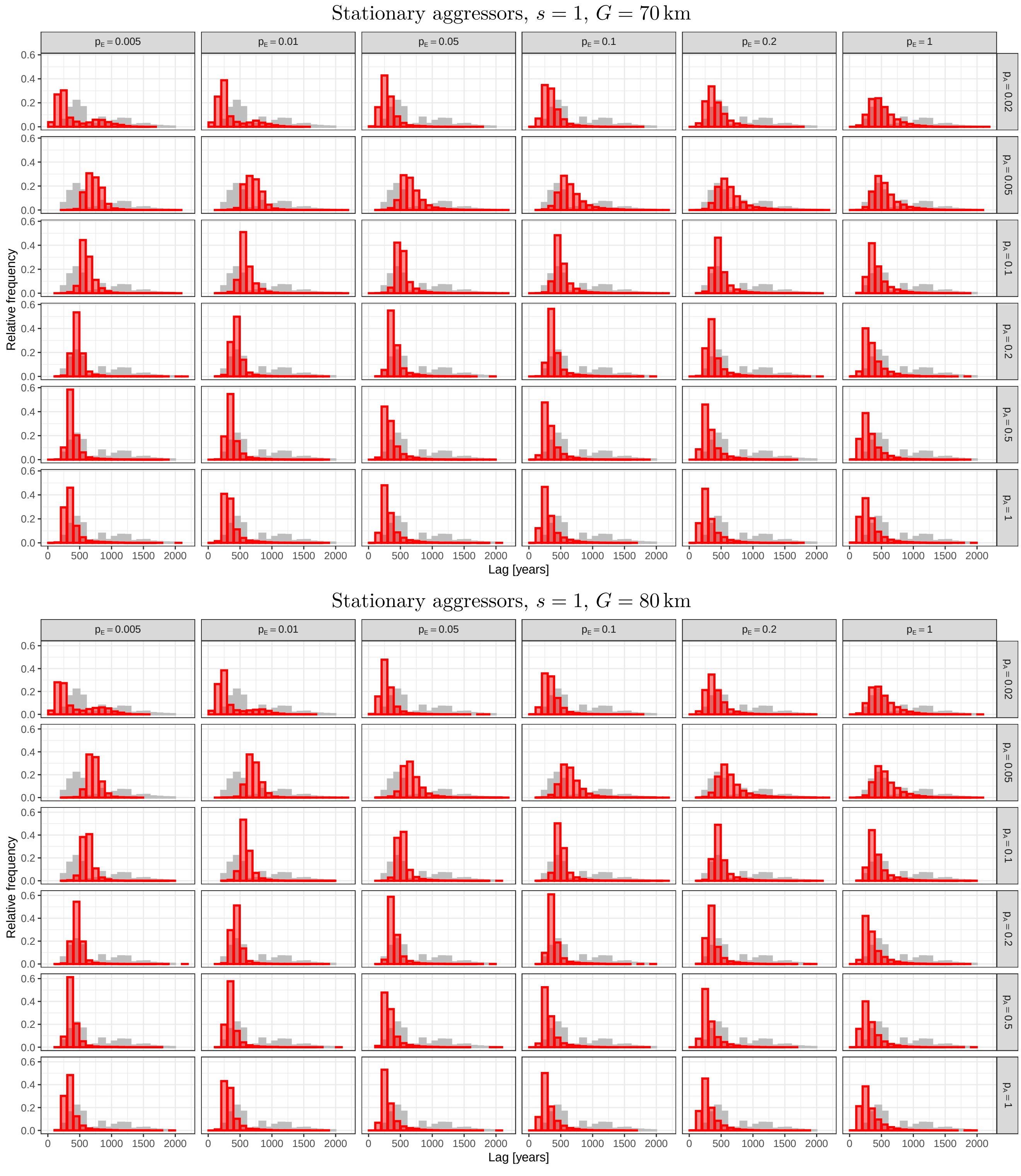

Figure S32: Distribution of the location of the first minimum in regional ACFs, model with stationary aggressors, $s = 1$, $G = 70$ and $80\,\mathrm{km}$ (top and bottom panels respectively).

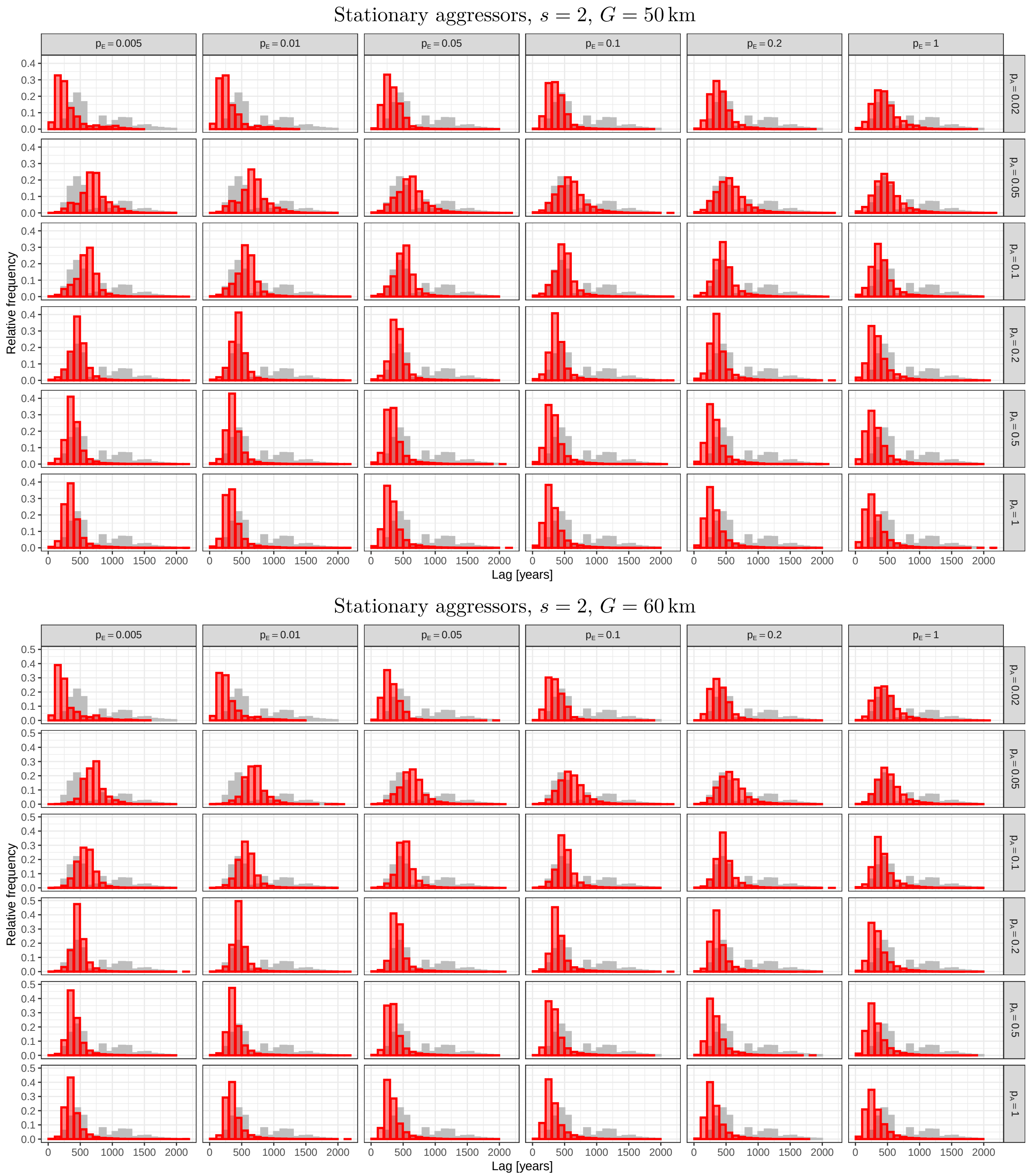

Figure S33: Distribution of the location of the first minimum in regional ACFs, model with stationary aggressors, $s = 2$, $G = 50$ and 60 km (top and bottom panels respectively).

Stationary aggressors, $s = 2$, $G = 70\,\mathrm{km}$

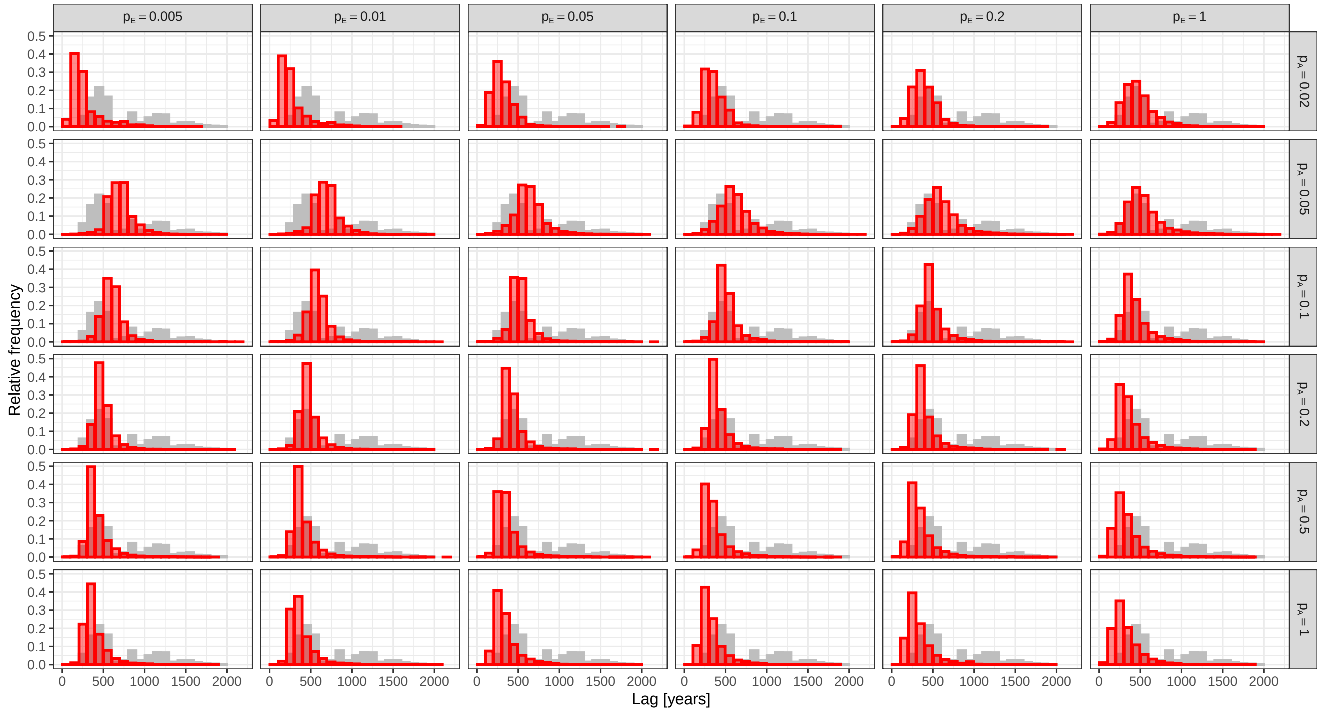

Stationary aggressors, $s = 2$, $G = 80\,\mathrm{km}$

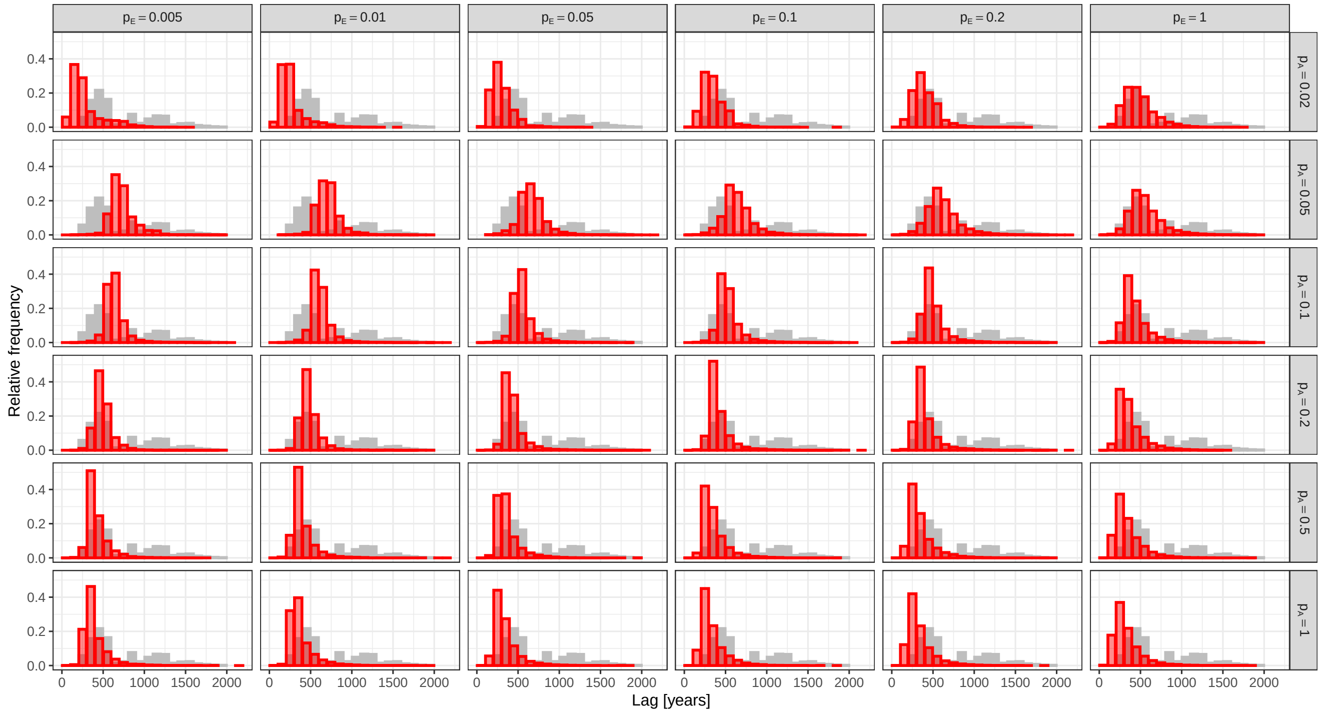

Figure S34: Distribution of the location of the first minimum in regional ACFs, model with stationary aggressors, $s = 2$, $G = 70$ and $80\,\mathrm{km}$ (top and bottom panels respectively).

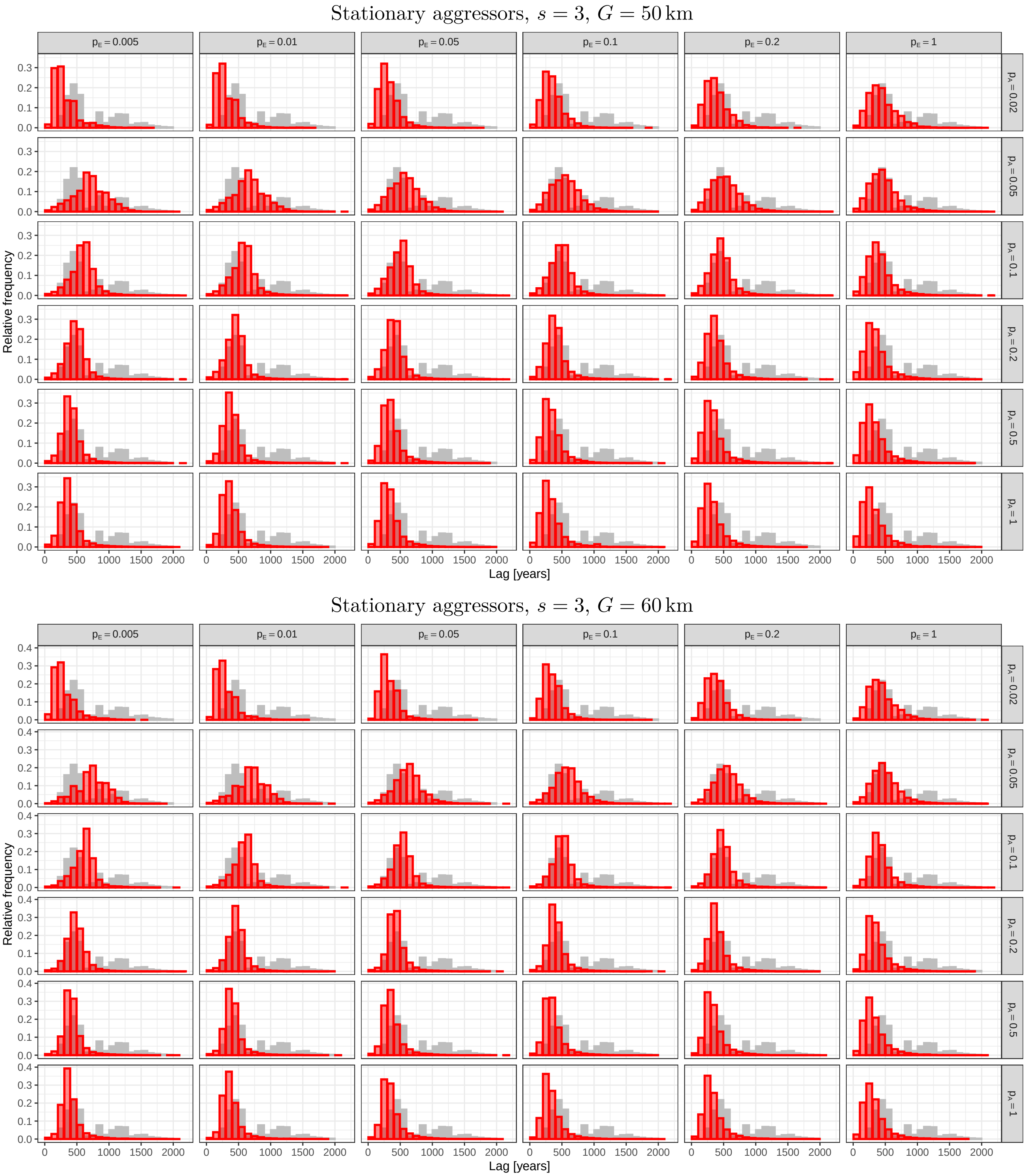

Figure S35: Distribution of the location of the first minimum in regional ACFs, model with stationary aggressors, $s = 3$, $G = 50$ and $60\,\text{km}$ (top and bottom panels respectively).

Stationary aggressors, $s = 3$, $G = 70\,\mathrm{km}$

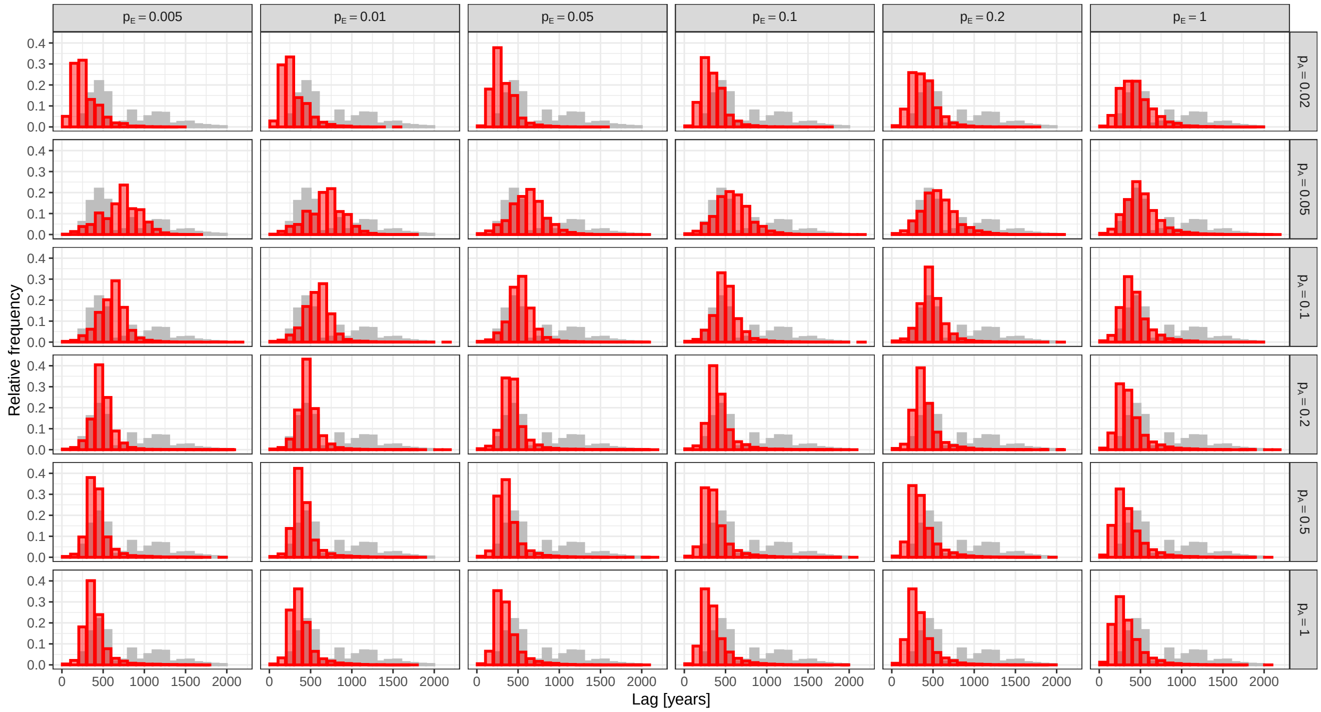

Stationary aggressors, $s = 3$, $G = 80\,\mathrm{km}$

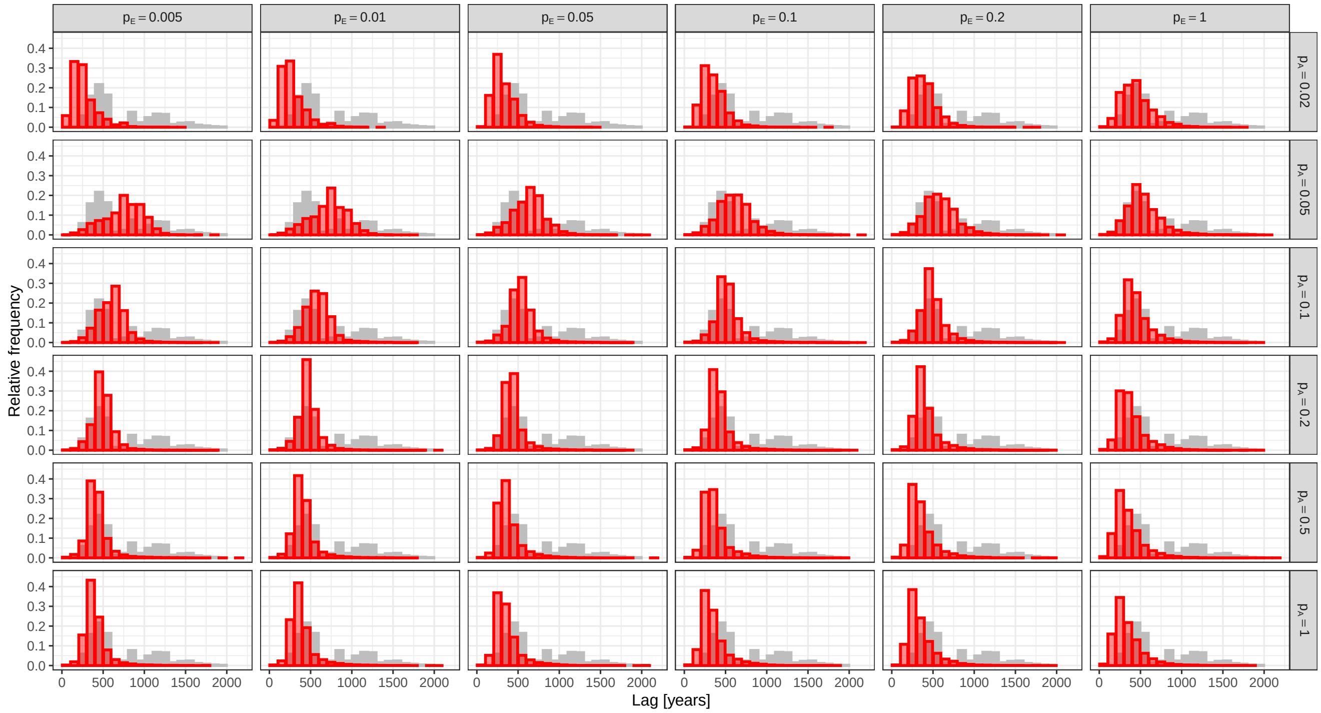

Figure S36: Distribution of the location of the first minimum in regional ACFs, model with stationary aggressors, $s = 3$, $G = 70$ and $80\,\mathrm{km}$ (top and bottom panels respectively).

Stationary aggressors, $s = 4$, $G = 50\,\text{km}$

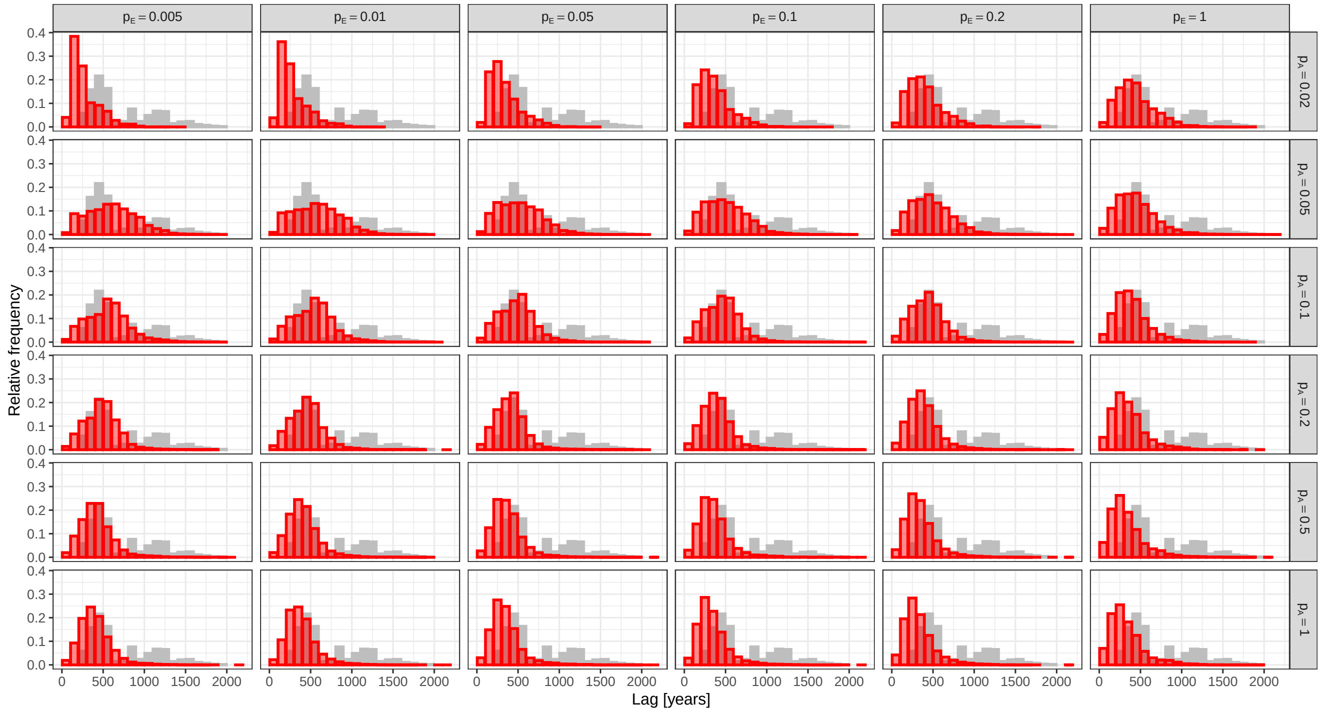

Stationary aggressors, $s = 4$, $G = 60\,\text{km}$

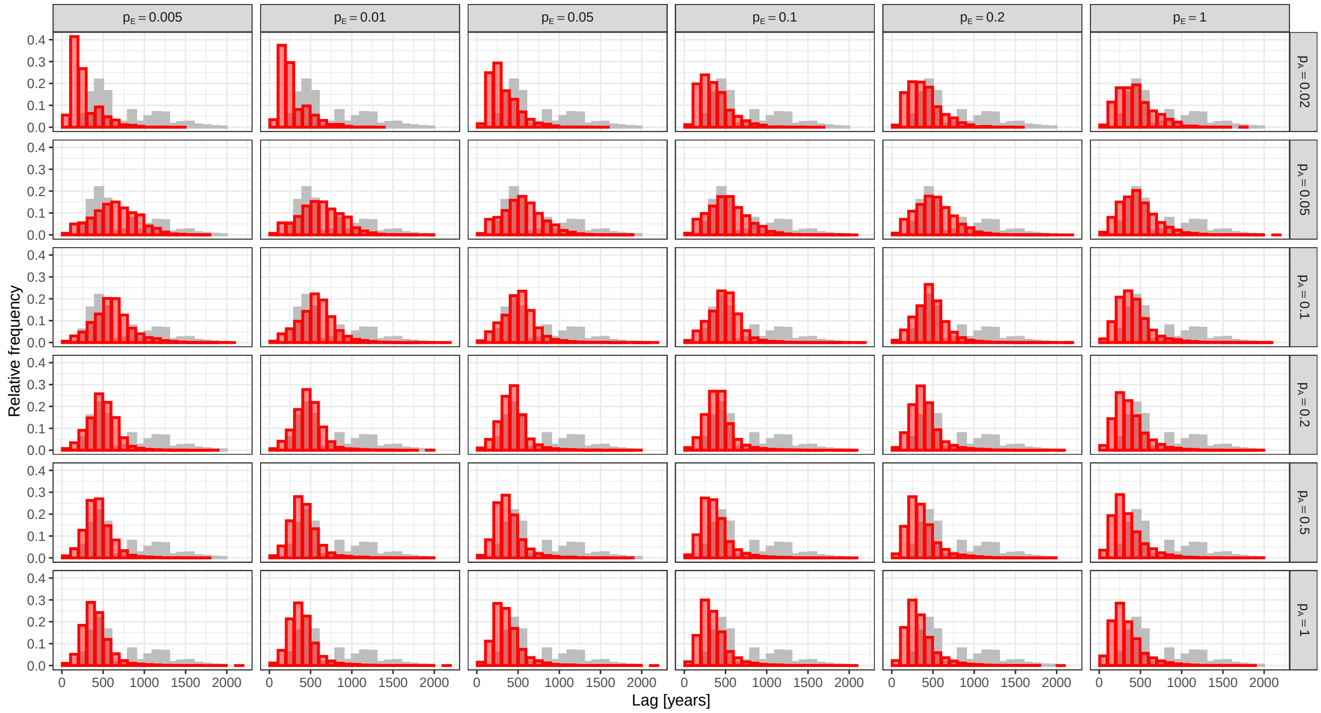

Figure S37: Distribution of the location of the first minimum in regional ACFs, model with stationary aggressors, $s = 4$, $G = 50$ and $60\,\text{km}$ (top and bottom panels respectively).

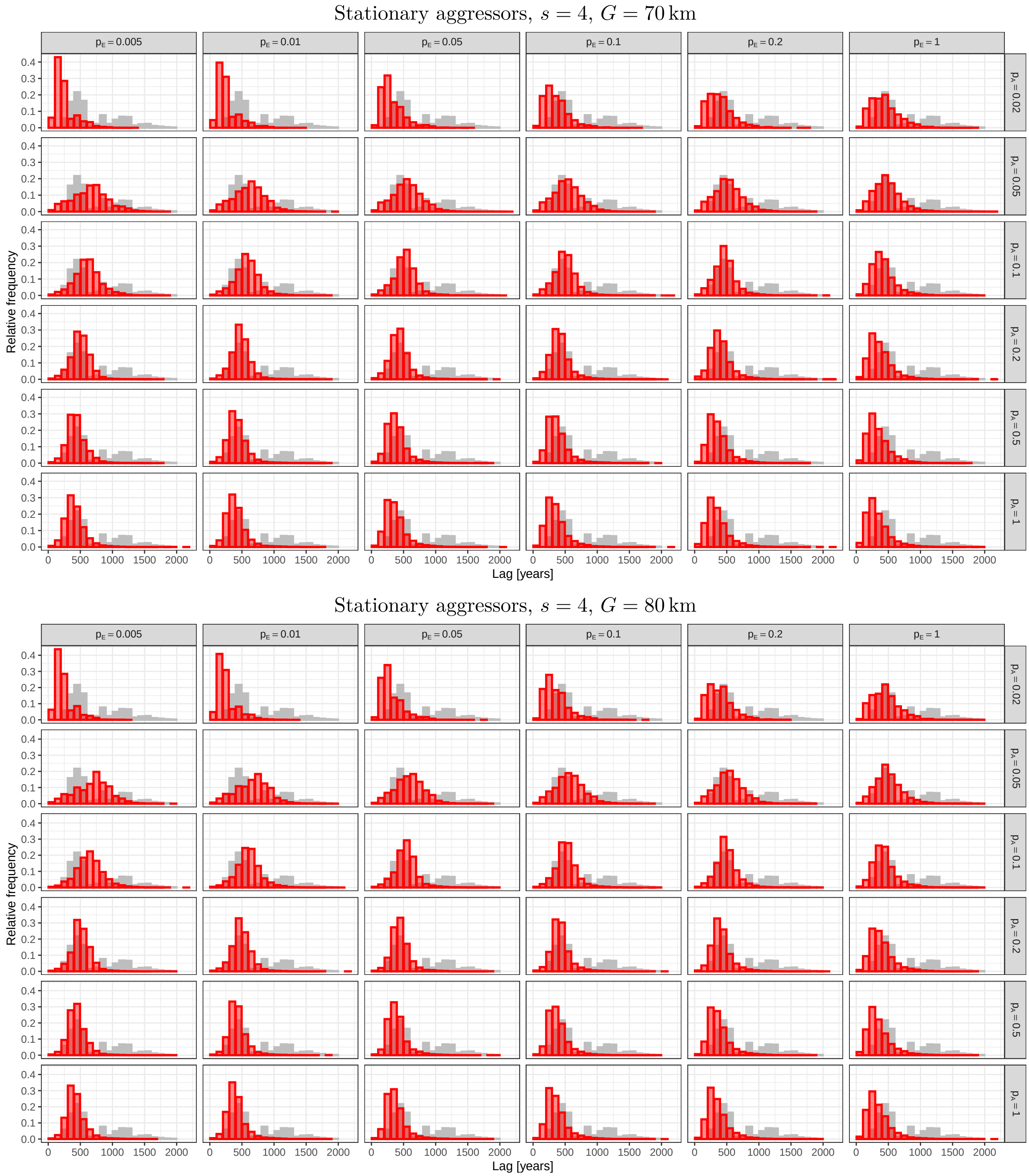

Figure S38: Distribution of the location of the first minimum in regional ACFs, model with stationary aggressors, $s = 4$, $G = 70$ and $80\,\mathrm{km}$ (top and bottom panels respectively).

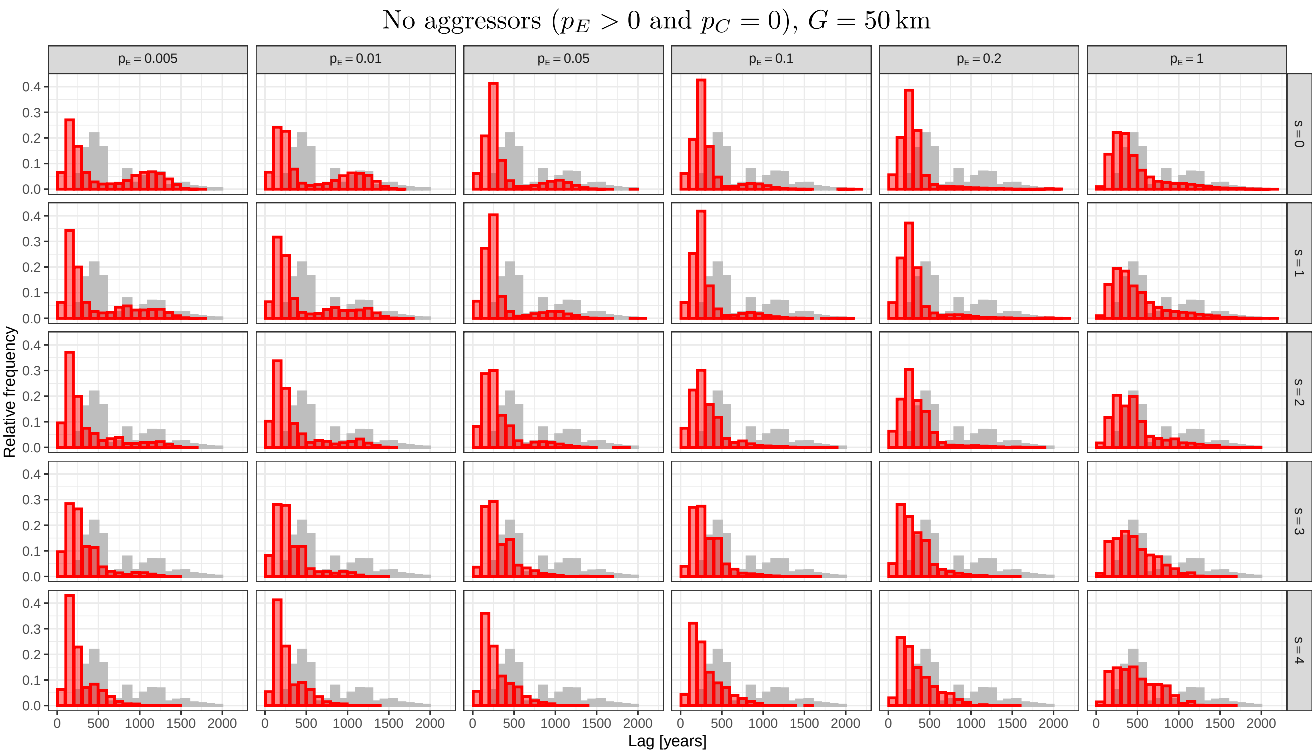

Figure S39: Distribution of the location of the first minimum in regional ACFs, model with conflict but no aggressors (i.e. first-order interactions), $G = 50\,\mathrm{km}$.

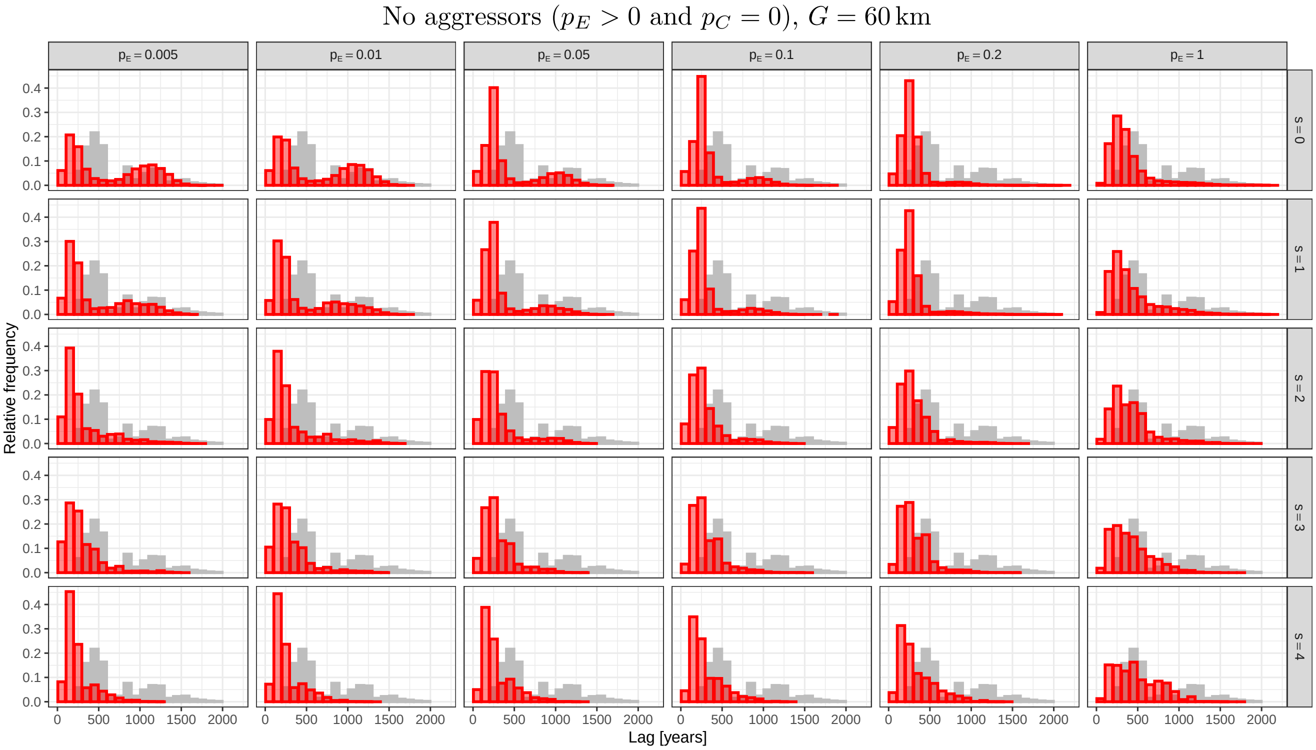

Figure S40: Distribution of the location of the first minimum in regional ACFs, model with conflict but no aggressors (i.e. first-order interactions), $G = 60\,\mathrm{km}$.

No aggressors ($p_E > 0$ and $p_C = 0$), $G = 70\,\mathrm{km}$

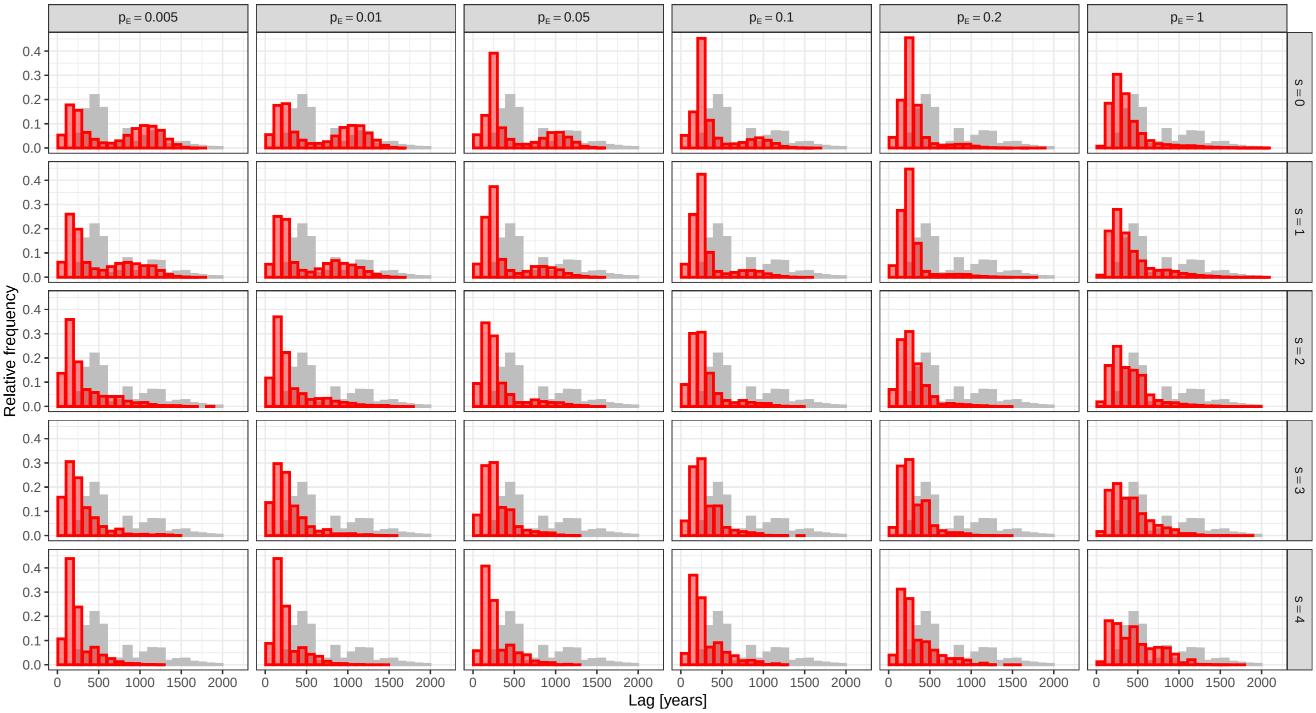

Figure S41: Distribution of the location of the first minimum in regional ACFs, model with conflict but no aggressors (i.e. first-order interactions), $G = 70\,\mathrm{km}$.

Bibliography

[1] Günther Fischer, Freddy O. Nachtergaele, Sylvia Prieler, Edmar Teixeira, Géza Tóth, Harrij van Velthuizen, Luc Verelst, and David Wiberg. Global Agro-ecological Zones (GAEZ v3.0). *IIASA and FAO*, page 196, 2008. ISSN 1944-9224.

[2] IIASA / FAO. Global Agro-ecological Zones (GAEZ v3.0). `https://www.gaez.iiasa.ac.at`, accessed 2021-06-17, 2012.

[3] José Luis Araus, Gustavo Ariel Slafer, Ramón Buxó, and Ignacio Romagosa. Productivity in prehistoric agriculture: Physiological models for the quantification of cereal yields as an alternative to traditional approaches. *Journal of Archaeological Science*, 30(6):681–693, 2003. ISSN 03054403. doi: 10.1016/S0305-4403(02)00240-6.

[4] José L. Araus, Juan P. Ferrio, Jordi Voltas, Mònica Aguilera, and Ramón Buxó. Agronomic conditions and crop evolution in ancient near east agriculture. *Nature Communications*, 5:4953, 2014. ISSN 20411723. doi: 10.1038/ncomms4953.

[5] Isabell Schmidt, Johanna Hilpert, Inga Kretschmer, Robin Peters, Manuel Broich, Sara Schiesberg, Oliver Vogels, Karl Peter Wendt, Andreas Zimmermann, and Andreas Maier. Approaching prehistoric demography: Proxies, scales and scope of the Cologne Protocol in European contexts: Approaching Prehistoric Demography. *Philosophical Transactions of the Royal Society B: Biological Sciences*, 376(1816), 2021. ISSN 14712970. doi: 10.1098/rstb.2019.0714rstb20190714.

[6] Peter Turchin, Thomas Currie, Christina Collins, Jill Levine, Oluwole Oyebamiji, Neil R. Edwards, Philip B. Holden, Daniel Hoyer, Kevin Feeney, Pieter François, and Harvey Whitehouse. An integrative approach to estimating productivity in past societies using Seshat: Global History Databank. *Holocene*, 31(6):1055–1065, 2021. ISSN 14770911. doi: 10.1177/0959683621994644.

[7] Tilman Baum, Claas Nendel, Stefanie Jacomet, Miquel Colobran, and Renate Ebersbach. "Slash and burn" or "weed and manure"? A modelling approach to explore hypotheses of late Neolithic crop cultivation in pre-alpine wetland sites. *Vegetation History and Archaeobotany*, 25(6):611–627, 2016. ISSN 09396314. doi: 10.1007/s00334-016-0583-x.

[8] Manfred Rösch, Harald Biester, Arno Bogenrieder, Eileen Eckmeier, Otto Ehrmann, Renate Gerlach, Mathias Hall, Christoph Hartkopf-Fröder, Ludger Herrmann, Birgit Kury, Jutta Lechterbeck, Wolfram Schier, and Erhard Schulz. Late Neolithic agriculture in temperate Europe-a long-term experimental approach. *Land*, 6(1):11, 2017. ISSN 2073445X. doi: 10.3390/land6010011.

[9] Karl Peter Wendt, Johanna Hilpert, and Andreas Zimmermann. Ein Modell zur Rekonstruktion von Landwirtschaftssystemen am Beispiel der Linearbandkeramik und der späten vorindustriellen Neuzeit. *Landschaftsarchäologie IV. Bericht der Römisch-Germanischen Kommission.*, 96(2015), 2019. doi: 10.11588/berrgk.2015.0.59354.

[10] Isabell Schmidt and Andreas Zimmermann. Population dynamics and socio-spatial organization of the Aurignacian: Scalable quantitative demographic data for western and central Europe. *PLoS ONE*, 14(2):1–20, 2019. ISSN 19326203. doi: 10.1371/journal.pone.0211562.

[11] Andreas Zimmermann, Johanna Hilpert, and Karl Peter Wendt. Estimations of population density for selected periods between the neolithic and AD 1800. *Human Biology*, 81(2-3):357–380, 2009. ISSN 00187143. doi: 10.3378/027.081.0313.

[12] Johannes Müller and Aleksandr Diachenko. Tracing long-term demographic changes: The issue of spatial scales. *PLoS ONE*, 14(1):e0208739, 2019. ISSN 19326203. doi: 10.1371/journal.pone.0208739.

[13] James A. Franke, Christoph Müller, Joshua Elliott, Alex C. Ruane, Jonas Jägermeyr, Juraj Balkovic, Philippe Ciais, Marie Dury, Pete D. Falloon, Christian Folberth, Louis François, Tobias Hank, Munir Hoffmann, R. Cesar Izaurralde, Ingrid Jacquemin, Curtis Jones, Nikolay Khabarov, Marian Koch, Michelle Li, Wenfeng Liu, Stefan Olin, Meridel Phillips, Thomas A.M. Pugh, Ashwan Reddy, Xuhui Wang, Karina Williams, Florian Zabel, and Elisabeth J. Moyer. The GGCMI Phase 2 emulators: global gridded crop model responses to changes in CO 2 , temperature, water, and nitrogen (version 1.0). *Geoscientific Model Development*, 13(5):3995–4018, 2020. doi: 10.5194/gmd-13-3995-2020.

[14] Edward Armstrong, Peter O. Hopcroft, and Paul J. Valdes. A simulated Northern Hemisphere terrestrial climate dataset for the past 60,000 years. *Scientific Data*, 6(1):1–16, 2019. ISSN 20524463. doi: 10.1038/s41597-019-0277-1. URL `http://dx.doi.org/10.1038/s41597-019-0277-1`.

[15] Werner Von Bloh, Sibyll Schaphoff, Christoph Müller, Susanne Rolinski, Katharina Waha, and Sönke Zaehle. Implementing the nitrogen cycle into the dynamic global vegetation, hydrology, and crop growth model LPJmL (version 5.0). *Geoscientific Model Development*, 11(7):2789–2812, 2018. ISSN 19919603. doi: 10.5194/gmd-11-2789-2018.

[16] James A. Franke, Christoph Müller, Joshua Elliott, Alex C. Ruane, Jonas Jägermeyr, Juraj Balkovic, Philippe Ciais, Marie Dury, Pete D. Falloon, Christian Folberth, Louis François, Tobias Hank, Munir Hoffmann, R. Cesar Izaurralde, Ingrid Jacquemin, Curtis Jones, Nikolay Khabarov, Marian Koch, Michelle Li, Wenfeng Liu, Stefan Olin, Meridel Phillips, Thomas A.M. Pugh, Ashwan Reddy, Xuhui Wang, Karina Williams, Florian Zabel, and Elisabeth J. Moyer. AgMIP's GGCMI Phase II: Crop model Emulators at 0.5 degree global resolution [Data set]. `http://doi.org/10.5281/zenodo.3994593`, accessed 2021-06-11, 2020.

[17] Alex C. Ruane, Richard Goldberg, and James Chryssanthacopoulos. Climate forcing datasets for agricultural modeling: Merged products for gap-filling and historical climate series estimation. *Agricultural and Forest Meteorology*, 200:233–248, 2015. ISSN 01681923. doi: 10.1016/j.agrformet.2014.09.016. URL `http://dx.doi.org/10.1016/j.agrformet.2014.09.016`.

[18] NASA. AgMERRA dataset. `https://data.giss.nasa.gov/impacts/agmipcf/`, accessed 2021-06-09, 2015.

[19] EPA. Climate Change Indicators: Atmospheric Concentrations of Greenhouse Gases. `https://www.epa.gov/climate-indicators/climate-change-indicators-atmospheric-concentrations-greenhouse-gases`, accessed 2021-06-24, 2021.

[20] Detlef Gronenborn, Barbara Horejs, Mario Börner, and Michale Ober. Expansion of farming in western Eurasia, 9600 - 4000 cal BC (update vers. 2021.2), 2021. URL `https://zenodo.org/record/5903165#.YlSOU1yxVEZ`.

[21] Sean S. Downey, W. Randall Haas, and Stephen J. Shennan. European Neolithic societies showed early warning signals of population collapse. *Proceedings of the National Academy of Sciences of the United States of America*, 113(35):9751–9756, 2016. ISSN 10916490. doi: 10.1073/pnas.1602504113.

[22] Enrico R. Crema and Andrew Bevan. Inference from large sets of radiocarbon dates: Software and methods. *Radiocarbon*, 63(1):23–39, 2021. ISSN 00338222. doi: 10.1017/RDC.2020.95.

[23] Gianmarco Alberti. Modeling Group Size and Scalar Stress by Logistic Regression from an Archaeological Perspective. *PLoS ONE*, 9(3):e91510, March 2014. ISSN 1932-6203. doi: 10.1371/journal.pone.0091510.
